# Supplementary material for: Activation of the Rat P2X7 Receptor by Functionally Different ATP Activation Sites
Source: Cells. 2025 Jun 6;14(12):855. doi: 10.3390/cells14120855 (PMC12191118; doi:10.3390/cells14120855)
Supplement: Supplementary file 1 [file cells-14-00855-s001.zip › Figure S1 revised 2025_06_05.pdf]

Figure S1 (related to Fig. 3). Quantification profiles of rP2X7 concatamers and their cleavage products

Fig. S1A

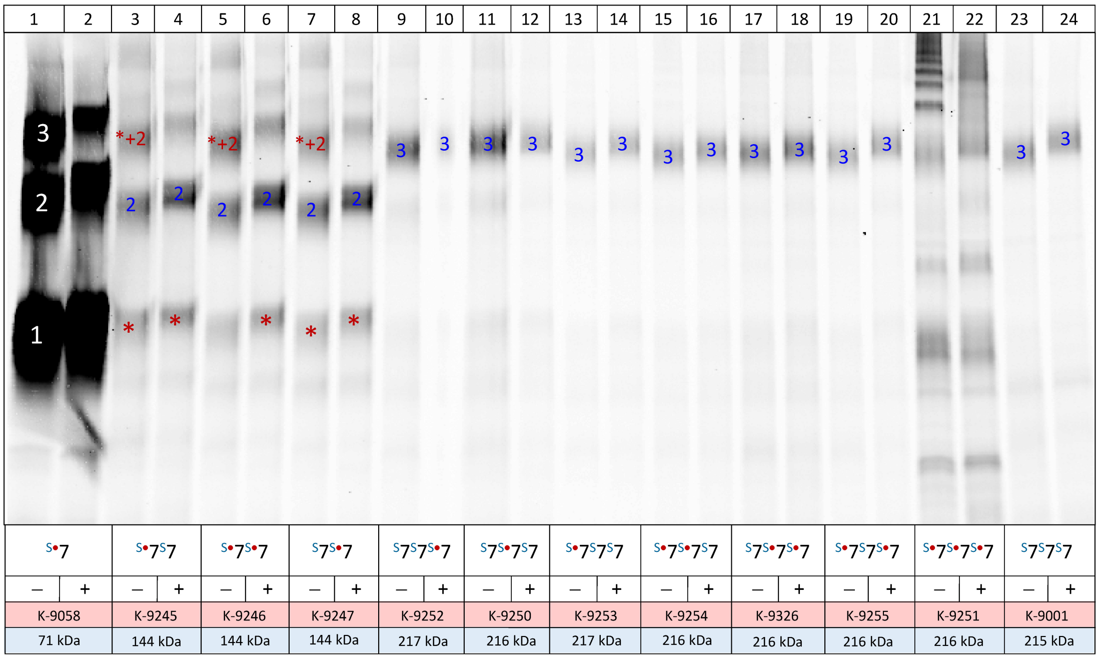

Fig. S1B

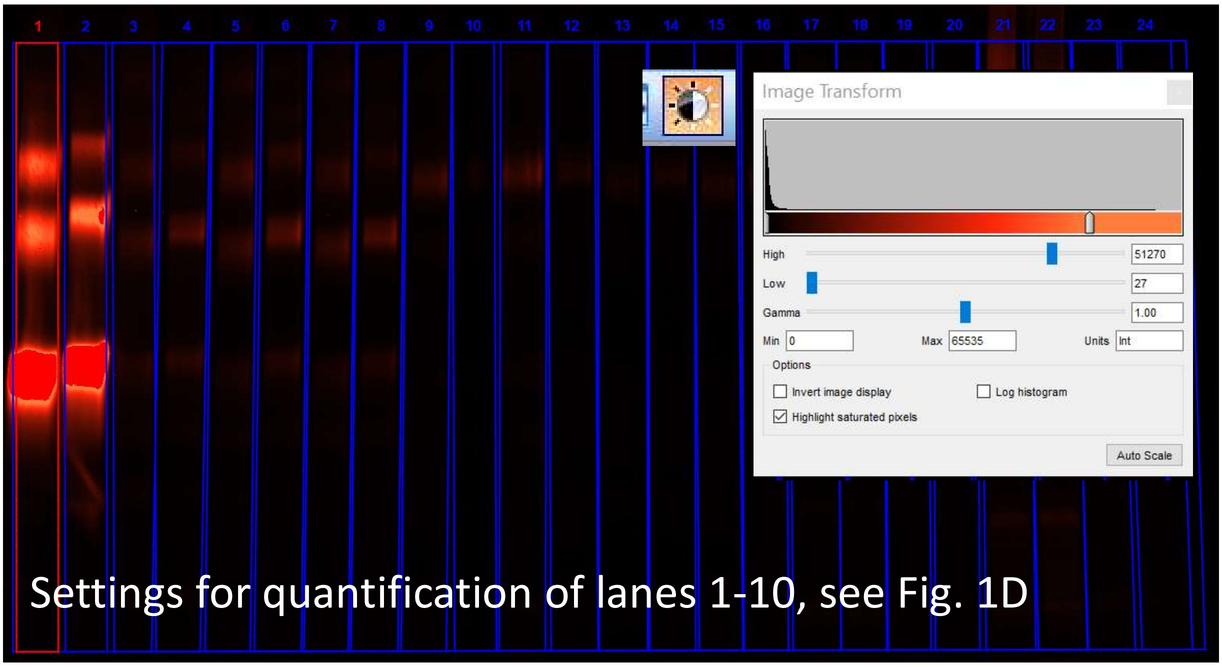

Fig. S1C

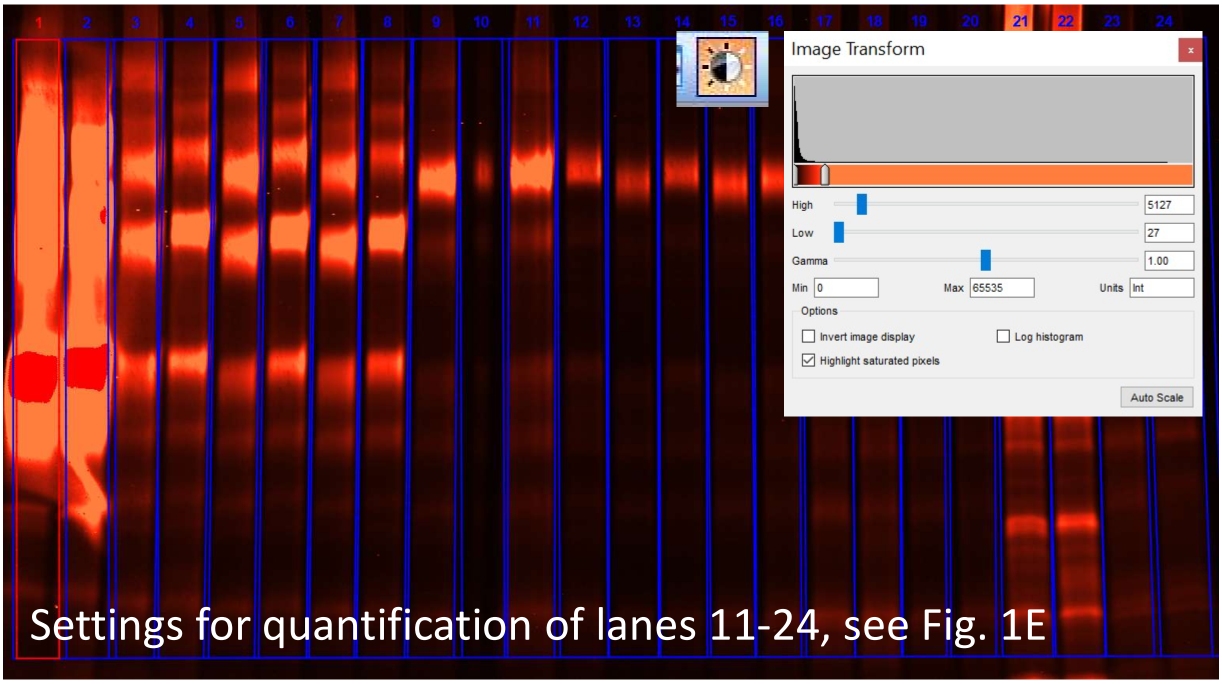

Fig. S1D

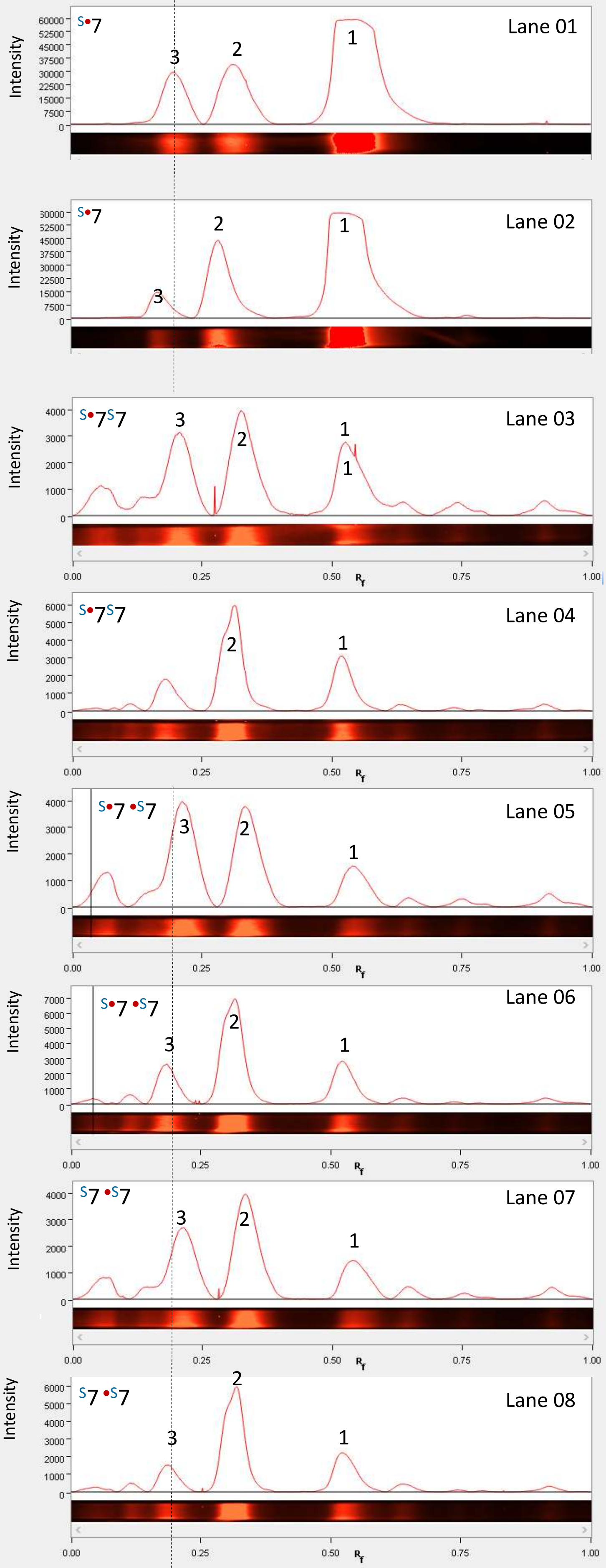

Figure S1 (related to Fig. 3). Quantification Profiles of rP2X7 Concatamers and Their Cleavage Products. The SDS-PAGE gel shown as Fig. 3 in the manuscript is also shown here to illustrate quantified profiles of the data. The wet gel was scanned for IR 800 fluorescence using a LI-COR scanner at 800 nm. The scan was quantified with Image Lab 6.01 (Bio-Rad Laboratories) using the Lane Profile command with the following settings for lanes 1-2: High 51270, Low 27, Gamma 1.0 and lanes 3-24: High 512700, Low 27, Gamma 1.0. Lanes 1 and 2 were overexposed, while lanes 21 and 22 contained leaky oocytes. The odd numbers refer to samples that were incubated without DTT, while the even numbers refer to samples that were incubated with 20 mM DTT.

Fig. S1E

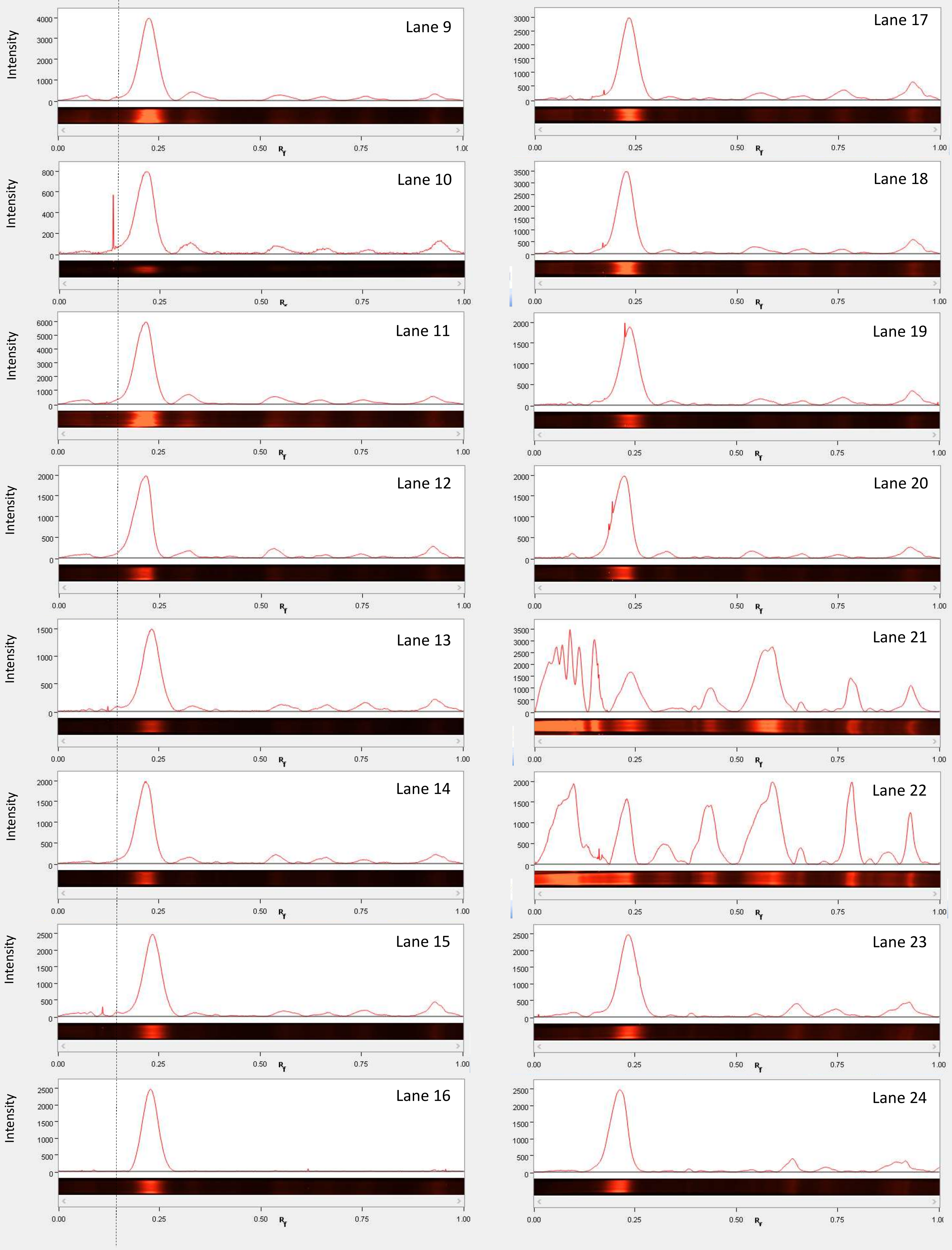
Intensity
Rf
Lane 9
Lane 10
Lane 11
Lane 12
Lane 13
Lane 14
Lane 15
Lane 16
Lane 17
Lane 18
Lane 19
Lane 20
Lane 21
Lane 22
Lane 23
Lane 24
0.00
0.25
0.50
0.75
1.00
