# Supplementary material for: Activation of the Rat P2X7 Receptor by Functionally Different ATP Activation Sites
Source: Cells. 2025 Jun 6;14(12):855. doi: 10.3390/cells14120855 (PMC12191118; doi:10.3390/cells14120855)
Supplement: Supplementary file 1 [file cells-14-00855-s001.zip › Markwardt et al Suppl Table 1 (Vector sequences).pdf]

Supplementary Table S1 - **Complete annotated plasmid sequences of the rP2X4 and rP2X7 concatamer constructs**. The complete plasmid sequences are shown, with the following features highlighted: start codon (green), stop codon (red), K^{64}A mutation (magenta), S3 double tag including linker (turquoise), remainder of the coding region (grey), SP6 promoter (yellow), and vector-encoded poly(A) sequence (bold). The inclusion of a long poly(A) stretch (~ 100 adenosines and longer) in the vector is crucial for efficient cRNA-driven expression in *X. laevis* oocytes for biochemical experiments, as previously demonstrated (Gloor, Pongs, Schmalzing. Gene, 160: 213-217, 1995 doi: 10.1016/0378-1119(95)00226-v.). The construct numbers given are laboratory identifiers and are also included in Figur 3 to allow cross-referencing of rP2X7 constructs with their coding sequences. Color code: SP6 primer yellow; start codon green; stop codon red, K64A mutation magenta, S3 double tag turquoise, rest of coding region in grey, **poly A sequence in bold**.

>K-9080 ^{S3}rP2X4^{S3}rP2X4^{S3}rP2X4 ($^{wt-wt-wt}$trimer) 141.280 Da, pI 8.01, 1.277 residues

AATACAAGCTTACAACAAAGAACAACAAACAACAAAGTCCGACGTCGAAGTAGCCACCATGGGGAGCGCTTGGAG
CCACCCGCAGTTCGAAAAAGGTGGAGGTTCTGGCGGTGGATCGGGAGGTTCAGCGTGGAGCCACCCGCAGTTCGA
GAAAGCGGGCTGCTGCTCCGTGCTCGGGTCCTTCCTGTTCGAGTACGACACGCCGCGCATCGTGCTCATCCGCAG
CCGTAAAGTGGGGCTGATGAACCGCGCGGTGCAGCTGCTCATCCTGGCTTACGTCATCGGGTGGGTGTTCGTGTG
GGAAAAGGGCTACCAGGAAACGGACTCCGTGGTCAGCTCGGTGACAACCAAAGCCAAAGGTGTGGCTGTGACCAA
CACCTCTCAGCTTGGATTCCGGATCTGGGACGTGGCGGACTATGTGATTCCAGCTCAGGAGGAAAACTCCCTCTT
CATTATGACCAACATGATTGTCACCGTGAACCAGACACAGAGCACCTGTCCAGAGATTCCTGATAAGACCAGCAT
TTGTAATTCAGACGCCGACTGCACTCCTGGCTCCGTGGACACCCACAGCAGTGGAGTTGCGACTGGAAGATGTGT
TCCTTTCAATGAGTCTGTGAAGACCTGTGAGGTGGCTGCATGGTGTCCAGTGGAGAACGACGTTGGCGTGCCAAC
GCCGGCTTTCTTAAAGGCTGCAGAAAACTTCACCCTCTTGGTAAAGAACAACATCTGGTACCCCAAGTTTAACTT
CAGCAAGAGGAACATCCTCCCCAACATCACCACGTCCTACCTCAAATCGTGCATTTACAATGCTCAAACGGATCC
CTTCTGCCCCATATTCCGTCTTGGCACAATCGTGGAGGACGCGGGACATAGCTTCCAGGAGATGGCAGTTGAGGG
AGGCATCATGGGTATCCAGATCAAGTGGGACTGCAATCTGGATAGAGCCGCCTCCCTTTGCCTGCCCAGATATTC
CTTCCGGCGCCTGGACACCCGGGACCTGGAACACAATGTGTCTCCTGGCTACAATTTCAGGTTTGCCAAGTACTA
CAGGGACCTGGCCGCCAAAGAGCAGCGCACACTCACCAAGGCGTACGGCATCCGCTTTGACATCATCGTGTTTGG
AAAGGCTGGGAAGTTTGACATCATCCCTACCATGATCAATGTTGGCTCTGGCTTGGCGCTCCTCGGGGTGGCGAC
GGTGCTCTGTGACGTCATAGTCCTCTACTGCATGAAGAAGAAATACTACTACCGGGACAAGAAATATAAGTATGT
GGAAGACTACGAGCAGGGTCTTTCGGGGGAGATGAACCAGGCTGGAAGCGCAGGCAGCGCCGGTTCTCTCATGGG
GAGCGCTTGGAGCCACCCGCAGTTCGAAAAAGGTGGAGGTTCTGGCGGTGGATCGGGAGGTTCAGCGTGGAGCCA
CCCGCAGTTCGAGAAAGCGGGCTGCTGCTCCGTGCTCGGGTCCTTCCTGTTCGAGTACGACACGCCGCGCATCGT
GCTCATCCGCAGCCGTAAAGTGGGGCTGATGAACCGCGCGGTGCAGCTGCTCATCCTGGCTTACGTCATCGGGTG
GGTGTTCGTGTGGGAAAAGGGCTACCAGGAAACGGACTCCGTGGTCAGCTCGGTGACAACCAAAGCCAAAGGTGT
GGCTGTGACCAACACCTCTCAGCTTGGATTCCGGATCTGGGACGTGGCGGACTATGTGATTCCAGCTCAGGAGGA
AAACTCCCTCTTCATTATGACCAACATGATTGTCACCGTGAACCAGACACAGAGCACCTGTCCAGAGATTCCTGA
TAAGACCAGCATTTGTAATTCAGACGCCGACTGCACTCCTGGCTCCGTGGACACCCACAGCAGTGGAGTTGCGAC
TGGAAGATGTGTTCCTTTCAATGAGTCTGTGAAGACCTGTGAGGTGGCTGCATGGTGTCCAGTGGAGAACGACGT
TGGCGTGCCAACGCCGGCTTTCTTAAAGGCTGCAGAAAACTTCACCCTCTTGGTAAAGAACAACATCTGGTACCC
CAAGTTTAACTTCAGCAAGAGGAACATCCTCCCCAACATCACCACGTCCTACCTCAAATCGTGCATTTACAATGC
TCAAACGGATCCCTTCTGCCCCATATTCCGTCTTGGCACAATCGTGGAGGACGCGGGACATAGCTTCCAGGAGAT
GGCAGTTGAGGGAGGCATCATGGGTATCCAGATCAAGTGGGACTGCAATCTGGATAGAGCCGCCTCCCTTTGCCT
GCCCAGATATTCCTTCCGGCGCCTGGACACCCGGGACCTGGAACACAATGTGTCTCCTGGCTACAATTTCAGGTT
TGCCAAGTACTACAGGGACCTGGCCGCCAAAGAGCAGCGCACACTCACCAAGGCGTACGGCATCCGCTTTGACAT
CATCGTGTTTGGAAAGGCTGGGAAGTTTGACATCATCCCTACCATGATCAATGTTGGCTCTGGCTTGGCGCTCCT
CGGGGTGGCGACGGTGCTCTGTGACGTCATAGTCCTCTACTGCATGAAGAAGAAATACTACTACCGGGACAAGAA
ATATAAGTATGTGGAAGACTACGAGCAGGGTCTTTCGGGGGAGATGAACCAGGCTGGAAGCGCAGGCAGCGCCGG
TTCTCTCATGGGGAGCGCTTGGAGCCACCCGCAGTTCGAAAAAGGTGGAGGTTCTGGCGGTGGATCGGGAGGTTC
AGCGTGGAGCCACCCGCAGTTCGAGAAAGCGGGCTGCTGCTCCGTGCTCGGGTCCTTCCTGTTCGAGTACGACAC
GCCGCGCATCGTGCTCATCCGCAGCCGTAAAGTGGGGCTCATGAACCGCGCGGTGCAGCTGCTCATCCTGGCTTA
CGTCATCGGGTGGGTGTTCGTGTGGGAAAAGGGCTACCAGGAAACGGACTCCGTGGTCAGCTCGGTGACAACCAA
AGCCAAAGGTGTGGCTGTGACCAACACCTCTCAGCTTGGATTCCGGATCTGGGACGTGGCGGACTATGTGATTCC
AGCTCAGGAGGAAAACTCCCTCTTCATTATGACCAACATGATTGTCACCGTGAACCAGACACAGAGCACCTGTCC

AGAGATTCCTGATAAGACCAGCATTTGTAATTCAGACGCCGACTGCACTCCTGGCTCCGTGGACACCCACAGCAG
TGGAGTTGCGACTGGAAGATGTGTTCCTTTCAATGAGTCTGTGAAGACCTGTGAGGTGGCTGCATGGTGTCCAGT
GGAGAACGACGTTGGCGTGCCAACGCCGGCTTTCTTAAAGGCTGCAGAAAACTTCACCCTCTTGGTAAAGAACAA
CATCTGGTACCCCAAGTTTAACTTCAGCAAGAGGAACATCCTCCCCAACATCACCACGTCCTACCTCAAATCGTG
CATTTACAATGCTCAAACGGATCCCTTCTGCCCCATATTCCGTCTTGGCACAATCGTGGAGGACGCGGGACATAG
CTTCCAGGAGATGGCAGTTGAGGGAGGCATCATGGGTATCCAGATCAAGTGGGACTGCAATCTGGATAGAGCCGC
CTCCCTTTGCCTGCCCAGATATTCCTTCCGGCGCCTGGACACCCGGGACCTGGAACACAATGTGTCTCCTGGCTA
CAATTTCAGGTTTGCCAAGTACTACAGGGACCTGGCCGCCAAAGAGCAGCGCACACTCACCAAGGCGTACGGCAT
CCGCTTTGACATCATCGTGTTTGGAAAGGCTGGGAAGTTTGACATCATCCCTACCATGATCAATGTTGGCTCTGG
CTTGGCGCTCCTCGGGGTGGCGACGGTGCTCTGTGACGTCATAGTCCTCTACTGCATGAAGAAGAAATACTACTA
CCGGGACAAGAAATATAAGTATGTGGAAGACTACGAGCAGGGTCTTTCGGGGGAGATGAACCAGTGACGCCTCTA
GAATTCCCGGGCGCGCCGTCGACGTAGCTTTCATTTGTTTT**AA**TTT**A**TTTTTT**AAA**T**A**GC**A**TT**A**C**AAAA**C**AAA**TT
ACC**A**C**AA**C**AAAAAAAAAA**CC**AAAA**C**AAAAA**C**AAA**T**AA**T**AAA**GTCCC**AAAA**C**AAAAA**CGG**AA**T**A**TGC**AAA**C**AAAAA**
AAAAAAAAAAAAAAAAAAAAAAAAAAAAAAAAAAAAAAAAAAAAAAAAAGAATTGCTCGAGCGGCCGCTCGAGCAATT
CCGGTCTCCCTATAGTGAGTCGTATTAGGCGTAATAGCGAAGAGGCCCGCACCGATCGCCCTTTCCAACAGTTGC
GCAGCCTGAATGGCGAATGGGACGCGCCCTGTAGCGGCGCATTAAGCGCGGCGGGTGTGGTGGTTACGCGCAGCG
TGACCGCTACACTTGCCAGCGCCCTAGCGCCCGCTCCTTTCGCTTTCTTCCCTTCCTTTCTCGCCACGTTCGCCG
GCTTTCCCCGTCAAGCTCTAAATCGGGGGCTCCCTTTAGGGTTCCGATTTAGTGCTTTACGGCACCTCGACCCCA
AAAAACTTGATTAGGGTGATGGTTCACGTGGGCCATCGCCCTGATAGACGGTTTTTCGCCCTTTGACGTTGGAGT
CCACGTTCTTTAATAGTGGACTCTTGTTCCAAACTGGAACAACACTCAACCCTATCTCGGTCTATTCTTTTGATT
TATAAGGGATTTTGCCGATTTCGGCCTATTGGTTAAAAAATGAGCTGATTTAACAAAAATTTAACGCGAATTTTAA
CAAAATATTAACGTTTACAATTTCAGGTGGCACTTTTCGGGGAAATGTGCGCGGAACCCCTATTTGTTTATTTTT
CTAAATACATTCAAATATGTATCCGCTCATGAGACAATAACCCTGATAAATGCTTCAATAATATTGAAAAAGGAA
GAGTATGAGTATTCAACATTTCCGTGTCGCCCTTATTCCCTTTTTTGCGGCATTTTGCCTTCCTGTTTTTGCTCA
CCCAGAAACGCTGGTGAAAGTAAAAGATGCTGAAGATCAGTTGGGTGCACGAGTGGGTTACATCGAACTGGATCT
CAACAGCGGTAAGATCCTTGAGAGTTTTCGCCCCGAAGAACGTTTTCCAATGATGAGCACTTTTAAAGTTCTGCT
ATGTGGCGCGGTATTATCCCGTATTGACGCCGGGCAAGAGCAACTCGGTCGCCGCATACACTATTCTCAGAATGA
CTTGGTTGAGTACTCACCAGTCACAGAAAAGCATCTTACGGATGGCATGACAGTAAGAGAATTATGCAGTGCTGC
CATAACCATGAGTGATAACACTGCGGCCAACTTACTTCTGACAACGATCGGAGGACCGAAGGAGCTAACCGCTTT
TTTGCACAACATGGGGGATCATGTAACTCGCCTTGATCGTTGGGAACCGGAGCTGAATGAAGCCATACCAAACGA
CGAGCGTGACACCACGATGCCTGTAGCAATGGCAACAACGTTGCGCAAACTATTAACTGGCGAACTACTTACTCT
AGCTTCCCGGCAACAATTAATAGACTGGATGGAGGCGGATAAAGTTGCAGGACCACTTCTGCGCTCGGCCCTTCC
GGCTGGCTGGTTTATTGCTGATAAATCTGGAGCCGGTGAGCGTGGGTCTCGCGGTATCATTGCAGCACTGGGGCC
AGATGGTAAGCCCTCCCGTATCGTAGTTATCTACACGACGGGGAGTCAGGCAACTATGGATGAACGAAATAGACA
GATCGCTGAGATAGGTGCCTCACTGATTAAGCATTGGTAACTGTCAGACCAAGTTTACTCATATATACTTTAGAT
TGATTTAAAACTTCATTTTTAATTTAAAAGGATCTAGGTGAAGATCCTTTTTGATAATCTCATGACCAAAATCCC
TTAACGTGAGTTTTCGTTCCACTGAGCGTCAGACCCCGTAGAAAAGATCAAAGGATCTTCTTGAGATCCTTTTTT
TCTGCGCGTAATCTGCTGCTTGCAAACAAAAAAACCACCGCTACCAGCGGTGGTTTGTTTGCCGGATCAAGAGCT
ACCAACTCTTTTTCCGAAGGTAACTGGCTTCAGCAGAGCGCAGATACCAAATACTGTCCTTCTAGTGTAGCCGTA
GTTAGGCCACCACTTCAAGAACTCTGTAGCACCGCCTACATACCTCGCTCTGCTAATCCTGTTACCAGTGGCTGC
TGCCAGTGGCGATAAGTCGTGTCTTACCGGGTTGGACTCAAGACGATAGTTACCGGATAAGGCGCAGCGGTCGGG
CTGAACGGGGGGTTCGTGCACACAGCCCAGCTTGGAGCGAACGACCTACACCGAACTGAGATACCTACAGCGTGA
GCATTGAGAAAGCGCCACGCTTCCCGAAGGGAGAAAGGCGGACAGGTATCCGGTAAGCGGCAGGGTCGGAACAGG
AGAGCGCACGAGGGAGCTTCCAGGGGGAAACGCCTGGTATCTTTATAGTCCTGTCGGGTTTCGCCACCTCTGACT
TGAGCGTCGATTTTTGTGATGCTCGTCAGGGGGGCGGAGCCTATGGAAAAACGCCAGCAACGCGGCCTTTTTACG
GTTCCTGGCCTTTTGCTGGCCTTTTGCTCACATGTTCTTTCCTGCGTTATCCCCTGATTCTGTGGATAACCGTAT
TACCGCCTTTGAGTGAGCTGATACCGCTCGCCGCAGCCGAACGACCGAGCGCAGCGAGTCAGTGAGCGAGGAAGC
GGAAGAGCGCCCAATACGCAAACCGCCTCTCCCCGCGCGTTGGCCGATTCATTAATGCAGTAATACATAACCTTA
TGTATCATACACATACGATTTAGGTGACACTATAG

>K-9058 ^{S3K64A}rP2X7 (^{ko}monomer) 71.489 Da, pI 8.21, 627 residues

AATACAAGCTTACAACAAAGAACAACAAACAACAAAGTCCGACGTCGAAGTAGCCACCTACCATCATCATCATCA
TCCCATGGGGAGCGCTTGGAGCCACCCGCAGTTCGAAAAAGGTGGAGGTTCTGGCGGTGGATCGGGAGGTTCAGC
GTGGAGCCACCCGCAGTTCGAGAAAGCTCCCGCTTGCTGTAGCTGGAACGATGTCTTTCAGTATGAGACAAACAA
AGTCACCCGGATCCAGAGCGTGAATTACGGCACCATCAAGTGGATCTTGCACATGACAGTCTTTTCCTACGTTAG
CTTTGCTTTGATGAGCGACAAGCTATATCAGCGGAAGGAGCCCCTTATCAGCTCTGTGCACACCGCTGTCAAAGG
CGTTGCAGAGGTGACAGAGAATGTCACGGAGGGCGGGGTGACGAAGTTAGTACACGGCATCTTCGACACGGCCGA
CTACACCCTCCCTTTGCAGGGGAACTCGTTCTTTGTAATGACAAATTATCTCAAGTCAGAAGGCCAAGAACAGAA

GCTGTGTCCTGAGTATCCCAGCCGCGGTAAACAGTGCCATTCTGACCAGGGTTGTATAAAAGGATGGATGGACCC
ACAAAGTAAAGGAATCCAGACCGGCAGGTGTATACCTTACGACCAGAAGAGGAAGACCTGTGAAATCTTTGCCTG
GTGTCCTGCTGAGGAAGGGAAAGAAGCCCCACGGCCTGCACTCTTGAGGAGCGCCGAAAACTTCACCGTACTCAT
CAAGAACAATATCGACTTCCCGGGCCACAACTATACTACGAGAAACATCTTACCAGGTATGAACATCTCTTGTAC
CTTTCACAAGACTTGGAACCCTCAGTGTCCCATCTTCCGGCTAGGGGACATCTTCCAGGAAATCGGAGAGAACTT
TACAGAGGTGGCAGTTCAGGGAGGAATCATGGGCATTGAGATCTACTGGGACTGCAACCTGGACAGCTGGTCCCA
TCGCTGTCAACCCAAATACAGCTTCCGCCGGCTGGACGACAAGTACACCAATGAGTCCCTGTTCCCTGGCTACAA
CTTCAGATACGCCAAGTACTATAAGGAAAATGGCATGGAAAAGCGGACATTGATCAAAGCCTTCGGCGTGCGTTT
TGACATCCTGGTTTTTGGCACTGGAGGAAAGTTTGACATCATCCAGTTGGTTGTGTACATTGGATCCACCCTGTC
CTATTTCGGTTTGGCCACCGTGTGTATTGACTTGATCATCAACACGTATGCCAGTACCTGCTGCAGGTCACGTGT
TTACCCCTCCTGTAAGTGCTGCGAGCCCTGTGCAGTGAATGAGTACTACTACAGAAAGAAGTGTGAGCCCATCGT
GGAGCCCAAGCCGACGTTAAAGTATGTGTCCTTTGTGGACGAGCCCCACATTTGGATGGTGGACCAGCAGCTGCT
TGGGAAAAGTCTGCAAGATGTCAAAGGTCAAGAGGTCCCGAGACCCCAGACGGACTTCTTGGAACTGTCTAGGCT
CTCCCTCTCTCTCCACCACTCACCCCCAATTCCTGGACAACCTGAGGAAATGCAGCTGCTCCAGATAGAAGCGGT
TCCTAGGTCCAGGGACAGCCCAGATTGGTGCCAGTGTGGAAACTGCCTCCCGTCTCAACTACCAGAGAACCGCAG
GGCCCTGGAGGAGCTGTGCTGCCGGAGGAAGCCAGGACAGTGCATCACTACCTCTGAGCTCTTCAGTAAGATCGT
GCTATCCAGAGAGGCCCTGCAGCTCCTCCTGCTCTACCAGGAGCCCTTGCTGGCGCTGGAGGGAGAGGCCATCAA
CAGCAAGCTGCGACACTGTGCGTACAGGAGCTATGCCACCTGGCGCTTTGTCTCCCAAGACATGGCCGACTTTGC
CATTCTGCCCAGCTGCTGCCGCTGGAAGATCCGGAAGGAGTTCCCCAAGACCCAGGGGCAGTACTCTGGCTTCAA
GTATCCCTACTGACTATCTAGAGGATCCCCGGGTACGTACCGGGCCCCCCATCGAGGTCGACGTAGCTTTCATTT
GTTTT**AA**TTT**A**TTTTTTT**AAA**T**A**GC**A**TT**A**C**AAAA**C**AAA**TT**A**CC**A**C**AA**C**AAAAAAAAAA**CC**AAAA**C**AAAAA**C**AAA**T**A**
AT**AAA**GTCCC**AAAA**C**AAAAA**CGG**AA**T**A**TGC**AAA**C**AAAAAAAAAAAAAAAAAAAAAAAAAAAAAAAAAAAA**
AAAAAAAAAAGAATTGCTCGAGCGGCCGCTCGAGCAATTCCGGTCTCCCTATAGTGAGTCGTATTACTGGCGTAA
TAGCGAAGAGGCCCGCACCGATCGCCCTTCCCAACAGTTGCGTAGCCTGAATGGCGAATGGACGCGCCCTGTAG
CGGCGCATTAAGCGCGGCGGGTGTGGTGGTTACGCGCAGCGTGACCGCTACACTTGCCAGCGCCCTAGCGCCCGC
TCCTTTCGCTTTCTTCCCTTCCTTTCTCGCCACGTTCGCCGGCTTTCCCCGTCAAGCTCTAAATCGGGGGCTCCC
TTTAGGGTTCCGATTTAGTGCTTTACGGCACCTCGACCCCAAAAAACTTGATTAGGGTGATGGTTCACGTGGGCC
ATCGCCCTGATAGACGGTTTTTCGCCCTTTGACGTTGGAGTCCACGTTCTTTAATAGTGGACTCTTGTTCCAAAC
TGGAACAACACTCAACCCTATCTCGGTCTATTCTTTTGATTTATAAGGGATTTTGCCGATTTCGGCCTATTGGTT
AAAAAATGAGCTGATTTAACAAAATTTAACGCGAATTTTAACAAAATATTAACGTTTACAATTTCAGGTGGCACT
TTTCGGGGAAATGTGCGCGGAACCCCTATTTGTTTATTTTTCTAAATACATTCAAATATGTATCCGCTCATGAGA
CAATAACCCTGATAAATGCTTCAATAATATTGAAAAAGGAAGAGTATGAGTATTCAACATTTCCGTGTCGCCCTT
ATTCCCTTTTTTGCGGCATTTTGCCTTCCTGTTTTTGCTCACCCAGAAACGCTGGTGAAAGTAAAAGATGCTGAA
GATCAGTTGGGTGCACGAGTGGGTTACATCGAACTGGATCTCAACAGCGGTAAGATCCTTGAGAGTTTTCGCCCC
GAAGAACGTTTTCCAATGATGAGCACTTTTAAAGTTCTGCTATGTGGCGCGGTATTATCCCGTATTGACGCCGGG
CAAGAGCAACTCGGTCGCCGCATACACTATTCTCAGAATGACTTGGTTGAGTACTCACCAGTCACAGAAAAGCAT
CTTACGGATGGCATGACAGTAAGAGAATTATGCAGTGCTGCCATAACCATGAGTGATAACACTGCGGCCAACTTA
CTTCTGACAACGATCGGAGGACCGAAGGAGCTAACCGCTTTTTTGCACAACATGGGGGATCATGTAACTCGCCTT
GATCGTTGGGAACCGGAGCTGAATGAAGCCATACCAAACGACGAGCGTGACACCACGATGCCTGTAGCAATGGCA
ACAACGTTGCGCAAACTATTAACTGGCGAACTACTTACTCTAGCTTCCCGGCAACAATTAATAGACTGGATGGAG
GCGGATAAAGTTGCAGGACCACTTCTGCGCTCGGCCCTTCCGGCTGGCTGGTTTATTGCTGATAAATCTGGAGCC
GGTGAGCGTGGGTCTCGCGGTATCATTGCAGCACTGGGGCCAGATGGTAAGCCCTCCCGTATCGTAGTTATCTAC
ACGACGGGGAGTCAGGCAACTATGGATGAACGAAATAGACAGATCGCTGAGATAGGTGCCTCACTGATTAAGCAT
TGGTAACTGTCAGACCAAGTTTACTCATATATACTTTAGATTGATTTAAAACTTCATTTTTAATTTAAAAGGATC
TAGGTGAAGATCCTTTTTGATAATCTCATGACCAAAATCCCTTAACGTGAGTTTTCGTTCCACTGAGCGTCAGAC
CCCGTAGAAAAGATCAAAGGATCTTCTTGAGATCCTTTTTTTCTGCGCGTAATCTGCTGCTTGCAAACAAAAAAA
CCACCGCTACCAGCGGTGGTTTGTTTGCCGGATCAAGAGCTACCAACTCTTTTTCCGAAGGTAACTGGCTTCAGC
AGAGCGCAGATACCAAATACTGTCCTTCTAGTGTAGCCGTAGTTAGGCCACCACTTCAAGAACTCTGTAGCACCG
CCTACATACCTCGCTCTGCTAATCCTGTTACCAGTGGCTGCTGCCAGTGGCGATAAGTCGTGTCTTACCGGGTTG
GACTCAAGACGATAGTTACCGGATAAGGCGCAGCGGTCGGGCTGAACGGGGGGTTCGTGCACACAGCCCAGCTTG
GAGCGAACGACCTACACCGAACTGAGATACCTACAGCGTGAGCATTGAGAAAGCGCCACGCTTCCCGAAGGGAGA
AAGGCGGACAGGTATCCGGTAAGCGGCAGGGTCGGAACAGGAGAGCGCACGAGGGAGCTTCCAGGGGGAAACGCC
TGGTATCTTTATAGTCCTGTCGGGTTTCGCCACCTCTGACTTGAGCGTCGATTTTTGTGATGCTCGTCAGGGGGG
CGGAGCCTATGGAAAAACGCCAGCAACGCGGCCTTTTTACGGTTCCTGGCCTTTTGCTGGCCTTTTGCTCACATG
TTCTTTCCTGCGTTATCCCCTGATTCTGTGGATAACCGTATTACCGCCTTTGAGTGAGCTGATACCGCTCGCCGC
AGCCGAACGACCGAGCGCAGCGAGTCAGTGAGCGAGGAAGCGGAAGAGCGCCCAATACGCAAACCGCCTCTCCCC
GCGCGTTGGCCGATTCATTAATGCAGTAATACATAACCTTATGTATCATACACATACGATTTAGGTGACACTATA
G

>K-9245 ^{S3K64A}rP2X7^{S3}rP2X7-pNKS4 ($^{ko-wt}$dimer) 144.020 Da, pI 8.29, 1.269 residues

AATACAAGCTTACAACAAAGAACAACAAACAACAAAGTCCGACGTCGAAGTAGCCACCTACCATCATCATCATCA
TCCCATGGGAGCGCTTGGAGCCACCCGCAGTTCGAAAAAGGTGGAGGTTCTGGCGGTGGATCGGGAGGTTCAGC
GTGGAGCCACCCGCAGTTCGAGAAAGCTCCCGCTTGCTGTAGCTGGAACGATGTCTTTCAGTATGAGACAAACAA
AGTCACCCGGATCCAGAGCGTGAATTACGGCACCATCAAGTGGATCTTGCACATGACAGTCTTTTCCTACGTTAG
CTTTGCTTTGATGAGCGACAAGCTATATCAGCGGAAGGAGCCCCTTATCAGCTCTGTGCACACCGCTGTCAAAGG
CGTTGCAGAGGTGACAGAGAATGTCACGGAGGGCGGGGTGACGAAGTTAGTACACGGCATCTTCGACACGGCCGA
CTACACCCTCCCTTTGCAGGGGAACTCGTTCTTTGTAATGACAAATTATCTCAAGTCAGAAGGCCAAGAACAGAA
GCTGTGTCCTGAGTATCCCAGCCGCGGTAAACAGTGCCATTCTGACCAGGGTTGTATAAAAGGATGGATGGACCC
ACAAAGTAAAGGAATCCAGACCGGCAGGTGTATACCTTACGACCAGAAGAGGAAGACCTGTGAAATCTTTGCCTG
GTGTCCTGCTGAGGAAGGGAAAGAAGCCCCACGGCCTGCACTCTTGAGGAGCGCCGAAAACTTCACCGTACTCAT
CAAGAACAATATCGACTTCCCGGGCCACAACTATACTACGAGAAACATCTTACCAGGTATGAACATCTCTTGTAC
CTTTCACAAGACTTGGAACCCTCAGTGTCCCATCTTCCGGCTAGGGGACATCTTCCAGGAAATCGGAGAGAACTT
TACAGAGGTGGCAGTTCAGGGAGGAATCATGGGCATTGAGATCTACTGGGACTGCAACCTGGACAGCTGGTCCCA
TCGCTGTCAACCCAAATACAGCTTCCGCCGGCTGGACGACAAGTACACCAATGAGTCCCTGTTCCCTGGCTACAA
CTTCAGATACGCCAAGTACTATAAGGAAAATGGCATGGAAAAGCGGACATTGATCAAAGCCTTCGGCGTGCGTTT
TGACATCCTGGTTTTTGGCACTGGAGGAAAGTTTGACATCATCCAGTTGGTTGTGTACATTGGATCCACCCTGTC
CTATTTCGGTTTGGCCACCGTGTGTATTGACTTGATCATCAACACGTATGCCAGTACCTGCTGCAGGTCACGTGT
TTACCCCTCCTGTAAGTGCTGCGAGCCCTGTGCAGTGAATGAGTACTACTACAGAAAGAAGTGTGAGCCCATCGT
GGAGCCCAAGCCGACGTTAAAGTATGTGTCCTTTGTGGACGAGCCCCACATTTGGATGGTGGACCAGCAGCTGCT
TGGGAAAAGTCTGCAAGATGTCAAAGGTCAAGAGGTCCCGAGACCCCAGACGGACTTCTTGGAACTGTCTAGGCT
CTCCCTCTCTCTCCACCACTCACCCCCAATTCCTGGACAACCTGAGGAAATGCAGCTGCTCCAGATAGAAGCGGT
TCCTAGGTCCAGGGACAGCCCAGATTGGTGCCAGTGTGGAAACTGCCTCCCGTCTCAACTACCAGAGAACCGCAG
GGCCCTGGAGGAGCTGTGCTGCCGGAGGAAGCCAGGACAGTGCATCACTACCTCTGAGCTCTTCAGTAAGATCGT
GCTATCCAGAGAGGCCCTGCAGCTCCTCCTGCTCTACCAGGAGCCCTTGCTGGCGCTGGAGGGAGAGGCCATCAA
CAGCAAGCTGCGACACTGTGCGTACAGGAGCTATGCCACCTGGCGCTTTGTCTCCCAAGACATGGCCGACTTTGC
CATTCTGCCCAGCTGCTGCCGCTGGAAGATCCGGAAGGAGTTCCCCAAGACCCAGGGGCAGTACTCTGGCTTCAA
GTATCCCTACGGTGGATCTGGTGGCGGTGGATCAGGAGGCGGTGGATCAGGACTCATGGGAGCGCTTGGAGCCA
CCCGCAGTTCGAAAAAGGTGGAGGTTCTGGCGGTGGATCGGGAGGTTCAGCGTGGAGCCACCCGCAGTTCGAGAA
AGCTCCCGCTTGCTGTAGCTGGAACGATGTCTTTCAGTATGAGACAAACAAAGTCACCCGGATCCAGAGCGTGAA
TTACGGCACCATCAAGTGGATCTTGCACATGACAGTCTTTTCCTACGTTAGCTTTGCTTTGATGAGCGACAAGCT
ATATCAGCGGAAGGAGCCCCTTATCAGCTCTGTGCACACCAAGGTCAAAGGCGTTGCAGAGGTGACAGAGAATGT
CACGGAGGGCGGGGTGACGAAGTTAGTACACGGCATCTTCGACACGGCCGACTACACCCTCCCTTTGCAGGGGAA
CTCGTTCTTTGTAATGACAAATTATCTCAAGTCAGAAGGCCAAGAACAGAAGCTGTGTCCTGAGTATCCCAGCCG
CGGTAAACAGTGCCATTCTGACCAGGGTTGTATAAAAGGATGGATGGACCCACAAAGTAAAGGAATCCAGACCGG
CAGGTGTATACCTTACGACCAGAAGAGGAAGACCTGTGAAATCTTTGCCTGGTGTCCTGCTGAGGAAGGGAAAGA
AGCCCCACGGCCTGCACTCTTGAGGAGCGCCGAAAACTTCACCGTACTCATCAAGAACAATATCGACTTCCCGGG
CCACAACTATACTACGAGAAACATCTTACCAGGTATGAACATCTCTTGTACCTTTCACAAGACTTGGAACCCTCA
GTGTCCCATCTTCCGGCTAGGGGACATCTTCCAGGAAATCGGAGAGAACTTTACAGAGGTGGCAGTTCAGGGAGG
AATCATGGGCATTGAGATCTACTGGGACTGCAACCTGGACAGCTGGTCCCATCGCTGTCAACCCAAATACAGCTT
CCGCCGGCTGGACGACAAGTACACCAATGAGTCCCTGTTCCCTGGCTACAACTTCAGATACGCCAAGTACTATAA
GGAAAATGGCATGGAAAAGCGGACATTGATCAAAGCCTTCGGCGTGCGTTTTGACATCCTGGTTTTTGGCACTGG
AGGAAAGTTTGACATCATCCAGTTGGTTGTGTACATTGGATCCACCCTGTCCTATTTCGGTTTGGCCACCGTGTG
TATTGACTTGATCATCAACACGTATGCCAGTACCTGCTGCAGGTCACGTGTTTACCCCTCCTGTAAGTGCTGCGA
GCCCTGTGCAGTGAATGAGTACTACTACAGAAAGAAGTGTGAGCCCATCGTGGAGCCCAAGCCGACGTTAAAGTA
TGTGTCCTTTGTGGACGAGCCCCACATTTGGATGGTGGACCAGCAGCTGCTTGGGAAAAGTCTGCAAGATGTCAA
AGGTCAAGAGGTCCCGAGACCCCAGACGGACTTCTTGGAACTGTCTAGGCTCTCCCTCTCTCTCCACCACTCACC
CCCAATTCCTGGACAACCTGAGGAAATGCAGCTGCTCCAGATAGAAGCGGTTCCTAGGTCCAGGGACAGCCCAGA
TTGGTGCCAGTGTGGAAACTGCCTCCCGTCTCAACTACCAGAGAACCGCAGGGCCCTGGAGGAGCTGTGCTGCCG
GAGGAAGCCAGGACAGTGCATCACTACCTCTGAGCTCTTCAGTAAGATCGTGCTATCCAGAGAGGCCCTGCAGCT
CCTCCTGCTCTACCAGGAGCCCTTGCTGGCGCTGGAGGGAGAGGCCATCAACAGCAAGCTGCGACACTGTGCGTA
CAGGAGCTATGCCACCTGGCGCTTTGTCTCCCAAGACATGGCCGACTTTGCCATTCTGCCCAGCTGCTGCCGCTG
GAAGATCCGGAAGGAGTTCCCCAAGACCCAGGGGCAGTACTCTGGCTTCAAGTATCCCTACTGACTATCTAGAGG
ATCCCCGGGTACGTACCGGGCCCCCCATCGAGGTCGACGTAGCTTTCATTTGTTTT**AA**TTT**A**TTTTTT**AAA**T**A**GC
ATT**A**C**AAAA**C**AAA**TT**A**CC**A**C**AA**C**AAAAAAAAAA**CC**AAAA**C**AAAAA**C**AAA**T**AA**T**AAA**GTCCC**AAAA**C**AAAAA**CGG**A**

AT**A**TGC**AAA**C**AAAAAAAAAAAAAAAAAAAAAAAAAAAAAAAAAAAAAAAAAAAAAAAAAAAGAA**TTGCTCGAGCG
GCCGCTCGAGCAATTCCGGTCTCCCTATAGTGAGTCGTATTACTGGCGTAATAGCGAAGAGGCCCGCACCGATCG
CCCTTCCCAACAGTTGCGTAGCCTGAATGGCGAATGGACGCGCCCTGTAGCGGCGCATTAAGCGCGGCGGGTGT
GGTGGTTACGCGCAGCGTGACCGCTACACTTGCCAGCGCCCTAGCGCCCGCTCCTTTCGCTTTCTTCCCTTCCTT
TCTCGCCACGTTCGCCGGCTTTCCCCGTCAAGCTCTAAATCGGGGGCTCCCTTTAGGGTTCCGATTTAGTGCTTT
ACGGCACCTCGACCCCAAAAAACTTGATTAGGGTGATGGTTCACGTGGGCCATCGCCCTGATAGACGGTTTTTCG
CCCTTTGACGTTGGAGTCCACGTTCTTTAATAGTGGACTCTTGTTCCAAACTGGAACAACACTCAACCCTATCTC
GGTCTATTCTTTTGATTTATAAGGGATTTTGCCGATTTCGGCCTATTGGTTAAAAAATGAGCTGATTTAACAAAA
TTTAACGCGAATTTTAACAAAATATTAACGTTTACAATTTCAGGTGGCACTTTTCGGGGAAATGTGCGCGGAACC
CCTATTTGTTTATTTTTCTAAATACATTCAAATATGTATCCGCTCATGAGACAATAACCCTGATAAATGCTTCAA
TAATATTGAAAAAGGAAGAGTATGAGTATTCAACATTTCCGTGTCGCCCTTATTCCCTTTTTTGCGGCATTTTGC
CTTCCTGTTTTTGCTCACCCAGAAACGCTGGTGAAAGTAAAAGATGCTGAAGATCAGTTGGGTGCACGAGTGGGT
TACATCGAACTGGATCTCAACAGCGGTAAGATCCTTGAGAGTTTTCGCCCCGAAGAACGTTTTCCAATGATGAGC
ACTTTTAAAGTTCTGCTATGTGGCGCGGTATTATCCCGTATTGACGCCGGGCAAGAGCAACTCGGTCGCCGCATA
CACTATTCTCAGAATGACTTGGTTGAGTACTCACCAGTCACAGAAAAGCATCTTACGGATGGCATGACAGTAAGA
GAATTATGCAGTGCTGCCATAACCATGAGTGATAACACTGCGGCCAACTTACTTCTGACAACGATCGGAGGACCG
AAGGAGCTAACCGCTTTTTTGCACAACATGGGGGATCATGTAACTCGCCTTGATCGTTGGGAACCGGAGCTGAAT
GAAGCCATACCAAACGACGAGCGTGACACCACGATGCCTGTAGCAATGGCAACAACGTTGCGCAAACTATTAACT
GGCGAACTACTTACTCTAGCTTCCCGGCAACAATTAATAGACTGGATGGAGGCGGATAAAGTTGCAGGACCACTT
CTGCGCTCGGCCCTTCCGGCTGGCTGGTTTATTGCTGATAAATCTGGAGCCGGTGAGCGTGGGTCTCGCGGTATC
ATTGCAGCACTGGGGCCAGATGGTAAGCCCTCCCGTATCGTAGTTATCTACACGACGGGGAGTCAGGCAACTATG
GATGAACGAAATAGACAGATCGCTGAGATAGGTGCCTCACTGATTAAGCATTGGTAACTGTCAGACCAAGTTTAC
TCATATATACTTTAGATTGATTTAAAACTTCATTTTTAATTTAAAAGGATCTAGGTGAAGATCCTTTTTGATAAT
CTCATGACCAAAATCCCTTAACGTGAGTTTTCGTTCCACTGAGCGTCAGACCCCGTAGAAAAGATCAAAGGATCT
TCTTGAGATCCTTTTTTTCTGCGCGTAATCTGCTGCTTGCAAACAAAAAAACCACCGCTACCAGCGGTGGTTTGT
TTGCCGGATCAAGAGCTACCAACTCTTTTTCCGAAGGTAACTGGCTTCAGCAGAGCGCAGATACCAAATACTGTC
CTTCTAGTGTAGCCGTAGTTAGGCCACCACTTCAAGAACTCTGTAGCACCGCCTACATACCTCGCTCTGCTAATC
CTGTTACCAGTGGCTGCTGCCAGTGGCGATAAGTCGTGTCTTACCGGGTTGGACTCAAGACGATAGTTACCGGAT
AAGGCGCAGCGGTCGGGCTGAACGGGGGGTTCGTGCACACAGCCCAGCTTGGAGCGAACGACCTACACCGAACTG
AGATACCTACAGCGTGAGCATTGAGAAAGCGCCACGCTTCCCGAAGGGAGAAAGGCGGACAGGTATCCGGTAAGC
GGCAGGGTCGGAACAGGAGAGCGCACGAGGGAGCTTCCAGGGGGAAACGCCTGGTATCTTTATAGTCCTGTCGGG
TTTCGCCACCTCTGACTTGAGCGTCGATTTTTGTGATGCTCGTCAGGGGGGCGGAGCCTATGGAAAAACGCCAGC
AACGCGGCCTTTTTACGGTTCCTGGCCTTTTGCTGGCCTTTTGCTCACATGTTCTTTCCTGCGTTATCCCCTGAT
TCTGTGGATAACCGTATTACCGCCTTTGAGTGAGCTGATACCGCTCGCCGCAGCCGAACGACCGAGCGCAGCGAG
TCAGTGAGCGAGGAAGCGGAAGAGCGCCCAATACGCAAACCGCCTCTCCCCGCGCGTTGGCCGATTCATTAATGC
AGTAATACATAACCTTATGTATCATACACATACGATTTAGGTGACACTATAG

>K-9246 S3K64ArP2X7S3K64ArP2X7-pNKS4 (ko-kodimer) 144.020 Da, pI 8.29, 1.269 residues

AATACAAGCTTACAACAAAGAACAACAAACAACAAAGTCCGACGTCGAAGTAGCCACCTACCATCATCATCATCA
TCCCATGGGAGCGCTTGGAGCCACCCGCAGTTCGAAAAAGGTGGAGGTTCTGGCGGTGGATCGGGAGGTTCAGC
GTGGAGCCACCCGCAGTTCGAGAAAGCTCCCGCTTGCTGTAGCTGGAACGATGTCTTTCAGTATGAGACAAACAA
AGTCACCCGGATCCAGAGCGTGAATTACGGCACCATCAAGTGGATCTTGCACATGACAGTCTTTTCCTACGTTAG
CTTTGCTTTGATGAGCGACAAGCTATATCAGCGGAAGGAGCCCCTTATCAGCTCTGTGCACACCGCTGTCAAAGG
CGTTGCAGAGGTGACAGAGAATGTCACGGAGGGCGGGGTGACGAAGTTAGTACACGGCATCTTCGACACGGCCGA
CTACACCCTCCCTTTGCAGGGGAACTCGTTCTTTGTAATGACAAATTATCTCAAGTCAGAAGGCCAAGAACAGAA
GCTGTGTCCTGAGTATCCCAGCCGCGGTAAACAGTGCCATTCTGACCAGGGTTGTATAAAAGGATGGATGGACCC
ACAAAGTAAAGGAATCCAGACCGGCAGGTGTATACCTTACGACCAGAAGAGGAAGACCTGTGAAATCTTTGCCTG
GTGTCCTGCTGAGGAAGGGAAAGAAGCCCCACGGCCTGCACTCTTGAGGAGCGCCGAAAACTTCACCGTACTCAT
CAAGAACAATATCGACTTCCCGGGCCACAACTATACTACGAGAAACATCTTACCAGGTATGAACATCTCTTGTAC
CTTTCACAAGACTTGGAACCCTCAGTGTCCCATCTTCCGGCTAGGGGACATCTTCCAGGAAATCGGAGAGAACTT
TACAGAGGTGGCAGTTCAGGGAGGAATCATGGGCATTGAGATCTACTGGGACTGCAACCTGGACAGCTGGTCCCA
TCGCTGTCAACCCAAATACAGCTTCCGCCGGCTGGACGACAAGTACACCAATGAGTCCCTGTTCCCTGGCTACAA
CTTCAGATACGCCAAGTACTATAAGGAAAATGGCATGGAAAAGCGGACATTGATCAAAGCCTTCGGCGTGCGTTT
TGACATCCTGGTTTTTGGCACTGGAGGAAAGTTTGACATCATCCAGTTGGTTGTGTACATTGGATCCACCCTGTC
CTATTTCGGTTTGGCCACCGTGTGTATTGACTTGATCATCAACACGTATGCCAGTACCTGCTGCAGGTCACGTGT
TTACCCCTCCTGTAAGTGCTGCGAGCCCTGTGCAGTGAATGAGTACTACTACAGAAAGAAGTGTGAGCCCATCGT

GGAGCCCAAGCCGACGTTAAAGTATGTGTCCTTTGTGGACGAGCCCCACATTTGGATGGTGGACCAGCAGCTGCT
TGGGAAAAGTCTGCAAGATGTCAAAGGTCAAGAGGTCCCGAGACCCCAGACGGACTTCTTGGAACTGTCTAGGCT
CTCCCTCTCTCTCCACCACTCACCCCCAATTCCTGGACAACCTGAGGAAATGCAGCTGCTCCAGATAGAAGCGGT
TCCTAGGTCCAGGGACAGCCCAGATTGGTGCCAGTGTGGAAACTGCCTCCCGTCTCAACTACCAGAGAACCGCAG
GGCCCTGGAGGAGCTGTGCTGCCGGAGGAAGCCAGGACAGTGCATCACTACCTCTGAGCTCTTCAGTAAGATCGT
GCTATCCAGAGAGGCCCTGCAGCTCCTCCTGCTCTACCAGGAGCCCTTGCTGGCGCTGGAGGGAGAGGCCATCAA
CAGCAAGCTGCGACACTGTGCGTACAGGAGCTATGCCACCTGGCGCTTTGTCTCCCAAGACATGGCCGACTTTGC
CATTCTGCCCAGCTGCTGCCGCTGGAAGATCCGGAAGGAGTTCCCCAAGACCCAGGGGCAGTACTCTGGCTTCAA
GTATCCCTACGGTGGATCTGGTGGCGGTGGATCAGGAGGCGGTGGATCAGGACTCATGGGAGCGCTTGGAGCCA
CCCGCAGTTCGAAAAAGGTGGAGGTTCTGGCGGTGGATCGGGAGGTTCAGCGTGGAGCCACCCGCAGTTCGAGAA
AGCTCCCGCTTGCTGTAGCTGGAACGATGTCTTTCAGTATGAGACAAACAAAGTCACCCGGATCCAGAGCGTGAA
TTACGGCACCATCAAGTGGATCTTGCACATGACAGTCTTTTCCTACGTTAGCTTTGCTTTGATGAGCGACAAGCT
ATATCAGCGGAAGGAGCCCCTTATCAGCTCTGTGCACACCGCTGTCAAAGGCGTTGCAGAGGTGACAGAGAATGT
CACGGAGGGCGGGGTGACGAAGTTAGTACACGGCATCTTCGACACGGCCGACTACACCCTCCCTTTGCAGGGGAA
CTCGTTCTTTGTAATGACAAATTATCTCAAGTCAGAAGGCCAAGAACAGAAGCTGTGTCCTGAGTATCCCAGCCG
CGGTAAACAGTGCCATTCTGACCAGGGTTGTATAAAAGGATGGATGGACCCACAAAGTAAAGGAATCCAGACCGG
CAGGTGTATACCTTACGACCAGAAGAGGAAGACCTGTGAAATCTTTGCCTGGTGTCCTGCTGAGGAAGGGAAAGA
AGCCCCACGGCCTGCACTCTTGAGGAGCGCCGAAAACTTCACCGTACTCATCAAGAACAATATCGACTTCCCGGG
CCACAACTATACTACGAGAAACATCTTACCAGGTATGAACATCTCTTGTACCTTTCACAAGACTTGGAACCCTCA
GTGTCCCATCTTCCGGCTAGGGGACATCTTCCAGGAAATCGGAGAGAACTTTACAGAGGTGGCAGTTCAGGGAGG
AATCATGGGCATTGAGATCTACTGGGACTGCAACCTGGACAGCTGGTCCCATCGCTGTCAACCCAAATACAGCTT
CCGCCGGCTGGACGACAAGTACACCAATGAGTCCCTGTTCCCTGGCTACAACTTCAGATACGCCAAGTACTATAA
GGAAAATGGCATGGAAAAGCGGACATTGATCAAAGCCTTCGGCGTGCGTTTTGACATCCTGGTTTTTGGCACTGG
AGGAAAGTTTGACATCATCCAGTTGGTTGTGTACATTGGATCCACCCTGTCCTATTTCGGTTTGGCCACCGTGTG
TATTGACTTGATCATCAACACGTATGCCAGTACCTGCTGCAGGTCACGTGTTTACCCCTCCTGTAAGTGCTGCGA
GCCCTGTGCAGTGAATGAGTACTACTACAGAAAGAAGTGTGAGCCCATCGTGGAGCCCAAGCCGACGTTAAAGTA
TGTGTCCTTTGTGGACGAGCCCCACATTTGGATGGTGGACCAGCAGCTGCTTGGGAAAAGTCTGCAAGATGTCAA
AGGTCAAGAGGTCCCGAGACCCCAGACGGACTTCTTGGAACTGTCTAGGCTCTCCCTCTCTCTCCACCACTCACC
CCCAATTCCTGGACAACCTGAGGAAATGCAGCTGCTCCAGATAGAAGCGGTTCCTAGGTCCAGGGACAGCCCAGA
TTGGTGCCAGTGTGGAAACTGCCTCCCGTCTCAACTACCAGAGAACCGCAGGGCCCTGGAGGAGCTGTGCTGCCG
GAGGAAGCCAGGACAGTGCATCACTACCTCTGAGCTCTTCAGTAAGATCGTGCTATCCAGAGAGGCCCTGCAGCT
CCTCCTGCTCTACCAGGAGCCCTTGCTGGCGCTGGAGGGAGAGGCCATCAACAGCAAGCTGCGACACTGTGCGTA
CAGGAGCTATGCCACCTGGCGCTTTGTCTCCCAAGACATGGCCGACTTTGCCATTCTGCCCAGCTGCTGCCGCTG
GAAGATCCGGAAGGAGTTCCCCAAGACCCAGGGGCAGTACTCTGGCTTCAAGTATCCCTACTGACTATCTAGAGG
ATCCCCGGGTACGTACCGGGCCCCCCATCGAGGTCGACGTAGCTTTCATTTGTTTT**AA**TTT**A**TTTTTT**AAA**T**A**GC
ATT**A**C**AAAA**C**AAA**TT**A**CC**A**C**AA**C**AAAAAAAAAA**CC**AAAA**C**AAAAA**C**AAA**T**AA**T**AAA**GTCCC**AAAA**C**AAAAA**CGG**A**
AT**A**TGC**AAA**C**AAAAAAAAAAAAAAAAAAAAAAAAAAAAAAAAAAAAAAAAAAAAAAAAAAAGAA**TTGCTCGAGCG
GCCGCTCGAGCAATTCCGGTCTCCCTATAGTGAGTCGTATTACTGGCGTAATAGCGAAGAGGCCCGCACCGATCG
CCCTTCCCAACAGTTGCGTAGCCTGAATGGCGAATGGGACGCGCCCTGTAGCGGCGCATTAAGCGCGGCGGGTGT
GGTGGTTACGCGCAGCGTGACCGCTACACTTGCCAGCGCCCTAGCGCCCGCTCCTTTCGCTTTCTTCCCTTCCTT
TCTCGCCACGTTCGCCGGCTTTCCCCGTCAAGCTCTAAATCGGGGGCTCCCTTTAGGGTTCCGATTTAGTGCTTT
ACGGCACCTCGACCCCAAAAAACTTGATTAGGGTGATGGTTCACGTGGGCCATCGCCCTGATAGACGGTTTTTCG
CCCTTTGACGTTGGAGTCCACGTTCTTTAATAGTGGACTCTTGTTCCAAACTGGAACAACACTCAACCCTATCTC
GGTCTATTCTTTTGATTTATAAGGGATTTTGCCGATTTCGGCCTATTGGTTAAAAAATGAGCTGATTTAACAAAA
TTTAACGCGAATTTTAACAAAATATTAACGTTTACAATTTCAGGTGGCACTTTTCGGGGAAATGTGCGCGGAACC
CCTATTTGTTTATTTTTCTAAATACATTCAAATATGTATCCGCTCATGAGACAATAACCCTGATAAATGCTTCAA
TAATATTGAAAAAGGAAGAGTATGAGTATTCAACATTTCCGTGTCGCCCTTATTCCCTTTTTTGCGGCATTTTGC
CTTCCTGTTTTTGCTCACCCAGAAACGCTGGTGAAAGTAAAAGATGCTGAAGATCAGTTGGGTGCACGAGTGGGT
TACATCGAACTGGATCTCAACAGCGGTAAGATCCTTGAGAGTTTTCGCCCCGAAGAACGTTTTCCAATGATGAGC
ACTTTTAAAGTTCTGCTATGTGGCGCGGTATTATCCCGTATTGACGCCGGGCAAGAGCAACTCGGTCGCCGCATA
CACTATTCTCAGAATGACTTGGTTGAGTACTCACCAGTCACAGAAAAGCATCTTACGGATGGCATGACAGTAAGA
GAATTATGCAGTGCTGCCATAACCATGAGTGATAACACTGCGGCCAACTTACTTCTGACAACGATCGGAGGACCG
AAGGAGCTAACCGCTTTTTTGCACAACATGGGGGATCATGTAACTCGCCTTGATCGTTGGGAACCGGAGCTGAAT
GAAGCCATACCAAACGACGAGCGTGACACCACGATGCCTGTAGCAATGGCAACAACGTTGCGCAAACTATTAACT
GGCGAACTACTTACTCTAGCTTCCCGGCAACAATTAATAGACTGGATGGAGGCGGATAAAGTTGCAGGACCACTT
CTGCGCTCGGCCCTTCCGGCTGGCTGGTTTATTGCTGATAAATCTGGAGCCGGTGAGCGTGGGTCTCGCGGTATC
ATTGCAGCACTGGGGCCAGATGGTAAGCCCTCCCGTATCGTAGTTATCTACACGACGGGGAGTCAGGCAACTATG
GATGAACGAAATAGACAGATCGCTGAGATAGGTGCCTCACTGATTAAGCATTGGTAACTGTCAGACCAAGTTTAC

TCATATATACTTTAGATTGATTTAAAACTTCATTTTTAATTTAAAAGGATCTAGGTGAAGATCCTTTTTGATAAT
CTCATGACCAAAATCCCTTAACGTGAGTTTTCGTTCCACTGAGCGTCAGACCCCGTAGAAAAGATCAAAGGATCT
TCTTGAGATCCTTTTTTTCTGCGCGTAATCTGCTGCTTGCAAACAAAAAAACCACCGCTACCAGCGGTGGTTTGT
TTGCCGGATCAAGAGCTACCAACTCTTTTTCCGAAGGTAACTGGCTTCAGCAGAGCGCAGATACCAAATACTGTC
CTTCTAGTGTAGCCGTAGTTAGGCCACCACTTCAAGAACTCTGTAGCACCGCCTACATACCTCGCTCTGCTAATC
CTGTTACCAGTGGCTGCTGCCAGTGGCGATAAGTCGTGTCTTACCGGGTTGGACTCAAGACGATAGTTACCGGAT
AAGGCGCAGCGGTCGGGCTGAACGGGGGGTTCGTGCACACAGCCCAGCTTGGAGCGAACGACCTACACCGAACTG
AGATACCTACAGCGTGAGCATTGAGAAAGCGCCACGCTTCCCGAAGGGAGAAAGGCGGACAGGTATCCGGTAAGC
GGCAGGGTCGGAACAGGAGAGCGCACGAGGGAGCTTCCAGGGGGAAACGCCTGGTATCTTTATAGTCCTGTCGGG
TTTCGCCACCTCTGACTTGAGCGTCGATTTTTGTGATGCTCGTCAGGGGGGCGGAGCCTATGGAAAAACGCCAGC
AACGCGGCCTTTTTACGGTTCCTGGCCTTTTGCTGGCCTTTTGCTCACATGTTCTTTCCTGCGTTATCCCCTGAT
TCTGTGGATAACCGTATTACCGCCTTTGAGTGAGCTGATACCGCTCGCCGCAGCCGAACGACCGAGCGCAGCGAG
TCAGTGAGCGAGGAAGCGGAAGAGCGCCCAATACGCAAACCGCCTCTCCCCGCGCGTTGGCCGATTCATTAATGC
AGTAATACATAACCTTATGTATCATACACATACGATTTAGGTGACACTATAG

>K-9247 ^{S3}**rP2X7**^{S3K64A}**rP2X7**-pNKS4 ($^{wt-ko}$dimer) 144.021 Da, pI 8.29, 1.269 residues

AATACAAGCTTACAACAAAGAACAACAAACAACAAAGTCCGACGTCGAAGTAGCCACCTACCATCATCATCATCA
TCCCATGGGGAGCGCTTGGAGCCACCCGCAGTTCGAAAAAGGTGGAGGTTCTGGCGGTGGATCGGGAGGTTCAGC
GTGGAGCCACCCGCAGTTCGAGAAAGCTCCCGCTTGCTGTAGCTGGAACGATGTCTTTCAGTATGAGACAAACAA
AGTCACCCGGATCCAGAGCGTGAATTACGGCACCATCAAGTGGATCTTGCACATGACAGTCTTTTCCTACGTTAG
CTTTGCTTTGATGAGCGACAAGCTATATCAGCGGAAGGAGCCCCTTATCAGCTCTGTGCACACCAAGGTCAAAGG
CGTTGCAGAGGTGACAGAGAATGTCACGGAGGGCGGGGTGACGAAGTTAGTACACGGCATCTTCGACACGGCCGA
CTACACCCTCCCTTTGCAGGGGAACTCGTTCTTTGTAATGACAAATTATCTCAAGTCAGAAGGCCAAGAACAGAA
GCTGTGTCCTGAGTATCCCAGCCGCGGTAAACAGTGCCATTCTGACCAGGGTTGTATAAAAGGATGGATGGACCC
ACAAAGTAAAGGAATCCAGACCGGCAGGTGTATACCTTACGACCAGAAGAGGAAGACCTGTGAAATCTTTGCCTG
GTGTCCTGCTGAGGAAGGGAAAGAAGCCCCACGGCCTGCACTCTTGAGGAGCGCCGAAAACTTCACCGTACTCAT
CAAGAACAATATCGACTTCCCGGGCCACAACTATACTACGAGAAACATCTTACCAGGTATGAACATCTCTTGTAC
CTTTCACAAGACTTGGAACCCTCAGTGTCCCATCTTCCGGCTAGGGGACATCTTCCAGGAAATCGGAGAGAACTT
TACAGAGGTGGCAGTTCAGGGAGGAATCATGGGCATTGAGATCTACTGGGACTGCAACCTGGACAGCTGGTCCCA
TCGCTGTCAACCCAAATACAGCTTCCGCCGGCTGGACGACAAGTACACCAATGAGTCCCTGTTCCCTGGCTACAA
CTTCAGATACGCCAAGTACTATAAGGAAAATGGCATGGAAAAGCGGACATTGATCAAAGCCTTCGGCGTGCGTTT
TGACATCCTGGTTTTTGGCACTGGAGGAAAGTTTGACATCATCCAGTTGGTTGTGTACATTGGATCCACCCTGTC
CTATTTCGGTTTGGCCACCGTGTGTATTGACTTGATCATCAACACGTATGCCAGTACCTGCTGCAGGTCACGTGT
TTACCCCTCCTGTAAGTGCTGCGAGCCCTGTGCAGTGAATGAGTACTACTACAGAAAGAAGTGTGAGCCCATCGT
GGAGCCCAAGCCGACGTTAAAGTATGTGTCCTTTGTGGACGAGCCCCACATTTGGATGGTGGACCAGCAGCTGCT
TGGGAAAAGTCTGCAAGATGTCAAAGGTCAAGAGGTCCCGAGACCCCAGACGGACTTCTTGGAACTGTCTAGGCT
CTCCCTCTCTCTCCACCACTCACCCCCAATTCCTGGACAACCTGAGGAAATGCAGCTGCTCCAGATAGAAGCGGT
TCCTAGGTCCAGGGACAGCCCAGATTGGTGCCAGTGTGGAAACTGCCTCCCGTCTCAACTACCAGAGAACCGCAG
GGCCCTGGAGGAGCTGTGCTGCCGGAGGAAGCCAGGACAGTGCATCACTACCTCTGAGCTCTTCAGTAAGATCGT
GCTATCCAGAGAGGCCCTGCAGCTCCTCCTGCTCTACCAGGAGCCCTTGCTGGCGCTGGAGGGAGAGGCCATCAA
CAGCAAGCTGCGACACTGTGCGTACAGGAGCTATGCCACCTGGCGCTTTGTCTCCCAAGACATGGCCGACTTTGC
CATTCTGCCCAGCTGCTGCCGCTGGAAGATCCGGAAGGAGTTCCCCAAGACCCAGGGGCAGTACTCTGGCTTCAA
GTATCCCTACGGTGGATCTGGTGGCGGTGGATCAGGAGGCGGTGGATCAGGACTCATGGGGAGCGCTTGGAGCCA
CCCGCAGTTCGAAAAAGGTGGAGGTTCTGGCGGTGGATCGGGAGGTTCAGCGTGGAGCCACCCGCAGTTCGAGAA
AGCTCCCGCTTGCTGTAGCTGGAACGATGTCTTTCAGTATGAGACAAACAAAGTCACCCGGATCCAGAGCGTGAA
TTACGGCACCATCAAGTGGATCTTGCACATGACAGTCTTTTCCTACGTTAGCTTTGCTTTGATGAGCGACAAGCT
ATATCAGCGGAAGGAGCCCCTTATCAGCTCTGTGCACACCGCTGTCAAAGGCGTTGCAGAGGTGACAGAGAATGT
CACGGAGGGCGGGGTGACGAAGTTAGTACACGGCATCTTCGACACGGCCGACTACACCCTCCCTTTGCAGGGGAA
CTCGTTCTTTGTAATGACAAATTATCTCAAGTCAGAAGGCCAAGAACAGAAGCTGTGTCCTGAGTATCCCAGCCG
CGGTAAACAGTGCCATTCTGACCAGGGTTGTATAAAAGGATGGATGGACCCACAAAGTAAAGGAATCCAGACCGG
CAGGTGTATACCTTACGACCAGAAGAGGAAGACCTGTGAAATCTTTGCCTGGTGTCCTGCTGAGGAAGGGAAAGA
AGCCCCACGGCCTGCACTCTTGAGGAGCGCCGAAAACTTCACCGTACTCATCAAGAACAATATCGACTTCCCGGG
CCACAACTATACTACGAGAAACATCTTACCAGGTATGAACATCTCTTGTACCTTTCACAAGACTTGGAACCCTCA
GTGTCCCATCTTCCGGCTAGGGGACATCTTCCAGGAAATCGGAGAGAACTTTACAGAGGTGGCAGTTCAGGGAGG
AATCATGGGCATTGAGATCTACTGGGACTGCAACCTGGACAGCTGGTCCCATCGCTGTCAACCCAAATACAGCTT
CCGCCGGCTGGACGACAAGTACACCAATGAGTCCCTGTTCCCTGGCTACAACTTCAGATACGCCAAGTACTATAA

GGAAAATGGCATGGAAAAGCGGACATTGATCAAAGCCTTCGGCGTGCGTTTTGACATCCTGGTTTTTGGCACTGG
AGGAAAGTTTGACATCATCCAGTTGGTTGTGTACATTGGATCCACCCTGTCCTATTTCGGTTTGGCCACCGTGTG
TATTGACTTGATCATCAACACGTATGCCAGTACCTGCTGCAGGTCACGTGTTTACCCCTCCTGTAAGTGCTGCGA
GCCCTGTGCAGTGAATGAGTACTACTACAGAAAGAAGTGTGAGCCCATCGTGGAGCCCAAGCCGACGTTAAAGTA
TGTGTCCTTTGTGGACGAGCCCCACATTTGGATGGTGGACCAGCAGCTGCTTGGGAAAAGTCTGCAAGATGTCAA
AGGTCAAGAGGTCCCGAGACCCCAGACGGACTTCTTGGAACTGTCTAGGCTCTCCCTCTCTCTCCACCACTCACC
CCCAATTCCTGGACAACCTGAGGAAATGCAGCTGCTCCAGATAGAAGCGGTTCCTAGGTCCAGGGACAGCCCAGA
TTGGTGCCAGTGTGGAAACTGCCTCCCGTCTCAACTACCAGAGAACCGCAGGGCCCTGGAGGAGCTGTGCTGCCG
GAGGAAGCCAGGACAGTGCATCACTACCTCTGAGCTCTTCAGTAAGATCGTGCTATCCAGAGAGGCCCTGCAGCT
CCTCCTGCTCTACCAGGAGCCCTTGCTGGCGCTGGAGGGAGAGGCCATCAACAGCAAGCTGCGACACTGTGCGTA
CAGGAGCTATGCCACCTGGCGCTTTGTCTCCCAAGACATGGCCGACTTTGCCATTCTGCCCAGCTGCTGCCGCTG
GAAGATCCGGAAGGAGTTCCCCAAGACCCAGGGGCAGTACTCTGGCTTCAAGTATCCCTACTGACTATCTAGAGG
ATCCCCGGGTACGTACCGGGCCCCCCATCGAGGTCGACGTAGCTTTCATTTGTTTT**AA**TTT**A**TTTTTT**AAA**T**A**GC
ATT**A**C**AAAA**C**AAA**TT**A**CC**A**C**AA**C**AAAAAAAAAA**CC**AAAA**C**AAAAA**C**AAA**T**AA**T**AAA**GTCCC**AAAA**C**AAAAA**CGG**A**
AT**A**TGC**AAA**C**AAAAAAAAAAAAAAAAAAAAAAAAAAAAAAAAAAAAAAAAAAAAAAAAAAAGAA**TTGCTCGAGCG
GCCGCTCGAGCAATTCCGGTCTCCCTATAGTGAGTCGTATTACTGGCGTAATAGCGAAGAGGCCCGCACCGATCG
CCCTTCCCAACAGTTGCGTAGCCTGAATGGCGAATGGGACGCGCCCTGTAGCGGCGCATTAAGCGCGGCGGGTGT
GGTGGTTACGCGCAGCGTGACCGCTACACTTGCCAGCGCCCTAGCGCCCGCTCCTTTCGCTTTCTTCCCTTCCTT
TCTCGCCACGTTCGCCGGCTTTCCCCGTCAAGCTCTAAATCGGGGGCTCCCTTTAGGGTTCCGATTTAGTGCTTT
ACGGCACCTCGACCCCAAAAAACTTGATTAGGGTGATGGTTCACGTGGGCCATCGCCCTGATAGACGGTTTTTCG
CCCTTTGACGTTGGAGTCCACGTTCTTTAATAGTGGACTCTTGTTCCAAACTGGAACAACACTCAACCCTATCTC
GGTCTATTCTTTTGATTTATAAGGGATTTTGCCGATTTCGGCCTATTGGTTAAAAAATGAGCTGATTTAACAAAA
TTTAACGCGAATTTTAACAAAATATTAACGTTTACAATTTCAGGTGGCACTTTTCGGGGAAATGTGCGCGGAACC
CCTATTTGTTTATTTTTCTAAATACATTCAAATATGTATCCGCTCATGAGACAATAACCCTGATAAATGCTTCAA
TAATATTGAAAAAGGAAGAGTATGAGTATTCAACATTTCCGTGTCGCCCTTATTCCCTTTTTTGCGGCATTTTGC
CTTCCTGTTTTTGCTCACCCAGAAACGCTGGTGAAAGTAAAAGATGCTGAAGATCAGTTGGGTGCACGAGTGGGT
TACATCGAACTGGATCTCAACAGCGGTAAGATCCTTGAGAGTTTTCGCCCCGAAGAACGTTTTCCAATGATGAGC
ACTTTTAAAGTTCTGCTATGTGGCGCGGTATTATCCCGTATTGACGCCGGGCAAGAGCAACTCGGTCGCCGCATA
CACTATTCTCAGAATGACTTGGTTGAGTACTCACCAGTCACAGAAAAGCATCTTACGGATGGCATGACAGTAAGA
GAATTATGCAGTGCTGCCATAACCATGAGTGATAACACTGCGGCCAACTTACTTCTGACAACGATCGGAGGACCG
AAGGAGCTAACCGCTTTTTTGCACAACATGGGGGATCATGTAACTCGCCTTGATCGTTGGGAACCGGAGCTGAAT
GAAGCCATACCAAACGACGAGCGTGACACCACGATGCCTGTAGCAATGGCAACAACGTTGCGCAAACTATTAACT
GGCGAACTACTTACTCTAGCTTCCCGGCAACAATTAATAGACTGGATGGAGGCGGATAAAGTTGCAGGACCACTT
CTGCGCTCGGCCCTTCCGGCTGGCTGGTTTATTGCTGATAAATCTGGAGCCGGTGAGCGTGGGTCTCGCGGTATC
ATTGCAGCACTGGGGCCAGATGGTAAGCCCTCCCGTATCGTAGTTATCTACACGACGGGGAGTCAGGCAACTATG
GATGAACGAAATAGACAGATCGCTGAGATAGGTGCCTCACTGATTAAGCATTGGTAACTGTCAGACCAAGTTTAC
TCATATATACTTTAGATTGATTTAAAACTTCATTTTTAATTTAAAAGGATCTAGGTGAAGATCCTTTTTGATAAT
CTCATGACCAAAATCCCTTAACGTGAGTTTTCGTTCCACTGAGCGTCAGACCCCGTAGAAAAGATCAAAGGATCT
TCTTGAGATCCTTTTTTTCTGCGCGTAATCTGCTGCTTGCAAACAAAAAAACCACCGCTACCAGCGGTGGTTTGT
TTGCCGGATCAAGAGCTACCAACTCTTTTTCCGAAGGTAACTGGCTTCAGCAGAGCGCAGATACCAAATACTGTC
CTTCTAGTGTAGCCGTAGTTAGGCCACCACTTCAAGAACTCTGTAGCACCGCCTACATACCTCGCTCTGCTAATC
CTGTTACCAGTGGCTGCTGCCAGTGGCGATAAGTCGTGTCTTACCGGGTTGGACTCAAGACGATAGTTACCGGAT
AAGGCGCAGCGGTCGGGCTGAACGGGGGGTTCGTGCACACAGCCCAGCTTGGAGCGAACGACCTACACCGAACTG
AGATACCTACAGCGTGAGCATTGAGAAAGCGCCACGCTTCCCGAAGGGAGAAAGGCGGACAGGTATCCGGTAAGC
GGCAGGGTCGGAACAGGAGAGCGCACGAGGGAGCTTCCAGGGGGAAACGCCTGGTATCTTTATAGTCCTGTCGGG
TTTCGCCACCTCTGACTTGAGCGTCGATTTTTGTGATGCTCGTCAGGGGGGCGGAGCCTATGGAAAAACGCCAGC
AACGCGGCCTTTTTACGGTTCCTGGCCTTTTGCTGGCCTTTTGCTCACATGTTCTTTCCTGCGTTATCCCCTGAT
TCTGTGGATAACCGTATTACCGCCTTTGAGTGAGCTGATACCGCTCGCCGCAGCCGAACGACCGAGCGCAGCGAG
TCAGTGAGCGAGGAAGCGGAAGAGCGCCCAATACGCAAACCGCCTCTCCCCGCGCGTTGGCCGATTCATTAATGC
AGTAATACATAACCTTATGTATCATACACATACGATTTAGGTGACACTATAG

>K-9250 ^{S3}rP2X7^{S3K64A}rP2X7^{S3}rP2X7 ($^{wt-ko-wt}$trimer) 216.551 Da, pI 8.29, 1.911 residues

AATACAAGCTTACAACAAAGAACAACAAACAACAAAGTCCGACGTCGAAGTAGCCACCTACCATCATCATCATCA
TCCCATGGGGAGCGCTTGGAGCCACCCGCAGTTCGAAAAAGGTGGAGGTTCTGGCGGTGGATCGGGAGGTTCAGC
GTGGAGCCACCCGCAGTTCGAGAAAGCTCCCGCTTGCTGTAGCTGGAACGATGTCTTTCAGTATGAGACAAACAA
AGTCACCCGGATCCAGAGCGTGAATTACGGCACCATCAAGTGGATCTTGCACATGACAGTCTTTTCCTACGTTAG

CTTTGCTTTGATGAGCGACAAGCTATATCAGCGGAAGGAGCCCCTTATCAGCTCTGTGCACACCAAGGTCAAAGG
CGTTGCAGAGGTGACAGAGAATGTCACGGAGGGCGGGGTGACGAAGTTAGTACACGGCATCTTCGACACGGCCGA
CTACACCCTCCCTTTGCAGGGGAACTCGTTCTTTGTAATGACAAATTATCTCAAGTCAGAAGGCCAAGAACAGAA
GCTGTGTCCTGAGTATCCCAGCCGCGGTAAACAGTGCCATTCTGACCAGGGTTGTATAAAAGGATGGATGGACCC
ACAAAGTAAAGGAATCCAGACCGGCAGGTGTATACCTTACGACCAGAAGAGGAAGACCTGTGAAATCTTTGCCTG
GTGTCCTGCTGAGGAAGGGAAAGAAGCCCCACGGCCTGCACTCTTGAGGAGCGCCGAAAACTTCACCGTACTCAT
CAAGAACAATATCGACTTCCCGGGCCACAACTATACTACGAGAAACATCTTACCAGGTATGAACATCTCTTGTAC
CTTTCACAAGACTTGGAACCCTCAGTGTCCCATCTTCCGGCTAGGGGACATCTTCCAGGAAATCGGAGAGAACTT
TACAGAGGTGGCAGTTCAGGGAGGAATCATGGGCATTGAGATCTACTGGGACTGCAACCTGGACAGCTGGTCCCA
TCGCTGTCAACCCAAATACAGCTTCCGCCGGCTGGACGACAAGTACACCAATGAGTCCCTGTTCCCTGGCTACAA
CTTCAGATACGCCAAGTACTATAAGGAAAATGGCATGGAAAAGCGGACATTGATCAAAGCCTTCGGCGTGCGTTT
TGACATCCTGGTTTTTGGCACTGGAGGAAAGTTTGACATCATCCAGTTGGTTGTGTACATTGGATCCACCCTGTC
CTATTTCGGTTTGGCCACCGTGTGTATTGACTTGATCATCAACACGTATGCCAGTACCTGCTGCAGGTCACGTGT
TTACCCCTCCTGTAAGTGCTGCGAGCCCTGTGCAGTGAATGAGTACTACTACAGAAAGAAGTGTGAGCCCATCGT
GGAGCCCAAGCCGACGTTAAAGTATGTGTCCTTTGTGGACGAGCCCCACATTTGGATGGTGGACCAGCAGCTGCT
TGGGAAAAGTCTGCAAGATGTCAAAGGTCAAGAGGTCCCGAGACCCCAGACGGACTTCTTGGAACTGTCTAGGCT
CTCCCTCTCTCTCCACCACTCACCCCCAATTCCTGGACAACCTGAGGAAATGCAGCTGCTCCAGATAGAAGCGGT
TCCTAGGTCCAGGGACAGCCCAGATTGGTGCCAGTGTGGAAACTGCCTCCCGTCTCAACTACCAGAGAACCGCAG
GGCCCTGGAGGAGCTGTGCTGCCGGAGGAAGCCAGGACAGTGCATCACTACCTCTGAGCTCTTCAGTAAGATCGT
GCTATCCAGAGAGGCCCTGCAGCTCCTCCTGCTCTACCAGGAGCCCTTGCTGGCGCTGGAGGGAGAGGCCATCAA
CAGCAAGCTGCGACACTGTGCGTACAGGAGCTATGCCACCTGGCGCTTTGTCTCCCAAGACATGGCCGACTTTGC
CATTCTGCCCAGCTGCTGCCGCTGGAAGATCCGGAAGGAGTTCCCCAAGACCCAGGGGCAGTACTCTGGCTTCAA
GTATCCCTACGGTGGATCTGGTGGCGGTGGATCAGGAGGCGGTGGATCAGGACTCATGGGGAGCGCTTGGAGCCA
CCCGCAGTTCGAAAAAGGTGGAGGTTCTGGCGGTGGATCGGGAGGTTCAGCGTGGAGCCACCCGCAGTTCGAGAA
AGCTCCCGCTTGCTGTAGCTGGAACGATGTCTTTCAGTATGAGACAAACAAAGTCACCCGGATCCAGAGCGTGAA
TTACGGCACCATCAAGTGGATCTTGCACATGACAGTCTTTTCCTACGTTAGCTTTGCTTTGATGAGCGACAAGCT
ATATCAGCGGAAGGAGCCCCTTATCAGCTCTGTGCACACCGCTGTCAAAGGCGTTGCAGAGGTGACAGAGAATGT
CACGGAGGGCGGGGTGACGAAGTTAGTACACGGCATCTTCGACACGGCCGACTACACCCTCCCTTTGCAGGGGAA
CTCGTTCTTTGTAATGACAAATTATCTCAAGTCAGAAGGCCAAGAACAGAAGCTGTGTCCTGAGTATCCCAGCCG
CGGTAAACAGTGCCATTCTGACCAGGGTTGTATAAAAGGATGGATGGACCCACAAAGTAAAGGAATCCAGACCGG
CAGGTGTATACCTTACGACCAGAAGAGGAAGACCTGTGAAATCTTTGCCTGGTGTCCTGCTGAGGAAGGGAAAGA
AGCCCCACGGCCTGCACTCTTGAGGAGCGCCGAAAACTTCACCGTACTCATCAAGAACAATATCGACTTCCCGGG
CCACAACTATACTACGAGAAACATCTTACCAGGTATGAACATCTCTTGTACCTTTCACAAGACTTGGAACCCTCA
GTGTCCCATCTTCCGGCTAGGGGACATCTTCCAGGAAATCGGAGAGAACTTTACAGAGGTGGCAGTTCAGGGAGG
AATCATGGGCATTGAGATCTACTGGGACTGCAACCTGGACAGCTGGTCCCATCGCTGTCAACCCAAATACAGCTT
CCGCCGGCTGGACGACAAGTACACCAATGAGTCCCTGTTCCCTGGCTACAACTTCAGATACGCCAAGTACTATAA
GGAAAATGGCATGGAAAAGCGGACATTGATCAAAGCCTTCGGCGTGCGTTTTGACATCCTGGTTTTTGGCACTGG
AGGAAAGTTTGACATCATCCAGTTGGTTGTGTACATTGGATCCACCCTGTCCTATTTCGGTTTGGCCACCGTGTG
TATTGACTTGATCATCAACACGTATGCCAGTACCTGCTGCAGGTCACGTGTTTACCCCTCCTGTAAGTGCTGCGA
GCCCTGTGCAGTGAATGAGTACTACTACAGAAAGAAGTGTGAGCCCATCGTGGAGCCCAAGCCGACGTTAAAGTA
TGTGTCCTTTGTGGACGAGCCCCACATTTGGATGGTGGACCAGCAGCTGCTTGGGAAAAGTCTGCAAGATGTCAA
AGGTCAAGAGGTCCCGAGACCCCAGACGGACTTCTTGGAACTGTCTAGGCTCTCCCTCTCTCTCCACCACTCACC
CCCAATTCCTGGACAACCTGAGGAAATGCAGCTGCTCCAGATAGAAGCGGTTCCTAGGTCCAGGGACAGCCCAGA
TTGGTGCCAGTGTGGAAACTGCCTCCCGTCTCAACTACCAGAGAACCGCAGGGCCCTGGAGGAGCTGTGCTGCCG
GAGGAAGCCAGGACAGTGCATCACTACCTCTGAGCTCTTCAGTAAGATCGTGCTATCCAGAGAGGCCCTGCAGCT
CCTCCTGCTCTACCAGGAGCCCTTGCTGGCGCTGGAGGGAGAGGCCATCAACAGCAAGCTGCGACACTGTGCGTA
CAGGAGCTATGCCACCTGGCGCTTTGTCTCCCAAGACATGGCCGACTTTGCCATTCTGCCCAGCTGCTGCCGCTG
GAAGATCCGGAAGGAGTTCCCCAAGACCCAGGGGCAGTACTCTGGCTTCAAGTATCCCTACGGTGGATCTGGTGG
CGGTGGATCAGGAGGCGGTGGATCAGGACTCATGGGGAGCGCTTGGAGCCACCCGCAGTTCGAAAAAGGTGGAGG
TTCTGGCGGTGGATCGGGAGGTTCAGCGTGGAGCCACCCGCAGTTCGAGAAAGCTCCCGCTTGCTGTAGCTGGAA
CGATGTCTTTCAGTATGAGACAAACAAAGTCACCCGGATCCAGAGCGTGAATTACGGCACCATCAAGTGGATCTT
GCACATGACAGTCTTTTCCTACGTTAGCTTTGCTTTGATGAGCGACAAGCTATATCAGCGGAAGGAGCCCCTTAT
CAGCTCTGTGCACACCAAGGTCAAAGGCGTTGCAGAGGTGACAGAGAATGTCACGGAGGGCGGGGTGACGAAGTT
AGTACACGGCATCTTCGACACGGCCGACTACACCCTCCCTTTGCAGGGGAACTCGTTCTTTGTAATGACAAATTA
TCTCAAGTCAGAAGGCCAAGAACAGAAGCTGTGTCCTGAGTATCCCAGCCGCGGTAAACAGTGCCATTCTGACCA
GGGTTGTATAAAAGGATGGATGGACCCACAAAGTAAAGGAATCCAGACCGGCAGGTGTATACCTTACGACCAGAA
GAGGAAGACCTGTGAAATCTTTGCCTGGTGTCCTGCTGAGGAAGGGAAAGAAGCCCCACGGCCTGCACTCTTGAG
GAGCGCCGAAAACTTCACCGTACTCATCAAGAACAATATCGACTTCCCGGGCCACAACTATACTACGAGAAACAT

CTTACCAGGTATGAACATCTCTTGTACCTTTCACAAGACTTGGAACCCTCAGTGTCCCATCTTCCGGCTAGGGGA
CATCTTCCAGGAAATCGGAGAGAACTTTACAGAGGTGGCAGTTCAGGGAGGAATCATGGGCATTGAGATCTACTG
GGACTGCAACCTGGACAGCTGGTCCCATCGCTGTCAACCCAAATACAGCTTCCGCCGGCTGGACGACAAGTACAC
CAATGAGTCCCTGTTCCCTGGCTACAACTTCAGATACGCCAAGTACTATAAGGAAAATGGCATGGAAAAGCGGAC
ATTGATCAAAGCCTTCGGCGTGCGTTTTGACATCCTGGTTTTTGGCACTGGAGGAAAGTTTGACATCATCCAGTT
GGTTGTGTACATTGGATCCACCCTGTCCTATTTCGGTTTGGCCACCGTGTGTATTGACTTGATCATCAACACGTA
TGCCAGTACCTGCTGCAGGTCACGTGTTTACCCCTCCTGTAAGTGCTGCGAGCCCTGTGCAGTGAATGAGTACTA
CTACAGAAAGAAGTGTGAGCCCATCGTGGAGCCCAAGCCGACGTTAAAGTATGTGTCCTTTGTGGACGAGCCCCA
CATTTGGATGGTGGACCAGCAGCTGCTTGGGAAAAGTCTGCAAGATGTCAAAGGTCAAGAGGTCCCGAGACCCCA
GACGGACTTCTTGGAACTGTCTAGGCTCTCCCTCTCTCTCCACCACTCACCCCCAATTCCTGGACAACCTGAGGA
AATGCAGCTGCTCCAGATAGAAGCGGTTCCTAGGTCCAGGGACAGCCCAGATTGGTGCCAGTGTGGAAACTGCCT
CCCGTCTCAACTACCAGAGAACCGCAGGGCCCTGGAGGAGCTGTGCTGCCGGAGGAAGCCAGGACAGTGCATCAC
TACCTCTGAGCTCTTCAGTAAGATCGTGCTATCCAGAGAGGCCCTGCAGCTCCTCCTGCTCTACCAGGAGCCCTT
GCTGGCGCTGGAGGGAGAGGCCATCAACAGCAAGCTGCGACACTGTGCGTACAGGAGCTATGCCACCTGGCGCTT
TGTCTCCCAAGACATGGCCGACTTTGCCATTCTGCCCAGCTGCTGCCGCTGGAAGATCCGGAAGGAGTTCCCCAA
GACCCAGGGGCAGTACTCTGGCTTCAAGTATCCCTACTGACTATCTAGAGGATCCCCGGGTACGTACCGGGCCCC
CCATCGAGGTCGACGTAGCTTTCATTTGTTTT**AA**TTT**A**TTTTTT**AAA**T**A**GC**A**TT**A**C**AAAA**C**AAA**TT**A**CC**A**C**AA**C**A**
AAAAAAAAACC**AAAA**C**AAAAA**C**AAA**T**AA**T**AAA**GTCCC**AAAA**C**AAAAA**CGG**AA**T**A**TGC**AAA**C**AAAAAAAAAAAAAA**
AAAAAAAAAAAAAAAAAAAAAAAAAAAAAAAAAAAAAGAATTGCTCGAGCGGCCGCTCGAGCAATTCCGGTCTCC
CTATAGTGAGTCGTATTACTGGCGTAATAGCGAAGAGGCCCGCACCGATCGCCCTTCCCAACAGTTGCGTAGCCT
GAATGGCGAATGGGACGCGCCCTGTAGCGGCGCATTAAGCGCGGCGGGTGTGGTGGTTACGCGCAGCGTGACCGC
TACACTTGCCAGCGCCCTAGCGCCCGCTCCTTTCGCTTTCTTCCCTTCCTTTCTCGCCACGTTCGCCGGCTTTCC
CCGTCAAGCTCTAAATCGGGGGCTCCCTTTAGGGTTCCGATTTAGTGCTTTACGGCACCTCGACCCCAAAAAACT
TGATTAGGGTGATGGTTCACGTGGGCCATCGCCCTGATAGACGGTTTTTCGCCCTTTGACGTTGGAGTCCACGTT
CTTTAATAGTGGACTCTTGTTCCAAACTGGAACAACACTCAACCCTATCTCGGTCTATTCTTTTGATTTATAAGG
GATTTTGCCGATTTCGGCCTATTGGTTAAAAAATGAGCTGATTTAACAAAATTTAACGCGAATTTTAACAAAATA
TTAACGTTTACAATTTCAGGTGGCACTTTTCGGGGAAATGTGCGCGGAACCCCTATTTGTTTATTTTTCTAAATA
CATTCAAATATGTATCCGCTCATGAGACAATAACCCTGATAAATGCTTCAATAATATTGAAAAAGGAAGAGTATG
AGTATTCAACATTTCCGTGTCGCCCTTATTCCCTTTTTTGCGGCATTTTGCCTTCCTGTTTTTGCTCACCCAGAA
ACGCTGGTGAAAGTAAAAGATGCTGAAGATCAGTTGGGTGCACGAGTGGGTTACATCGAACTGGATCTCAACAGC
GGTAAGATCCTTGAGAGTTTTCGCCCCGAAGAACGTTTTCCAATGATGAGCACTTTTAAAGTTCTGCTATGTGGC
GCGGTATTATCCCGTATTGACGCCGGGCAAGAGCAACTCGGTCGCCGCATACACTATTCTCAGAATGACTTGGTT
GAGTACTCACCAGTCACAGAAAAGCATCTTACGGATGGCATGACAGTAAGAGAATTATGCAGTGCTGCCATAACC
ATGAGTGATAACACTGCGGCCAACTTACTTCTGACAACGATCGGAGGACCGAAGGAGCTAACCGCTTTTTTGCAC
AACATGGGGGATCATGTAACTCGCCTTGATCGTTGGGAACCGGAGCTGAATGAAGCCATACCAAACGACGAGCGT
GACACCACGATGCCTGTAGCAATGGCAACAACGTTGCGCAAACTATTAACTGGCGAACTACTTACTCTAGCTTCC
CGGCAACAATTAATAGACTGGATGGAGGCGGATAAAGTTGCAGGACCACTTCTGCGCTCGGCCCTTCCGGCTGGC
TGGTTTATTGCTGATAAATCTGGAGCCGGTGAGCGTGGGTCTCGCGGTATCATTGCAGCACTGGGGCCAGATGGT
AAGCCCTCCCGTATCGTAGTTATCTACACGACGGGGAGTCAGGCAACTATGGATGAACGAAATAGACAGATCGCT
GAGATAGGTGCCTCACTGATTAAGCATTGGTAACTGTCAGACCAAGTTTACTCATATATACTTTAGATTGATTTA
AAACTTCATTTTTAATTTAAAAGGATCTAGGTGAAGATCCTTTTTGATAATCTCATGACCAAAATCCCTTAACGT
GAGTTTTCGTTCCACTGAGCGTCAGACCCCGTAGAAAAGATCAAAGGATCTTCTTGAGATCCTTTTTTTCTGCGC
GTAATCTGCTGCTTGCAAACAAAAAAACCACCGCTACCAGCGGTGGTTTGTTTGCCGGATCAAGAGCTACCAACT
CTTTTTCCGAAGGTAACTGGCTTCAGCAGAGCGCAGATACCAAATACTGTCCTTCTAGTGTAGCCGTAGTTAGGC
CACCACTTCAAGAACTCTGTAGCACCGCCTACATACCTCGCTCTGCTAATCCTGTTACCAGTGGCTGCTGCCAGT
GGCGATAAGTCGTGTCTTACCGGGTTGGACTCAAGACGATAGTTACCGGATAAGGCGCAGCGGTCGGGCTGAACG
GGGGGTTCGTGCACACAGCCCAGCTTGGAGCGAACGACCTACACCGAACTGAGATACCTACAGCGTGAGCATTGA
GAAAGCGCCACGCTTCCCGAAGGGAGAAAGGCGGACAGGTATCCGGTAAGCGGCAGGGTCGGAACAGGAGAGCGC
ACGAGGGAGCTTCCAGGGGGAAACGCCTGGTATCTTTATAGTCCTGTCGGGTTTCGCCACCTCTGACTTGAGCGT
CGATTTTTGTGATGCTCGTCAGGGGGGCGGAGCCTATGGAAAAACGCCAGCAACGCGGCCTTTTTACGGTTCCTG
GCCTTTTGCTGGCCTTTTGCTCACATGTTCTTTCCTGCGTTATCCCCTGATTCTGTGGATAACCGTATTACCGCC
TTTGAGTGAGCTGATACCGCTCGCCGCAGCCGAACGACCGAGCGCAGCGAGTCAGTGAGCGAGGAAGCGGAAGAG
CGCCCAATACGCAAACCGCCTCTCCCCGCGCGTTGGCCGATTCATTAATGCAGTAATACATAACCTTATGTATCA
TACACATACGATTTAGGTGACACTATAG

>K-9251 ^{S3K64A}rP2X7^{S3K64A}rP2X7^{S3K64A}rP2X7 ($^{ko-ko-ko}$trimer) 216.437 Da, pI 8.26, 1.911 residues

AATACAAGCTTACAACAAAGAACAACAAACAACAAAGTCCGACGTCGAAGTAGCCACCTACCATCATCATCATCA
TCCCATGGGGAGCGCTTGGAGCCACCCGCAGTTCGAAAAAGGTGGAGGTTCTGGCGGTGGATCGGGAGGTTCAGC
GTGGAGCCACCCGCAGTTCGAGAAAGCTCCCGCTTGCTGTAGCTGGAACGATGTCTTTCAGTATGAGACAAACAA
AGTCACCCGGATCCAGAGCGTGAATTACGGCACCATCAAGTGGATCTTGCACATGACAGTCTTTTCCTACGTTAG
CTTTGCTTTGATGAGCGACAAGCTATATCAGCGGAAGGAGCCCCTTATCAGCTCTGTGCACACCGCTGTCAAAGG
CGTTGCAGAGGTGACAGAGAATGTCACGGAGGGCGGGGTGACGAAGTTAGTACACGGCATCTTCGACACGGCCGA
CTACACCCTCCCTTTGCAGGGGAACTCGTTCTTTGTAATGACAAATTATCTCAAGTCAGAAGGCCAAGAACAGAA
GCTGTGTCCTGAGTATCCCAGCCGCGGTAAACAGTGCCATTCTGACCAGGGTTGTATAAAAGGATGGATGGACCC
ACAAAGTAAAGGAATCCAGACCGGCAGGTGTATACCTTACGACCAGAAGAGGAAGACCTGTGAAATCTTTGCCTG
GTGTCCTGCTGAGGAAGGGAAAGAAGCCCCACGGCCTGCACTCTTGAGGAGCGCCGAAAACTTCACCGTACTCAT
CAAGAACAATATCGACTTCCCGGGCCACAACTATACTACGAGAAACATCTTACCAGGTATGAACATCTCTTGTAC
CTTTCACAAGACTTGGAACCCTCAGTGTCCCATCTTCCGGCTAGGGGACATCTTCCAGGAAATCGGAGAGAACTT
TACAGAGGTGGCAGTTCAGGGAGGAATCATGGGCATTGAGATCTACTGGGACTGCAACCTGGACAGCTGGTCCCA
TCGCTGTCAACCCAAATACAGCTTCCGCCGGCTGGACGACAAGTACACCAATGAGTCCCTGTTCCCTGGCTACAA
CTTCAGATACGCCAAGTACTATAAGGAAAATGGCATGGAAAAGCGGACATTGATCAAAGCCTTCGGCGTGCGTTT
TGACATCCTGGTTTTTGGCACTGGAGGAAAGTTTGACATCATCCAGTTGGTTGTGTACATTGGATCCACCCTGTC
CTATTTCGGTTTGGCCACCGTGTGTATTGACTTGATCATCAACACGTATGCCAGTACCTGCTGCAGGTCACGTGT
TTACCCCTCCTGTAAGTGCTGCGAGCCCTGTGCAGTGAATGAGTACTACTACAGAAAGAAGTGTGAGCCCATCGT
GGAGCCCAAGCCGACGTTAAAGTATGTGTCCTTTGTGGACGAGCCCCACATTTGGATGGTGGACCAGCAGCTGCT
TGGGAAAAGTCTGCAAGATGTCAAAGGTCAAGAGGTCCCGAGACCCCAGACGGACTTCTTGGAACTGTCTAGGCT
CTCCCTCTCTCTCCACCACTCACCCCCAATTCCTGGACAACCTGAGGAAATGCAGCTGCTCCAGATAGAAGCGGT
TCCTAGGTCCAGGGACAGCCCAGATTGGTGCCAGTGTGGAAACTGCCTCCCGTCTCAACTACCAGAGAACCGCAG
GGCCCTGGAGGAGCTGTGCTGCCGGAGGAAGCCAGGACAGTGCATCACTACCTCTGAGCTCTTCAGTAAGATCGT
GCTATCCAGAGAGGCCCTGCAGCTCCTCCTGCTCTACCAGGAGCCCTTGCTGGCGCTGGAGGGAGAGGCCATCAA
CAGCAAGCTGCGACACTGTGCGTACAGGAGCTATGCCACCTGGCGCTTTGTCTCCCAAGACATGGCCGACTTTGC
CATTCTGCCCAGCTGCTGCCGCTGGAAGATCCGGAAGGAGTTCCCCAAGACCCAGGGGCAGTACTCTGGCTTCAA
GTATCCCTACGGTGGATCTGGTGGCGGTGGATCAGGAGGCGGTGGATCAGGACTCATGGGGAGCGCTTGGAGCCA
CCCGCAGTTCGAAAAAGGTGGAGGTTCTGGCGGTGGATCGGGAGGTTCAGCGTGGAGCCACCCGCAGTTCGAGAA
AGCTCCCGCTTGCTGTAGCTGGAACGATGTCTTTCAGTATGAGACAAACAAAGTCACCCGGATCCAGAGCGTGAA
TTACGGCACCATCAAGTGGATCTTGCACATGACAGTCTTTTCCTACGTTAGCTTTGCTTTGATGAGCGACAAGCT
ATATCAGCGGAAGGAGCCCCTTATCAGCTCTGTGCACACCGCTGTCAAAGGCGTTGCAGAGGTGACAGAGAATGT
CACGGAGGGCGGGGTGACGAAGTTAGTACACGGCATCTTCGACACGGCCGACTACACCCTCCCTTTGCAGGGGAA
CTCGTTCTTTGTAATGACAAATTATCTCAAGTCAGAAGGCCAAGAACAGAAGCTGTGTCCTGAGTATCCCAGCCG
CGGTAAACAGTGCCATTCTGACCAGGGTTGTATAAAAGGATGGATGGACCCACAAAGTAAAGGAATCCAGACCGG
CAGGTGTATACCTTACGACCAGAAGAGGAAGACCTGTGAAATCTTTGCCTGGTGTCCTGCTGAGGAAGGGAAAGA
AGCCCCACGGCCTGCACTCTTGAGGAGCGCCGAAAACTTCACCGTACTCATCAAGAACAATATCGACTTCCCGGG
CCACAACTATACTACGAGAAACATCTTACCAGGTATGAACATCTCTTGTACCTTTCACAAGACTTGGAACCCTCA
GTGTCCCATCTTCCGGCTAGGGGACATCTTCCAGGAAATCGGAGAGAACTTTACAGAGGTGGCAGTTCAGGGAGG
AATCATGGGCATTGAGATCTACTGGGACTGCAACCTGGACAGCTGGTCCCATCGCTGTCAACCCAAATACAGCTT
CCGCCGGCTGGACGACAAGTACACCAATGAGTCCCTGTTCCCTGGCTACAACTTCAGATACGCCAAGTACTATAA
GGAAAATGGCATGGAAAAGCGGACATTGATCAAAGCCTTCGGCGTGCGTTTTGACATCCTGGTTTTTGGCACTGG
AGGAAAGTTTGACATCATCCAGTTGGTTGTGTACATTGGATCCACCCTGTCCTATTTCGGTTTGGCCACCGTGTG
TATTGACTTGATCATCAACACGTATGCCAGTACCTGCTGCAGGTCACGTGTTTACCCCTCCTGTAAGTGCTGCGA
GCCCTGTGCAGTGAATGAGTACTACTACAGAAAGAAGTGTGAGCCCATCGTGGAGCCCAAGCCGACGTTAAAGTA
TGTGTCCTTTGTGGACGAGCCCCACATTTGGATGGTGGACCAGCAGCTGCTTGGGAAAAGTCTGCAAGATGTCAA
AGGTCAAGAGGTCCCGAGACCCCAGACGGACTTCTTGGAACTGTCTAGGCTCTCCCTCTCTCTCCACCACTCACC
CCCAATTCCTGGACAACCTGAGGAAATGCAGCTGCTCCAGATAGAAGCGGTTCCTAGGTCCAGGGACAGCCCAGA
TTGGTGCCAGTGTGGAAACTGCCTCCCGTCTCAACTACCAGAGAACCGCAGGGCCCTGGAGGAGCTGTGCTGCCG
GAGGAAGCCAGGACAGTGCATCACTACCTCTGAGCTCTTCAGTAAGATCGTGCTATCCAGAGAGGCCCTGCAGCT
CCTCCTGCTCTACCAGGAGCCCTTGCTGGCGCTGGAGGGAGAGGCCATCAACAGCAAGCTGCGACACTGTGCGTA
CAGGAGCTATGCCACCTGGCGCTTTGTCTCCCAAGACATGGCCGACTTTGCCATTCTGCCCAGCTGCTGCCGCTG
GAAGATCCGGAAGGAGTTCCCCAAGACCCAGGGGCAGTACTCTGGCTTCAAGTATCCCTACGGTGGATCTGGTGG
CGGTGGATCAGGAGGCGGTGGATCAGGACTCATGGGGAGCGCTTGGAGCCACCCGCAGTTCGAAAAAGGTGGAGG
TTCTGGCGGTGGATCGGGAGGTTCAGCGTGGAGCCACCCGCAGTTCGAGAAAGCTCCCGCTTGCTGTAGCTGGAA
CGATGTCTTTCAGTATGAGACAAACAAAGTCACCCGGATCCAGAGCGTGAATTACGGCACCATCAAGTGGATCTT
GCACATGACAGTCTTTTCCTACGTTAGCTTTGCTTTGATGAGCGACAAGCTATATCAGCGGAAGGAGCCCCTTAT
CAGCTCTGTGCACACCGCTGTCAAAGGCGTTGCAGAGGTGACAGAGAATGTCACGGAGGGCGGGGTGACGAAGTT
AGTACACGGCATCTTCGACACGGCCGACTACACCCTCCCTTTGCAGGGGAACTCGTTCTTTGTAATGACAAATTA

TCTCAAGTCAGAAGGCCAAGAACAGAAGCTGTGTCCTGAGTATCCCAGCCGCGGTAAACAGTGCCATTCTGACCA
GGGTTGTATAAAAGGATGGATGGACCCACAAAGTAAAGGAATCCAGACCGGCAGGTGTATACCTTACGACCAGAA
GAGGAAGACCTGTGAAATCTTTGCCTGGTGTCCTGCTGAGGAAGGGAAAGAAGCCCCACGGCCTGCACTCTTGAG
GAGCGCCGAAAACTTCACCGTACTCATCAAGAACAATATCGACTTCCCGGGCCACAACTATACTACGAGAAACAT
CTTACCAGGTATGAACATCTCTTGTACCTTTCACAAGACTTGGAACCCTCAGTGTCCCATCTTCCGGCTAGGGGA
CATCTTCCAGGAAATCGGAGAGAACTTTACAGAGGTGGCAGTTCAGGGAGGAATCATGGGCATTGAGATCTACTG
GGACTGCAACCTGGACAGCTGGTCCCATCGCTGTCAACCCAAATACAGCTTCCGCCGGCTGGACGACAAGTACAC
CAATGAGTCCCTGTTCCCTGGCTACAACTTCAGATACGCCAAGTACTATAAGGAAAATGGCATGGAAAAGCGGAC
ATTGATCAAAGCCTTCGGCGTGCGTTTTGACATCCTGGTTTTTGGCACTGGAGGAAAGTTTGACATCATCCAGTT
GGTTGTGTACATTGGATCCACCCTGTCCTATTTCGGTTTGGCCACCGTGTGTATTGACTTGATCATCAACACGTA
TGCCAGTACCTGCTGCAGGTCACGTGTTTACCCCTCCTGTAAGTGCTGCGAGCCCTGTGCAGTGAATGAGTACTA
CTACAGAAAGAAGTGTGAGCCCATCGTGGAGCCCAAGCCGACGTTAAAGTATGTGTCCTTTGTGGACGAGCCCCA
CATTTGGATGGTGGACCAGCAGCTGCTTGGGAAAAGTCTGCAAGATGTCAAAGGTCAAGAGGTCCCGAGACCCCA
GACGGACTTCTTGGAACTGTCTAGGCTCTCCCTCTCTCTCCACCACTCACCCCCAATTCCTGGACAACCTGAGGA
AATGCAGCTGCTCCAGATAGAAGCGGTTCCTAGGTCCAGGGACAGCCCAGATTGGTGCCAGTGTGGAAACTGCCT
CCCGTCTCAACTACCAGAGAACCGCAGGGCCCTGGAGGAGCTGTGCTGCCGGAGGAAGCCAGGACAGTGCATCAC
TACCTCTGAGCTCTTCAGTAAGATCGTGCTATCCAGAGAGGCCCTGCAGCTCCTCCTGCTCTACCAGGAGCCCTT
GCTGGCGCTGGAGGGAGAGGCCATCAACAGCAAGCTGCGACACTGTGCGTACAGGAGCTATGCCACCTGGCGCTT
TGTCTCCCAAGACATGGCCGACTTTGCCATTCTGCCCAGCTGCTGCCGCTGGAAGATCCGGAAGGAGTTCCCCAA
GACCCAGGGGCAGTACTCTGGCTTCAAGTATCCCTACTGACTATCTAGAGGATCCCCGGGTACGTACCGGGCCCC
CCATCGAGGTCGACGTAGCTTTCATTTGTTTT**AA**TTT**A**TTTTTT**AAA**T**A**GC**A**TT**A**C**AAAA**C**AAA**TT**A**CC**A**C**AA**C**A**
AAAAAAAAACC**AAAA**C**AAAAA**C**AAA**T**AA**T**AAA**GTCCC**AAAA**C**AAAAA**CGG**AA**T**A**TGC**AAA**C**AAAAAAAAAAAAAA**
AAAAAAAAAAAAAAAAAAAAAAAAAAAAAAAAAAAAAGAATTGCTCGAGCGGCCGCTCGAGCAATTCCGGTCTCC
CTATAGTGAGTCGTATTACTGGCGTAATAGCGAAGAGGCCCGCACCGATCGCCCTTCCCAACAGTTGCGTAGCCT
GAATGGCGAATGGGACGCGCCCTGTAGCGGCGCATTAAGCGCGGCGGGTGTGGTGGTTACGCGCAGCGTGACCGC
TACACTTGCCAGCGCCCTAGCGCCCGCTCCTTTCGCTTTCTTCCCTTCCTTTCTCGCCACGTTCGCCGGCTTTCC
CCGTCAAGCTCTAAATCGGGGGCTCCCTTTAGGGTTCCGATTTAGTGCTTTACGGCACCTCGACCCCAAAAAACT
TGATTAGGGTGATGGTTCACGTGGGCCATCGCCCTGATAGACGGTTTTTCGCCCTTTGACGTTGGAGTCCACGTT
CTTTAATAGTGGACTCTTGTTCCAAACTGGAACAACACTCAACCCTATCTCGGTCTATTCTTTTGATTTATAAGG
GATTTTGCCGATTTCGGCCTATTGGTTAAAAAATGAGCTGATTTAACAAAATTTAACGCGAATTTTAACAAAATA
TTAACGTTTACAATTTCAGGTGGCACTTTTCGGGGAAATGTGCGCGGAACCCCTATTTGTTTATTTTTCTAAATA
CATTCAAATATGTATCCGCTCATGAGACAATAACCCTGATAAATGCTTCAATAATATTGAAAAAGGAAGAGTATG
AGTATTCAACATTTCCGTGTCGCCCTTATTCCCTTTTTTGCGGCATTTTGCCTTCCTGTTTTTGCTCACCCAGAA
ACGCTGGTGAAAGTAAAAGATGCTGAAGATCAGTTGGGTGCACGAGTGGGTTACATCGAACTGGATCTCAACAGC
GGTAAGATCCTTGAGAGTTTTCGCCCCGAAGAACGTTTTCCAATGATGAGCACTTTTAAAGTTCTGCTATGTGGC
GCGGTATTATCCCGTATTGACGCCGGGCAAGAGCAACTCGGTCGCCGCATACACTATTCTCAGAATGACTTGGTT
GAGTACTCACCAGTCACAGAAAAGCATCTTACGGATGGCATGACAGTAAGAGAATTATGCAGTGCTGCCATAACC
ATGAGTGATAACACTGCGGCCAACTTACTTCTGACAACGATCGGAGGACCGAAGGAGCTAACCGCTTTTTTGCAC
AACATGGGGGATCATGTAACTCGCCTTGATCGTTGGGAACCGGAGCTGAATGAAGCCATACCAAACGACGAGCGT
GACACCACGATGCCTGTAGCAATGGCAACAACGTTGCGCAAACTATTAACTGGCGAACTACTTACTCTAGCTTCC
CGGCAACAATTAATAGACTGGATGGAGGCGGATAAAGTTGCAGGACCACTTCTGCGCTCGGCCCTTCCGGCTGGC
TGGTTTATTGCTGATAAATCTGGAGCCGGTGAGCGTGGGTCTCGCGGTATCATTGCAGCACTGGGGCCAGATGGT
AAGCCCTCCCGTATCGTAGTTATCTACACGACGGGGAGTCAGGCAACTATGGATGAACGAAATAGACAGATCGCT
GAGATAGGTGCCTCACTGATTAAGCATTGGTAACTGTCAGACCAAGTTTACTCATATATACTTTAGATTGATTTA
AAACTTCATTTTTAATTTAAAAGGATCTAGGTGAAGATCCTTTTTGATAATCTCATGACCAAAATCCCTTAACGT
GAGTTTTCGTTCCACTGAGCGTCAGACCCCGTAGAAAAGATCAAAGGATCTTCTTGAGATCCTTTTTTTCTGCGC
GTAATCTGCTGCTTGCAAACAAAAAAACCACCGCTACCAGCGGTGGTTTGTTTGCCGGATCAAGAGCTACCAACT
CTTTTTCCGAAGGTAACTGGCTTCAGCAGAGCGCAGATACCAAATACTGTCCTTCTAGTGTAGCCGTAGTTAGGC
CACCACTTCAAGAACTCTGTAGCACCGCCTACATACCTCGCTCTGCTAATCCTGTTACCAGTGGCTGCTGCCAGT
GGCGATAAGTCGTGTCTTACCGGGTTGGACTCAAGACGATAGTTACCGGATAAGGCGCAGCGGTCGGGCTGAACG
GGGGGTTCGTGCACACAGCCCAGCTTGGAGCGAACGACCTACACCGAACTGAGATACCTACAGCGTGAGCATTGA
GAAAGCGCCACGCTTCCCGAAGGGAGAAAGGCGGACAGGTATCCGGTAAGCGGCAGGGTCGGAACAGGAGAGCGC
ACGAGGGAGCTTCCAGGGGGAAACGCCTGGTATCTTTATAGTCCTGTCGGGTTTCGCCACCTCTGACTTGAGCGT
CGATTTTTGTGATGCTCGTCAGGGGGGCGGAGCCTATGGAAAAACGCCAGCAACGCGGCCTTTTTACGGTTCCTG
GCCTTTTGCTGGCCTTTTGCTCACATGTTCTTTCCTGCGTTATCCCCTGATTCTGTGGATAACCGTATTACCGCC
TTTGAGTGAGCTGATACCGCTCGCCGCAGCCGAACGACCGAGCGCAGCGAGTCAGTGAGCGAGGAAGCGGAAGAG
CGCCCAATACGCAAACCGCCTCTCCCCGCGCGTTGGCCGATTCATTAATGCAGTAATACATAACCTTATGTATCA
TACACATACGATTTAGGTGACACTATAG

>K-9252 ^{S3}rP2X7^{S3}rP2X7^{S3K64A}rP2X7-pNKS4 ($^{wt-wt-ko}$trimer) 216.552 Da, pI 8.32, 1.911 residues

AATACAAGCTTACAACAAAGAACAACAAACAACAAAGTCCGACGTCGAAGTAGCCACCTACCATCATCATCATCA
TCCCATGGGGAGCGCTTGGAGCCACCCGCAGTTCGAAAAAGGTGGAGGTTCTGGCGGTGGATCGGGAGGTTCAGC
GTGGAGCCACCCGCAGTTCGAGAAAGCTCCCGCTTGCTGTAGCTGGAACGATGTCTTTCAGTATGAGACAAACAA
AGTCACCCGGATCCAGAGCGTGAATTACGGCACCATCAAGTGGATCTTGCACATGACAGTCTTTTCCTACGTTAG
CTTTGCTTTGATGAGCGACAAGCTATATCAGCGGAAGGAGCCCCTTATCAGCTCTGTGCACACCAAGGTCAAAGG
CGTTGCAGAGGTGACAGAGAATGTCACGGAGGGCGGGGTGACGAAGTTAGTACACGGCATCTTCGACACGGCCGA
CTACACCCTCCCTTTGCAGGGGAACTCGTTCTTTGTAATGACAAATTATCTCAAGTCAGAAGGCCAAGAACAGAA
GCTGTGTCCTGAGTATCCCAGCCGCGGTAAACAGTGCCATTCTGACCAGGGTTGTATAAAAGGATGGATGGACCC
ACAAAGTAAAGGAATCCAGACCGGCAGGTGTATACCTTACGACCAGAAGAGGAAGACCTGTGAAATCTTTGCCTG
GTGTCCTGCTGAGGAAGGGAAAGAAGCCCCACGGCCTGCACTCTTGAGGAGCGCCGAAAACTTCACCGTACTCAT
CAAGAACAATATCGACTTCCCGGGCCACAACTATACTACGAGAAACATCTTACCAGGTATGAACATCTCTTGTAC
CTTTCACAAGACTTGGAACCCTCAGTGTCCCATCTTCCGGCTAGGGGACATCTTCCAGGAAATCGGAGAGAACTT
TACAGAGGTGGCAGTTCAGGGAGGAATCATGGGCATTGAGATCTACTGGGACTGCAACCTGGACAGCTGGTCCCA
TCGCTGTCAACCCAAATACAGCTTCCGCCGGCTGGACGACAAGTACACCAATGAGTCCCTGTTCCCTGGCTACAA
CTTCAGATACGCCAAGTACTATAAGGAAAATGGCATGGAAAAGCGGACATTGATCAAAGCCTTCGGCGTGCGTTT
TGACATCCTGGTTTTTGGCACTGGAGGAAAGTTTGACATCATCCAGTTGGTTGTGTACATTGGATCCACCCTGTC
CTATTTCGGTTTGGCCACCGTGTGTATTGACTTGATCATCAACACGTATGCCAGTACCTGCTGCAGGTCACGTGT
TTACCCCTCCTGTAAGTGCTGCGAGCCCTGTGCAGTGAATGAGTACTACTACAGAAAGAAGTGTGAGCCCATCGT
GGAGCCCAAGCCGACGTTAAAGTATGTGTCCTTTGTGGACGAGCCCCACATTTGGATGGTGGACCAGCAGCTGCT
TGGGAAAAGTCTGCAAGATGTCAAAGGTCAAGAGGTCCCGAGACCCCAGACGGACTTCTTGGAACTGTCTAGGCT
CTCCCTCTCTCTCCACCACTCACCCCCAATTCCTGGACAACCTGAGGAAATGCAGCTGCTCCAGATAGAAGCGGT
TCCTAGGTCCAGGGACAGCCCAGATTGGTGCCAGTGTGGAAACTGCCTCCCGTCTCAACTACCAGAGAACCGCAG
GGCCCTGGAGGAGCTGTGCTGCCGGAGGAAGCCAGGACAGTGCATCACTACCTCTGAGCTCTTCAGTAAGATCGT
GCTATCCAGAGAGGCCCTGCAGCTCCTCCTGCTCTACCAGGAGCCCTTGCTGGCGCTGGAGGGAGAGGCCATCAA
CAGCAAGCTGCGACACTGTGCGTACAGGAGCTATGCCACCTGGCGCTTTGTCTCCCAAGACATGGCCGACTTTGC
CATTCTGCCCAGCTGCTGCCGCTGGAAGATCCGGAAGGAGTTCCCCAAGACCCAGGGGCAGTACTCTGGCTTCAA
GTATCCCTACGGTGGATCTGGTGGCGGTGGATCAGGAGGCGGTGGATCAGGACTCATGGGGAGCGCTTGGAGCCA
CCCGCAGTTCGAAAAAGGTGGAGGTTCTGGCGGTGGATCGGGAGGTTCAGCGTGGAGCCACCCGCAGTTCGAGAA
AGCTCCCGCTTGCTGTAGCTGGAACGATGTCTTTCAGTATGAGACAAACAAAGTCACCCGGATCCAGAGCGTGAA
TTACGGCACCATCAAGTGGATCTTGCACATGACAGTCTTTTCCTACGTTAGCTTTGCTTTGATGAGCGACAAGCT
ATATCAGCGGAAGGAGCCCCTTATCAGCTCTGTGCACACCAAGGTCAAAGGCGTTGCAGAGGTGACAGAGAATGT
CACGGAGGGCGGGGTGACGAAGTTAGTACACGGCATCTTCGACACGGCCGACTACACCCTCCCTTTGCAGGGGAA
CTCGTTCTTTGTAATGACAAATTATCTCAAGTCAGAAGGCCAAGAACAGAAGCTGTGTCCTGAGTATCCCAGCCG
CGGTAAACAGTGCCATTCTGACCAGGGTTGTATAAAAGGATGGATGGACCCACAAAGTAAAGGAATCCAGACCGG
CAGGTGTATACCTTACGACCAGAAGAGGAAGACCTGTGAAATCTTTGCCTGGTGTCCTGCTGAGGAAGGGAAAGA
AGCCCCACGGCCTGCACTCTTGAGGAGCGCCGAAAACTTCACCGTACTCATCAAGAACAATATCGACTTCCCGGG
CCACAACTATACTACGAGAAACATCTTACCAGGTATGAACATCTCTTGTACCTTTCACAAGACTTGGAACCCTCA
GTGTCCCATCTTCCGGCTAGGGGACATCTTCCAGGAAATCGGAGAGAACTTTACAGAGGTGGCAGTTCAGGGAGG
AATCATGGGCATTGAGATCTACTGGGACTGCAACCTGGACAGCTGGTCCCATCGCTGTCAACCCAAATACAGCTT
CCGCCGGCTGGACGACAAGTACACCAATGAGTCCCTGTTCCCTGGCTACAACTTCAGATACGCCAAGTACTATAA
GGAAAATGGCATGGAAAAGCGGACATTGATCAAAGCCTTCGGCGTGCGTTTTGACATCCTGGTTTTTGGCACTGG
AGGAAAGTTTGACATCATCCAGTTGGTTGTGTACATTGGATCCACCCTGTCCTATTTCGGTTTGGCCACCGTGTG
TATTGACTTGATCATCAACACGTATGCCAGTACCTGCTGCAGGTCACGTGTTTACCCCTCCTGTAAGTGCTGCGA
GCCCTGTGCAGTGAATGAGTACTACTACAGAAAGAAGTGTGAGCCCATCGTGGAGCCCAAGCCGACGTTAAAGTA
TGTGTCCTTTGTGGACGAGCCCCACATTTGGATGGTGGACCAGCAGCTGCTTGGGAAAAGTCTGCAAGATGTCAA
AGGTCAAGAGGTCCCGAGACCCCAGACGGACTTCTTGGAACTGTCTAGGCTCTCCCTCTCTCTCCACCACTCACC
CCCAATTCCTGGACAACCTGAGGAAATGCAGCTGCTCCAGATAGAAGCGGTTCCTAGGTCCAGGGACAGCCCAGA
TTGGTGCCAGTGTGGAAACTGCCTCCCGTCTCAACTACCAGAGAACCGCAGGGCCCTGGAGGAGCTGTGCTGCCG
GAGGAAGCCAGGACAGTGCATCACTACCTCTGAGCTCTTCAGTAAGATCGTGCTATCCAGAGAGGCCCTGCAGCT
CCTCCTGCTCTACCAGGAGCCCTTGCTGGCGCTGGAGGGAGAGGCCATCAACAGCAAGCTGCGACACTGTGCGTA
CAGGAGCTATGCCACCTGGCGCTTTGTCTCCCAAGACATGGCCGACTTTGCCATTCTGCCCAGCTGCTGCCGCTG
GAAGATCCGGAAGGAGTTCCCCAAGACCCAGGGGCAGTACTCTGGCTTCAAGTATCCCTACGGTGGATCTGGTGG
CGGTGGATCAGGAGGCGGTGGATCAGGACTCATGGGGAGCGCTTGGAGCCACCCGCAGTTCGAAAAAGGTGGAGG
TTCTGGCGGTGGATCGGGAGGTTCAGCGTGGAGCCACCCGCAGTTCGAGAAAGCTCCCGCTTGCTGTAGCTGGAA

CGATGTCTTTCAGTATGAGACAAACAAAGTCACCCGGATCCAGAGCGTGAATTACGGCACCATCAAGTGGATCTT
GCACATGACAGTCTTTTCCTACGTTAGCTTTGCTTTGATGAGCGACAAGCTATATCAGCGGAAGGAGCCCCTTAT
CAGCTCTGTGCACACCGCTGTCAAAGGCGTTGCAGAGGTGACAGAGAATGTCACGGAGGGCGGGGTGACGAAGTT
AGTACACGGCATCTTCGACACGGCCGACTACACCCTCCCTTTGCAGGGGAACTCGTTCTTTGTAATGACAAATTA
TCTCAAGTCAGAAGGCCAAGAACAGAAGCTGTGTCCTGAGTATCCCAGCCGCGGTAAACAGTGCCATTCTGACCA
GGGTTGTATAAAAGGATGGATGGACCCACAAAGTAAAGGAATCCAGACCGGCAGGTGTATACCTTACGACCAGAA
GAGGAAGACCTGTGAAATCTTTGCCTGGTGTCCTGCTGAGGAAGGGAAAGAAGCCCCACGGCCTGCACTCTTGAG
GAGCGCCGAAAACTTCACCGTACTCATCAAGAACAATATCGACTTCCCGGGCCACAACTATACTACGAGAAACAT
CTTACCAGGTATGAACATCTCTTGTACCTTTCACAAGACTTGGAACCCTCAGTGTCCCATCTTCCGGCTAGGGGA
CATCTTCCAGGAAATCGGAGAGAACTTTACAGAGGTGGCAGTTCAGGGAGGAATCATGGGCATTGAGATCTACTG
GGACTGCAACCTGGACAGCTGGTCCCATCGCTGTCAACCCAAATACAGCTTCCGCCGGCTGGACGACAAGTACAC
CAATGAGTCCCTGTTCCCTGGCTACAACTTCAGATACGCCAAGTACTATAAGGAAAATGGCATGGAAAAGCGGAC
ATTGATCAAAGCCTTCGGCGTGCGTTTTGACATCCTGGTTTTTGGCACTGGAGGAAAGTTTGACATCATCCAGTT
GGTTGTGTACATTGGATCCACCCTGTCCTATTTCGGTTTGGCCACCGTGTGTATTGACTTGATCATCAACACGTA
TGCCAGTACCTGCTGCAGGTCACGTGTTTACCCCTCCTGTAAGTGCTGCGAGCCCTGTGCAGTGAATGAGTACTA
CTACAGAAAGAAGTGTGAGCCCATCGTGGAGCCCAAGCCGACGTTAAAGTATGTGTCCTTTGTGGACGAGCCCCA
CATTTGGATGGTGGACCAGCAGCTGCTTGGGAAAAGTCTGCAAGATGTCAAAGGTCAAGAGGTCCCGAGACCCCA
GACGGACTTCTTGGAACTGTCTAGGCTCTCCCTCTCTCTCCACCACTCACCCCCAATTCCTGGACAACCTGAGGA
AATGCAGCTGCTCCAGATAGAAGCGGTTCCTAGGTCCAGGGACAGCCCAGATTGGTGCCAGTGTGGAAACTGCCT
CCCGTCTCAACTACCAGAGAACCGCAGGGCCCTGGAGGAGCTGTGCTGCCGGAGGAAGCCAGGACAGTGCATCAC
TACCTCTGAGCTCTTCAGTAAGATCGTGCTATCCAGAGAGGCCCTGCAGCTCCTCCTGCTCTACCAGGAGCCCTT
GCTGGCGCTGGAGGGAGAGGCCATCAACAGCAAGCTGCGACACTGTGCGTACAGGAGCTATGCCACCTGGCGCTT
TGTCTCCCAAGACATGGCCGACTTTGCCATTCTGCCCAGCTGCTGCCGCTGGAAGATCCGGAAGGAGTTCCCCAA
GACCCAGGGGCAGTACTCTGGCTTCAAGTATCCCTACTGACTATCTAGAGGATCCCCGGGTACGTACCGGGCCCC
CCATCGAGGTCGACGTAGCTTTCATTTGTTTT**AA**TTT**A**TTTTTTT**AAA**T**A**GC**A**TT**A**C**AAAA**C**AAA**TT**A**CC**A**C**AA**C**A**
AAAAAAAAACC**AAAA**C**AAAAA**C**AAA**T**AA**T**AAA**GTCCC**AAAA**C**AAAAA**CGG**AA**T**A**TGC**AAA**C**AAAAAAAAAAAAAAA**
AAAAAAAAAAAAAAAAAAAAAAAAAAAAAAAAAAAAAAGAATTGCTCGAGCGGCCGCTCGAGCAATTCCGGTCTCC
CTATAGTGAGTCGTATTACTGGCGTAATAGCGAAGAGGCCCGCACCGATCGCCCTTCCCAACAGTTGCGTAGCCT
GAATGGCGAATGGGACGCGCCCTGTAGCGGCGCATTAAGCGCGGCGGGTGTGGTGGTTACGCGCAGCGTGACCGC
TACACTTGCCAGCGCCCTAGCGCCCGCTCCTTTCGCTTTCTTCCCTTCCTTTCTCGCCACGTTCGCCGGCTTTCC
CCGTCAAGCTCTAAATCGGGGGCTCCCTTTAGGGTTCCGATTTAGTGCTTTACGGCACCTCGACCCCAAAAAACT
TGATTAGGGTGATGGTTCACGTGGGCCATCGCCCTGATAGACGGTTTTTCGCCCTTTGACGTTGGAGTCCACGTT
CTTTAATAGTGGACTCTTGTTCCAAACTGGAACAACACTCAACCCTATCTCGGTCTATTCTTTTGATTTATAAGG
GATTTTGCCGATTTCGGCCTATTGGTTAAAAAATGAGCTGATTTAACAAAAATTTAACGCGAATTTTAACAAAATA
TTAACGTTTACAATTTCAGGTGGCACTTTTCGGGGAAATGTGCGCGGAACCCCTATTTGTTTATTTTTCTAAATA
CATTCAAATATGTATCCGCTCATGAGACAATAACCCTGATAAATGCTTCAATAATATTGAAAAAGGAAGAGTATG
AGTATTCAACATTTCCGTGTCGCCCTTATTCCCTTTTTTGCGGCATTTTGCCTTCCTGTTTTTGCTCACCCAGAA
ACGCTGGTGAAAGTAAAAGATGCTGAAGATCAGTTGGGTGCACGAGTGGGTTACATCGAACTGGATCTCAACAGC
GGTAAGATCCTTGAGAGTTTTCGCCCCGAAGAACGTTTTCCAATGATGAGCACTTTTAAAGTTCTGCTATGTGGC
GCGGTATTATCCCGTATTGACGCCGGGCAAGAGCAACTCGGTCGCCGCATACACTATTCTCAGAATGACTTGGTT
GAGTACTCACCAGTCACAGAAAAGCATCTTACGGATGGCATGACAGTAAGAGAATTATGCAGTGCTGCCATAACC
ATGAGTGATAACACTGCGGCCAACTTACTTCTGACAACGATCGGAGGACCGAAGGAGCTAACCGCTTTTTTGCAC
AACATGGGGGATCATGTAACTCGCCTTGATCGTTGGGAACCGGAGCTGAATGAAGCCATACCAAACGACGAGCGT
GACACCACGATGCCTGTAGCAATGGCAACAACGTTGCGCAAACTATTAACTGGCGAACTACTTACTCTAGCTTCC
CGGCAACAATTAATAGACTGGATGGAGGCGGATAAAGTTGCAGGACCACTTCTGCGCTCGGCCCTTCCGGCTGGC
TGGTTTATTGCTGATAAATCTGGAGCCGGTGAGCGTGGGTCTCGCGGTATCATTGCAGCACTGGGGCCAGATGGT
AAGCCCTCCCGTATCGTAGTTATCTACACGACGGGGAGTCAGGCAACTATGGATGAACGAAATAGACAGATCGCT
GAGATAGGTGCCTCACTGATTAAGCATTGGTAACTGTCAGACCAAGTTTACTCATATATACTTTAGATTGATTTA
AAACTTCATTTTTAATTTAAAAGGATCTAGGTGAAGATCCTTTTTGATAATCTCATGACCAAAATCCCTTAACGT
GAGTTTTCGTTCCACTGAGCGTCAGACCCCGTAGAAAAGATCAAAGGATCTTCTTGAGATCCTTTTTTTCTGCGC
GTAATCTGCTGCTTGCAAACAAAAAAACCACCGCTACCAGCGGTGGTTTGTTTGCCGGATCAAGAGCTACCAACT
CTTTTTCCGAAGGTAACTGGCTTCAGCAGAGCGCAGATACCAAATACTGTCCTTCTAGTGTAGCCGTAGTTAGGC
CACCACTTCAAGAACTCTGTAGCACCGCCTACATACCTCGCTCTGCTAATCCTGTTACCAGTGGCTGCTGCCAGT
GGCGATAAGTCGTGTCTTACCGGGTTGGACTCAAGACGATAGTTACCGGATAAGGCGCAGCGGTCGGGCTGAACG
GGGGGTTCGTGCACACAGCCCAGCTTGGAGCGAACGACCTACACCGAACTGAGATACCTACAGCGTGAGCATTGA
GAAAGCGCCACGCTTCCCGAAGGGAGAAAGGCGGACAGGTATCCGGTAAGCGGCAGGGTCGGAACAGGAGAGCGC
ACGAGGGAGCTTCCAGGGGGAAACGCCTGGTATCTTTATAGTCCTGTCGGGTTTCGCCACCTCTGACTTGAGCGT
CGATTTTTGTGATGCTCGTCAGGGGGGCGGAGCCTATGGAAAAACGCCAGCAACGCGGCCTTTTTACGGTTCCTG

GCCTTTTGCTGGCCTTTTGCTCACATGTTCTTTCCTGCGTTATCCCCTGATTCTGTGGATAACCGTATTACCGCC
TTTGAGTGAGCTGATACCGCTCGCCGCAGCCGAACGACCGAGCGCAGCGAGTCAGTGAGCGAGGAAGCGGAAGAG
CGCCCAATACGCAAACCGCCTCTCCCCGCGCGTTGGCCGATTCATTAATGCAGTAATACATAACCTTATGTATCA
TACACATACGATTTAGGTGACACTATAG

>K-9253 S3K64ArP2X7S3rP2X7S3rP2X7-pNKS4 (ko-wt-wttrimer) 216.551 Da, pI 8.32, 1.911 residues

AATACAAGCTTACAACAAAGAACAACAAACAACAAAGTCCGACGTCGAAGTAGCCACCTACCATCATCATCATCA
TCCCATGGGGAGCGCTTGGAGCCACCCGCAGTTCGAAAAAGGTGGAGGTTCTGGCGGTGGATCGGGAGGTTCAGC
GTGGAGCCACCCGCAGTTCGAGAAAGCTCCCGCTTGCTGTAGCTGGAACGATGTCTTTCAGTATGAGACAAACAA
AGTCACCCGGATCCAGAGCGTGAATTACGGCACCATCAAGTGGATCTTGCACATGACAGTCTTTTCCTACGTTAG
CTTTGCTTTGATGAGCGACAAGCTATATCAGCGGAAGGAGCCCCTTATCAGCTCTGTGCACACCGCTGTCAAAGG
CGTTGCAGAGGTGACAGAGAATGTCACGGAGGGCGGGGTGACGAAGTTAGTACACGGCATCTTCGACACGGCCGA
CTACACCCTCCCTTTGCAGGGGAACTCGTTCTTTGTAATGACAAATTATCTCAAGTCAGAAGGCCAAGAACAGAA
GCTGTGTCCTGAGTATCCCAGCCGCGGTAAACAGTGCCATTCTGACCAGGGTTGTATAAAAGGATGGATGGACCC
ACAAAGTAAAGGAATCCAGACCGGCAGGTGTATACCTTACGACCAGAAGAGGAAGACCTGTGAAATCTTTGCCTG
GTGTCCTGCTGAGGAAGGGAAAGAAGCCCCACGGCCTGCACTCTTGAGGAGCGCCGAAAACTTCACCGTACTCAT
CAAGAACAATATCGACTTCCCGGGCCACAACTATACTACGAGAAACATCTTACCAGGTATGAACATCTCTTGTAC
CTTTCACAAGACTTGGAACCCTCAGTGTCCCATCTTCCGGCTAGGGGACATCTTCCAGGAAATCGGAGAGAACTT
TACAGAGGTGGCAGTTCAGGGAGGAATCATGGGCATTGAGATCTACTGGGACTGCAACCTGGACAGCTGGTCCCA
TCGCTGTCAACCCAAATACAGCTTCCGCCGGCTGGACGACAAGTACACCAATGAGTCCCTGTTCCCTGGCTACAA
CTTCAGATACGCCAAGTACTATAAGGAAAATGGCATGGAAAAGCGGACATTGATCAAAGCCTTCGGCGTGCGTTT
TGACATCCTGGTTTTTGGCACTGGAGGAAAGTTTGACATCATCCAGTTGGTTGTGTACATTGGATCCACCCTGTC
CTATTTCGGTTTGGCCACCGTGTGTATTGACTTGATCATCAACACGTATGCCAGTACCTGCTGCAGGTCACGTGT
TTACCCCTCCTGTAAGTGCTGCGAGCCCTGTGCAGTGAATGAGTACTACTACAGAAAGAAGTGTGAGCCCATCGT
GGAGCCCAAGCCGACGTTAAAGTATGTGTCCTTTGTGGACGAGCCCCACATTTGGATGGTGGACCAGCAGCTGCT
TGGGAAAAGTCTGCAAGATGTCAAAGGTCAAGAGGTCCCGAGACCCCAGACGGACTTCTTGGAACTGTCTAGGCT
CTCCCTCTCTCTCCACCACTCACCCCCAATTCCTGGACAACCTGAGGAAATGCAGCTGCTCCAGATAGAAGCGGT
TCCTAGGTCCAGGGACAGCCCAGATTGGTGCCAGTGTGGAAACTGCCTCCCGTCTCAACTACCAGAGAACCGCAG
GGCCCTGGAGGAGCTGTGCTGCCGGAGGAAGCCAGGACAGTGCATCACTACCTCTGAGCTCTTCAGTAAGATCGT
GCTATCCAGAGAGGCCCTGCAGCTCCTCCTGCTCTACCAGGAGCCCTTGCTGGCGCTGGAGGGAGAGGCCATCAA
CAGCAAGCTGCGACACTGTGCGTACAGGAGCTATGCCACCTGGCGCTTTGTCTCCCAAGACATGGCCGACTTTGC
CATTCTGCCCAGCTGCTGCCGCTGGAAGATCCGGAAGGAGTTCCCCAAGACCCAGGGGCAGTACTCTGGCTTCAA
GTATCCCTACGGTGGATCTGGTGGCGGTGGATCAGGAGGCGGTGGATCAGGACTCATGGGGAGCGCTTGGAGCCA
CCCGCAGTTCGAAAAAGGTGGAGGTTCTGGCGGTGGATCGGGAGGTTCAGCGTGGAGCCACCCGCAGTTCGAGAA
AGCTCCCGCTTGCTGTAGCTGGAACGATGTCTTTCAGTATGAGACAAACAAAGTCACCCGGATCCAGAGCGTGAA
TTACGGCACCATCAAGTGGATCTTGCACATGACAGTCTTTTCCTACGTTAGCTTTGCTTTGATGAGCGACAAGCT
ATATCAGCGGAAGGAGCCCCTTATCAGCTCTGTGCACACCAAGGTCAAAGGCGTTGCAGAGGTGACAGAGAATGT
CACGGAGGGCGGGGTGACGAAGTTAGTACACGGCATCTTCGACACGGCCGACTACACCCTCCCTTTGCAGGGGAA
CTCGTTCTTTGTAATGACAAATTATCTCAAGTCAGAAGGCCAAGAACAGAAGCTGTGTCCTGAGTATCCCAGCCG
CGGTAAACAGTGCCATTCTGACCAGGGTTGTATAAAAGGATGGATGGACCCACAAAGTAAAGGAATCCAGACCGG
CAGGTGTATACCTTACGACCAGAAGAGGAAGACCTGTGAAATCTTTGCCTGGTGTCCTGCTGAGGAAGGGAAAGA
AGCCCCACGGCCTGCACTCTTGAGGAGCGCCGAAAACTTCACCGTACTCATCAAGAACAATATCGACTTCCCGGG
CCACAACTATACTACGAGAAACATCTTACCAGGTATGAACATCTCTTGTACCTTTCACAAGACTTGGAACCCTCA
GTGTCCCATCTTCCGGCTAGGGGACATCTTCCAGGAAATCGGAGAGAACTTTACAGAGGTGGCAGTTCAGGGAGG
AATCATGGGCATTGAGATCTACTGGGACTGCAACCTGGACAGCTGGTCCCATCGCTGTCAACCCAAATACAGCTT
CCGCCGGCTGGACGACAAGTACACCAATGAGTCCCTGTTCCCTGGCTACAACTTCAGATACGCCAAGTACTATAA
GGAAAATGGCATGGAAAAGCGGACATTGATCAAAGCCTTCGGCGTGCGTTTTGACATCCTGGTTTTTGGCACTGG
AGGAAAGTTTGACATCATCCAGTTGGTTGTGTACATTGGATCCACCCTGTCCTATTTCGGTTTGGCCACCGTGTG
TATTGACTTGATCATCAACACGTATGCCAGTACCTGCTGCAGGTCACGTGTTTACCCCTCCTGTAAGTGCTGCGA
GCCCTGTGCAGTGAATGAGTACTACTACAGAAAGAAGTGTGAGCCCATCGTGGAGCCCAAGCCGACGTTAAAGTA
TGTGTCCTTTGTGGACGAGCCCCACATTTGGATGGTGGACCAGCAGCTGCTTGGGAAAAGTCTGCAAGATGTCAA
AGGTCAAGAGGTCCCGAGACCCCAGACGGACTTCTTGGAACTGTCTAGGCTCTCCCTCTCTCTCCACCACTCACC
CCCAATTCCTGGACAACCTGAGGAAATGCAGCTGCTCCAGATAGAAGCGGTTCCTAGGTCCAGGGACAGCCCAGA
TTGGTGCCAGTGTGGAAACTGCCTCCCGTCTCAACTACCAGAGAACCGCAGGGCCCTGGAGGAGCTGTGCTGCCG
GAGGAAGCCAGGACAGTGCATCACTACCTCTGAGCTCTTCAGTAAGATCGTGCTATCCAGAGAGGCCCTGCAGCT
CCTCCTGCTCTACCAGGAGCCCTTGCTGGCGCTGGAGGGAGAGGCCATCAACAGCAAGCTGCGACACTGTGCGTA

CAGGAGCTATGCCACCTGGCGCTTTGTCTCCCAAGACATGGCCGACTTTGCCATTCTGCCCAGCTGCTGCCGCTG
GAAGATCCGGAAGGAGTTCCCCAAGACCCAGGGGCAGTACTCTGGCTTCAAGTATCCCTACGGTGGATCTGGTGG
CGGTGGATCAGGAGGCGGTGGATCAGGACTCATGGGGAGCGCTTGGAGCCACCCGCAGTTCGAAAAAGGTGGAGG
TTCTGGCGGTGGATCGGGAGGTTCAGCGTGGAGCCACCCGCAGTTCGAGAAAGCTCCCGCTTGCTGTAGCTGGAA
CGATGTCTTTCAGTATGAGACAAACAAAGTCACCCGGATCCAGAGCGTGAATTACGGCACCATCAAGTGGATCTT
GCACATGACAGTCTTTTCCTACGTTAGCTTTGCTTTGATGAGCGACAAGCTATATCAGCGGAAGGAGCCCCTTAT
CAGCTCTGTGCACACCAAGGTCAAAGGCGTTGCAGAGGTGACAGAGAATGTCACGGAGGGCGGGGTGACGAAGTT
AGTACACGGCATCTTCGACACGGCCGACTACACCCTCCCTTTGCAGGGGAACTCGTTCTTTGTAATGACAAATTA
TCTCAAGTCAGAAGGCCAAGAACAGAAGCTGTGTCCTGAGTATCCCAGCCGCGGTAAACAGTGCCATTCTGACCA
GGGTTGTATAAAAGGATGGATGGACCCACAAAGTAAAGGAATCCAGACCGGCAGGTGTATACCTTACGACCAGAA
GAGGAAGACCTGTGAAATCTTTGCCTGGTGTCCTGCTGAGGAAGGGAAAGAAGCCCCACGGCCTGCACTCTTGAG
GAGCGCCGAAAACTTCACCGTACTCATCAAGAACAATATCGACTTCCCGGGCCACAACTATACTACGAGAAACAT
CTTACCAGGTATGAACATCTCTTGTACCTTTCACAAGACTTGGAACCCTCAGTGTCCCATCTTCCGGCTAGGGGA
CATCTTCCAGGAAATCGGAGAGAACTTTACAGAGGTGGCAGTTCAGGGAGGAATCATGGGCATTGAGATCTACTG
GGACTGCAACCTGGACAGCTGGTCCCATCGCTGTCAACCCAAATACAGCTTCCGCCGGCTGGACGACAAGTACAC
CAATGAGTCCCTGTTCCCTGGCTACAACTTCAGATACGCCAAGTACTATAAGGAAAATGGCATGGAAAAGCGGAC
ATTGATCAAAGCCTTCGGCGTGCGTTTTGACATCCTGGTTTTTGGCACTGGAGGAAAGTTTGACATCATCCAGTT
GGTTGTGTACATTGGATCCACCCTGTCCTATTTCGGTTTGGCCACCGTGTGTATTGACTTGATCATCAACACGTA
TGCCAGTACCTGCTGCAGGTCACGTGTTTACCCCTCCTGTAAGTGCTGCGAGCCCTGTGCAGTGAATGAGTACTA
CTACAGAAAGAAGTGTGAGCCCATCGTGGAGCCCAAGCCGACGTTAAAGTATGTGTCCTTTGTGGACGAGCCCCA
CATTTGGATGGTGGACCAGCAGCTGCTTGGGAAAAGTCTGCAAGATGTCAAAGGTCAAGAGGTCCCGAGACCCCA
GACGGACTTCTTGGAACTGTCTAGGCTCTCCCTCTCTCTCCACCACTCACCCCCAATTCCTGGACAACCTGAGGA
AATGCAGCTGCTCCAGATAGAAGCGGTTCCTAGGTCCAGGGACAGCCCAGATTGGTGCCAGTGTGGAAACTGCCT
CCCGTCTCAACTACCAGAGAACCGCAGGGCCCTGGAGGAGCTGTGCTGCCGGAGGAAGCCAGGACAGTGCATCAC
TACCTCTGAGCTCTTCAGTAAGATCGTGCTATCCAGAGAGGCCCTGCAGCTCCTCCTGCTCTACCAGGAGCCCTT
GCTGGCGCTGGAGGGAGAGGCCATCAACAGCAAGCTGCGACACTGTGCGTACAGGAGCTATGCCACCTGGCGCTT
TGTCTCCCAAGACATGGCCGACTTTGCCATTCTGCCCAGCTGCTGCCGCTGGAAGATCCGGAAGGAGTTCCCCAA
GACCCAGGGGCAGTACTCTGGCTTCAAGTATCCCTACTGACTATCTAGAGGATCCCCGGGTACGTACCGGGCCCC
CCATCGAGGTCGACGTAGCTTTCATTTGTTTT**AA**TTT**A**TTTTTT**AAA**T**A**GC**A**TT**A**C**AAAA**C**AAA**TT**A**CC**A**C**AA**C**A**
AAAAAAAAACC**AAAA**C**AAAAA**C**AAA**T**AA**T**AAA**GTCCC**AAAA**C**AAAAA**CGG**AA**T**A**TGC**AAA**C**AAAAAAAAAAAAAA**
AAAAAAAAAAAAAAAAAAAAAAAAAAAAAAAAAAAAAGAATTGCTCGAGCGGCCGCTCGAGCAATTCCGGTCTCC
CTATAGTGAGTCGTATTACTGGCGTAATAGCGAAGAGGCCCGCACCGATCGCCCTTCCCAACAGTTGCGTAGCCT
GAATGGCGAATGGGACGCGCCCTGTAGCGGCGCATTAAGCGCGGCGGGTGTGGTGGTTACGCGCAGCGTGACCGC
TACACTTGCCAGCGCCCTAGCGCCCGCTCCTTTCGCTTTCTTCCCTTCCTTTCTCGCCACGTTCGCCGGCTTTCC
CCGTCAAGCTCTAAATCGGGGGCTCCCTTTAGGGTTCCGATTTAGTGCTTTACGGCACCTCGACCCCAAAAAACT
TGATTAGGGTGATGGTTCACGTGGGCCATCGCCCTGATAGACGGTTTTTCGCCCTTTGACGTTGGAGTCCACGTT
CTTTAATAGTGGACTCTTGTTCCAAACTGGAACAACACTCAACCCTATCTCGGTCTATTCTTTTGATTTATAAGG
GATTTTGCCGATTTCGGCCTATTGGTTAAAAAATGAGCTGATTTAACAAAAATTTAACGCGAATTTTAACAAAATA
TTAACGTTTACAATTTCAGGTGGCACTTTTCGGGGAAATGTGCGCGGAACCCCTATTTGTTTATTTTTCTAAATA
CATTCAAATATGTATCCGCTCATGAGACAATAACCCTGATAAATGCTTCAATAATATTGAAAAAGGAAGAGTATG
AGTATTCAACATTTCCGTGTCGCCCTTATTCCCTTTTTTGCGGCATTTTGCCTTCCTGTTTTTGCTCACCCAGAA
ACGCTGGTGAAAGTAAAAGATGCTGAAGATCAGTTGGGTGCACGAGTGGGTTACATCGAACTGGATCTCAACAGC
GGTAAGATCCTTGAGAGTTTTCGCCCCGAAGAACGTTTTCCAATGATGAGCACTTTTAAAGTTCTGCTATGTGGC
GCGGTATTATCCCGTATTGACGCCGGGCAAGAGCAACTCGGTCGCCGCATACACTATTCTCAGAATGACTTGGTT
GAGTACTCACCAGTCACAGAAAAGCATCTTACGGATGGCATGACAGTAAGAGAATTATGCAGTGCTGCCATAACC
ATGAGTGATAACACTGCGGCCAACTTACTTCTGACAACGATCGGAGGACCGAAGGAGCTAACCGCTTTTTTGCAC
AACATGGGGGATCATGTAACTCGCCTTGATCGTTGGGAACCGGAGCTGAATGAAGCCATACCAAACGACGAGCGT
GACACCACGATGCCTGTAGCAATGGCAACAACGTTGCGCAAACTATTAACTGGCGAACTACTTACTCTAGCTTCC
CGGCAACAATTAATAGACTGGATGGAGGCGGATAAAGTTGCAGGACCACTTCTGCGCTCGGCCCTTCCGGCTGGC
TGGTTTATTGCTGATAAATCTGGAGCCGGTGAGCGTGGGTCTCGCGGTATCATTGCAGCACTGGGGCCAGATGGT
AAGCCCTCCCGTATCGTAGTTATCTACACGACGGGGAGTCAGGCAACTATGGATGAACGAAATAGACAGATCGCT
GAGATAGGTGCCTCACTGATTAAGCATTGGTAACTGTCAGACCAAGTTTACTCATATATACTTTAGATTGATTTA
AAACTTCATTTTTAATTTAAAAGGATCTAGGTGAAGATCCTTTTTGATAATCTCATGACCAAAATCCCTTAACGT
GAGTTTTCGTTCCACTGAGCGTCAGACCCCGTAGAAAAGATCAAAGGATCTTCTTGAGATCCTTTTTTTCTGCGC
GTAATCTGCTGCTTGCAAACAAAAAAACCACCGCTACCAGCGGTGGTTTGTTTGCCGGATCAAGAGCTACCAACT
CTTTTTCCGAAGGTAACTGGCTTCAGCAGAGCGCAGATACCAAATACTGTCCTTCTAGTGTAGCCGTAGTTAGGC
CACCACTTCAAGAACTCTGTAGCACCGCCTACATACCTCGCTCTGCTAATCCTGTTACCAGTGGCTGCTGCCAGT
GGCGATAAGTCGTGTCTTACCGGGTTGGACTCAAGACGATAGTTACCGGATAAGGCGCAGCGGTCGGGCTGAACG

GGGGGTTCGTGCACACAGCCCAGCTTGGAGCGAACGACCTACACCGAACTGAGATACCTACAGCGTGAGCATTGA
GAAAGCGCCACGCTTCCCGAAGGGAGAAAGGCGGACAGGTATCCGGTAAGCGGCAGGGTCGGAACAGGAGAGCGC
ACGAGGGAGCTTCCAGGGGGAAACGCCTGGTATCTTTATAGTCCTGTCGGGTTTCGCCACCTCTGACTTGAGCGT
CGATTTTTGTGATGCTCGTCAGGGGGGCGGAGCCTATGGAAAAACGCCAGCAACGCGGCCTTTTTACGGTTCCTG
GCCTTTTGCTGGCCTTTTGCTCACATGTTCTTTCCTGCGTTATCCCCTGATTCTGTGGATAACCGTATTACCGCC
TTTGAGTGAGCTGATACCGCTCGCCGCAGCCGAACGACCGAGCGCAGCGAGTCAGTGAGCGAGGAAGCGGAAGAG
CGCCCAATACGCAAACCGCCTCTCCCCGCGCGTTGGCCGATTCATTAATGCAGTAATACATAACCTTATGTATCA
TACACATACGATTTAGGTGACACTATAG

>K-9254 ^{S3K64A}rP2X7^{S3K64A}rP2X7^{S3}rP2X7-pNKS4 ($^{ko-ko-wt}$trimer) 216.494 Da, pI 8.29, 1.911 residues

AATACAAGCTTACAACAAAGAACAACAAACAACAAAGTCCGACGTCGAAGTAGCCACCTACCATCATCATCATCA
TCCCATGGGGAGCGCTTGGAGCCACCCGCAGTTCGAAAAAGGTGGAGGTTCTGGCGGTGGATCGGGAGGTTCAGC
GTGGAGCCACCCGCAGTTCGAGAAAGCTCCCGCTTGCTGTAGCTGGAACGATGTCTTTCAGTATGAGACAAACAA
AGTCACCCGGATCCAGAGCGTGAATTACGGCACCATCAAGTGGATCTTGCACATGACAGTCTTTTCCTACGTTAG
CTTTGCTTTGATGAGCGACAAGCTATATCAGCGGAAGGAGCCCCTTATCAGCTCTGTGCACACCGCTGTCAAAGG
CGTTGCAGAGGTGACAGAGAATGTCACGGAGGGCGGGGTGACGAAGTTAGTACACGGCATCTTCGACACGGCCGA
CTACACCCTCCCTTTGCAGGGGAACTCGTTCTTTGTAATGACAAATTATCTCAAGTCAGAAGGCCAAGAACAGAA
GCTGTGTCCTGAGTATCCCAGCCGCGGTAAACAGTGCCATTCTGACCAGGGTTGTATAAAAGGATGGATGGACCC
ACAAAGTAAAGGAATCCAGACCGGCAGGTGTATACCTTACGACCAGAAGAGGAAGACCTGTGAAATCTTTGCCTG
GTGTCCTGCTGAGGAAGGGAAAGAAGCCCCACGGCCTGCACTCTTGAGGAGCGCCGAAAACTTCACCGTACTCAT
CAAGAACAATATCGACTTCCCGGGCCACAACTATACTACGAGAAACATCTTACCAGGTATGAACATCTCTTGTAC
CTTTCACAAGACTTGGAACCCTCAGTGTCCCATCTTCCGGCTAGGGGACATCTTCCAGGAAATCGGAGAGAACTT
TACAGAGGTGGCAGTTCAGGGAGGAATCATGGGCATTGAGATCTACTGGGACTGCAACCTGGACAGCTGGTCCCA
TCGCTGTCAACCCAAATACAGCTTCCGCCGGCTGGACGACAAGTACACCAATGAGTCCCTGTTCCCTGGCTACAA
CTTCAGATACGCCAAGTACTATAAGGAAAATGGCATGGAAAAGCGGACATTGATCAAAGCCTTCGGCGTGCGTTT
TGACATCCTGGTTTTTGGCACTGGAGGAAAGTTTGACATCATCCAGTTGGTTGTGTACATTGGATCCACCCTGTC
CTATTTCGGTTTGGCCACCGTGTGTATTGACTTGATCATCAACACGTATGCCAGTACCTGCTGCAGGTCACGTGT
TTACCCCTCCTGTAAGTGCTGCGAGCCCTGTGCAGTGAATGAGTACTACTACAGAAAGAAGTGTGAGCCCATCGT
GGAGCCCAAGCCGACGTTAAAGTATGTGTCCTTTGTGGACGAGCCCCACATTTGGATGGTGGACCAGCAGCTGCT
TGGGAAAAGTCTGCAAGATGTCAAAGGTCAAGAGGTCCCGAGACCCCAGACGGACTTCTTGGAACTGTCTAGGCT
CTCCCTCTCTCTCCACCACTCACCCCCAATTCCTGGACAACCTGAGGAAATGCAGCTGCTCCAGATAGAAGCGGT
TCCTAGGTCCAGGGACAGCCCAGATTGGTGCCAGTGTGGAAACTGCCTCCCGTCTCAACTACCAGAGAACCGCAG
GGCCCTGGAGGAGCTGTGCTGCCGGAGGAAGCCAGGACAGTGCATCACTACCTCTGAGCTCTTCAGTAAGATCGT
GCTATCCAGAGAGGCCCTGCAGCTCCTCCTGCTCTACCAGGAGCCCTTGCTGGCGCTGGAGGGAGAGGCCATCAA
CAGCAAGCTGCGACACTGTGCGTACAGGAGCTATGCCACCTGGCGCTTTGTCTCCCAAGACATGGCCGACTTTGC
CATTCTGCCCAGCTGCTGCCGCTGGAAGATCCGGAAGGAGTTCCCCAAGACCCAGGGGCAGTACTCTGGCTTCAA
GTATCCCTACGGTGGATCTGGTGGCGGTGGATCAGGAGGCGGTGGATCAGGACTCATGGGGAGCGCTTGGAGCCA
CCCGCAGTTCGAAAAAGGTGGAGGTTCTGGCGGTGGATCGGGAGGTTCAGCGTGGAGCCACCCGCAGTTCGAGAA
AGCTCCCGCTTGCTGTAGCTGGAACGATGTCTTTCAGTATGAGACAAACAAAGTCACCCGGATCCAGAGCGTGAA
TTACGGCACCATCAAGTGGATCTTGCACATGACAGTCTTTTCCTACGTTAGCTTTGCTTTGATGAGCGACAAGCT
ATATCAGCGGAAGGAGCCCCTTATCAGCTCTGTGCACACCGCTGTCAAAGGCGTTGCAGAGGTGACAGAGAATGT
CACGGAGGGCGGGGTGACGAAGTTAGTACACGGCATCTTCGACACGGCCGACTACACCCTCCCTTTGCAGGGGAA
CTCGTTCTTTGTAATGACAAATTATCTCAAGTCAGAAGGCCAAGAACAGAAGCTGTGTCCTGAGTATCCCAGCCG
CGGTAAACAGTGCCATTCTGACCAGGGTTGTATAAAAGGATGGATGGACCCACAAAGTAAAGGAATCCAGACCGG
CAGGTGTATACCTTACGACCAGAAGAGGAAGACCTGTGAAATCTTTGCCTGGTGTCCTGCTGAGGAAGGGAAAGA
AGCCCCACGGCCTGCACTCTTGAGGAGCGCCGAAAACTTCACCGTACTCATCAAGAACAATATCGACTTCCCGGG
CCACAACTATACTACGAGAAACATCTTACCAGGTATGAACATCTCTTGTACCTTTCACAAGACTTGGAACCCTCA
GTGTCCCATCTTCCGGCTAGGGGACATCTTCCAGGAAATCGGAGAGAACTTTACAGAGGTGGCAGTTCAGGGAGG
AATCATGGGCATTGAGATCTACTGGGACTGCAACCTGGACAGCTGGTCCCATCGCTGTCAACCCAAATACAGCTT
CCGCCGGCTGGACGACAAGTACACCAATGAGTCCCTGTTCCCTGGCTACAACTTCAGATACGCCAAGTACTATAA
GGAAAATGGCATGGAAAAGCGGACATTGATCAAAGCCTTCGGCGTGCGTTTTGACATCCTGGTTTTTGGCACTGG
AGGAAAGTTTGACATCATCCAGTTGGTTGTGTACATTGGATCCACCCTGTCCTATTTCGGTTTGGCCACCGTGTG
TATTGACTTGATCATCAACACGTATGCCAGTACCTGCTGCAGGTCACGTGTTTACCCCTCCTGTAAGTGCTGCGA
GCCCTGTGCAGTGAATGAGTACTACTACAGAAAGAAGTGTGAGCCCATCGTGGAGCCCAAGCCGACGTTAAAGTA

TGTGTCCTTTGTGGACGAGCCCCACATTTGGATGGTGGACCAGCAGCTGCTTGGGAAAAGTCTGCAAGATGTCAA
AGGTCAAGAGGTCCCGAGACCCCAGACGGACTTCTTGGAACTGTCTAGGCTCTCCCTCTCTCTCCACCACTCACC
CCCAATTCCTGGACAACCTGAGGAAATGCAGCTGCTCCAGATAGAAGCGGTTCCTAGGTCCAGGGACAGCCCAGA
TTGGTGCCAGTGTGGAAACTGCCTCCCGTCTCAACTACCAGAGAACCGCAGGGCCCTGGAGGAGCTGTGCTGCCG
GAGGAAGCCAGGACAGTGCATCACTACCTCTGAGCTCTTCAGTAAGATCGTGCTATCCAGAGAGGCCCTGCAGCT
CCTCCTGCTCTACCAGGAGCCCTTGCTGGCGCTGGAGGGAGAGGCCATCAACAGCAAGCTGCGACACTGTGCGTA
CAGGAGCTATGCCACCTGGCGCTTTGTCTCCCAAGACATGGCCGACTTTGCCATTCTGCCCAGCTGCTGCCGCTG
GAAGATCCGGAAGGAGTTCCCCAAGACCCAGGGGCAGTACTCTGGCTTCAAGTATCCCTACGGTGGATCTGGTGG
CGGTGGATCAGGAGGCGGTGGATCAGGACTCATGGGAGCGCTTGGAGCCACCCGCAGTTCGAAAAAGGTGGAGG
TTCTGGCGGTGGATCGGGAGGTTCAGCGTGGAGCCACCCGCAGTTCGAGAAAGCTCCCGCTTGCTGTAGCTGGAA
CGATGTCTTTCAGTATGAGACAAACAAAGTCACCCGGATCCAGAGCGTGAATTACGGCACCATCAAGTGGATCTT
GCACATGACAGTCTTTTCCTACGTTAGCTTTGCTTTGATGAGCGACAAGCTATATCAGCGGAAGGAGCCCCTTAT
CAGCTCTGTGCACACCAAGGTCAAAGGCGTTGCAGAGGTGACAGAGAATGTCACGGAGGGCGGGGTGACGAAGTT
AGTACACGGCATCTTCGACACGGCCGACTACACCCTCCCTTTGCAGGGGAACTCGTTCTTTGTAATGACAAATTA
TCTCAAGTCAGAAGGCCAAGAACAGAAGCTGTGTCCTGAGTATCCCAGCCGCGGTAAACAGTGCCATTCTGACCA
GGGTTGTATAAAAGGATGGATGGACCCACAAAGTAAAGGAATCCAGACCGGCAGGTGTATACCTTACGACCAGAA
GAGGAAGACCTGTGAAATCTTTGCCTGGTGTCCTGCTGAGGAAGGGAAAGAAGCCCCACGGCCTGCACTCTTGAG
GAGCGCCGAAAACTTCACCGTACTCATCAAGAACAATATCGACTTCCCGGGCCACAACTATACTACGAGAAACAT
CTTACCAGGTATGAACATCTCTTGTACCTTTCACAAGACTTGGAACCCTCAGTGTCCCATCTTCCGGCTAGGGGA
CATCTTCCAGGAAATCGGAGAGAACTTTACAGAGGTGGCAGTTCAGGGAGGAATCATGGGCATTGAGATCTACTG
GGACTGCAACCTGGACAGCTGGTCCCATCGCTGTCAACCCAAATACAGCTTCCGCCGGCTGGACGACAAGTACAC
CAATGAGTCCCTGTTCCCTGGCTACAACTTCAGATACGCCAAGTACTATAAGGAAAATGGCATGGAAAAGCGGAC
ATTGATCAAAGCCTTCGGCGTGCGTTTTGACATCCTGGTTTTTGGCACTGGAGGAAAGTTTGACATCATCCAGTT
GGTTGTGTACATTGGATCCACCCTGTCCTATTTCGGTTTGGCCACCGTGTGTATTGACTTGATCATCAACACGTA
TGCCAGTACCTGCTGCAGGTCACGTGTTTACCCCTCCTGTAAGTGCTGCGAGCCCTGTGCAGTGAATGAGTACTA
CTACAGAAAGAAGTGTGAGCCCATCGTGGAGCCCAAGCCGACGTTAAAGTATGTGTCCTTTGTGGACGAGCCCCA
CATTTGGATGGTGGACCAGCAGCTGCTTGGGAAAAGTCTGCAAGATGTCAAAGGTCAAGAGGTCCCGAGACCCCA
GACGGACTTCTTGGAACTGTCTAGGCTCTCCCTCTCTCTCCACCACTCACCCCCAATTCCTGGACAACCTGAGGA
AATGCAGCTGCTCCAGATAGAAGCGGTTCCTAGGTCCAGGGACAGCCCAGATTGGTGCCAGTGTGGAAACTGCCT
CCCGTCTCAACTACCAGAGAACCGCAGGGCCCTGGAGGAGCTGTGCTGCCGGAGGAAGCCAGGACAGTGCATCAC
TACCTCTGAGCTCTTCAGTAAGATCGTGCTATCCAGAGAGGCCCTGCAGCTCCTCCTGCTCTACCAGGAGCCCTT
GCTGGCGCTGGAGGGAGAGGCCATCAACAGCAAGCTGCGACACTGTGCGTACAGGAGCTATGCCACCTGGCGCTT
TGTCTCCCAAGACATGGCCGACTTTGCCATTCTGCCCAGCTGCTGCCGCTGGAAGATCCGGAAGGAGTTCCCCAA
GACCCAGGGGCAGTACTCTGGCTTCAAGTATCCCTACTGACTATCTAGAGGATCCCCGGGTACGTACCGGGCCCC
CCATCGAGGTCGACGTAGCTTTCATTTGTTTT**AA**TTT**A**TTTTTT**AAA**T**A**GC**A**TT**A**C**AAAA**C**AAA**TT**A**CC**A**C**AA**C**A**
AAAAAAAAACC**AAAA**C**AAAAA**C**AAA**T**AA**T**AAA**GTCCC**AAAA**C**AAAAA**CGG**AA**T**A**TGC**AAA**C**AAAAAAAAAAAAAA**
AAAAAAAAAAAAAAAAAAAAAAAAAAAAAAAAAAAAAGAATTGCTCGAGCGGCCGCTCGAGCAATTCCGGTCTCC
CTATAGTGAGTCGTATTACTGGCGTAATAGCGAAGAGGCCCGCACCGATCGCCCTTCCCAACAGTTGCGTAGCCT
GAATGGCGAATGGGACGCGCCCTGTAGCGGCGCATTAAGCGCGGCGGGTGTGGTGGTTACGCGCAGCGTGACCGC
TACACTTGCCAGCGCCCTAGCGCCCGCTCCTTTCGCTTTCTTCCCTTCCTTTCTCGCCACGTTCGCCGGCTTTCC
CCGTCAAGCTCTAAATCGGGGGCTCCCTTTAGGGTTCCGATTTAGTGCTTTACGGCACCTCGACCCCAAAAAACT
TGATTAGGGTGATGGTTCACGTGGGCCATCGCCCTGATAGACGGTTTTTCGCCCTTTGACGTTGGAGTCCACGTT
CTTTAATAGTGGACTCTTGTTCCAAACTGGAACAACACTCAACCCTATCTCGGTCTATTCTTTTGATTTATAAGG
GATTTTGCCGATTTCGGCCTATTGGTTAAAAAATGAGCTGATTTAACAAAAATTTAACGCGAATTTTAACAAAATA
TTAACGTTTACAATTTCAGGTGGCACTTTTCGGGGAAATGTGCGCGGAACCCCTATTTGTTTATTTTTCTAAATA
CATTCAAATATGTATCCGCTCATGAGACAATAACCCTGATAAATGCTTCAATAATATTGAAAAAGGAAGAGTATG
AGTATTCAACATTTCCGTGTCGCCCTTATTCCCTTTTTTGCGGCATTTTGCCTTCCTGTTTTTGCTCACCCAGAA
ACGCTGGTGAAAGTAAAAGATGCTGAAGATCAGTTGGGTGCACGAGTGGGTTACATCGAACTGGATCTCAACAGC
GGTAAGATCCTTGAGAGTTTTCGCCCCGAAGAACGTTTTCCAATGATGAGCACTTTTAAAGTTCTGCTATGTGGC
GCGGTATTATCCCGTATTGACGCCGGGCAAGAGCAACTCGGTCGCCGCATACACTATTCTCAGAATGACTTGGTT
GAGTACTCACCAGTCACAGAAAAGCATCTTACGGATGGCATGACAGTAAGAGAATTATGCAGTGCTGCCATAACC
ATGAGTGATAACACTGCGGCCAACTTACTTCTGACAACGATCGGAGGACCGAAGGAGCTAACCGCTTTTTTGCAC
AACATGGGGGATCATGTAACTCGCCTTGATCGTTGGGAACCGGAGCTGAATGAAGCCATACCAAACGACGAGCGT
GACACCACGATGCCTGTAGCAATGGCAACAACGTTGCGCAAACTATTAACTGGCGAACTACTTACTCTAGCTTCC
CGGCAACAATTAATAGACTGGATGGAGGCGGATAAAGTTGCAGGACCACTTCTGCGCTCGGCCCTTCCGGCTGGC
TGGTTTATTGCTGATAAATCTGGAGCCGGTGAGCGTGGGTCTCGCGGTATCATTGCAGCACTGGGGCCAGATGGT
AAGCCCTCCCGTATCGTAGTTATCTACACGACGGGGAGTCAGGCAACTATGGATGAACGAAATAGACAGATCGCT
GAGATAGGTGCCTCACTGATTAAGCATTGGTAACTGTCAGACCAAGTTTACTCATATATACTTTAGATTGATTTA

AAACTTCATTTTTAATTTAAAAGGATCTAGGTGAAGATCCTTTTTGATAATCTCATGACCAAAATCCCTTAACGT
GAGTTTTCGTTCCACTGAGCGTCAGACCCCGTAGAAAAGATCAAAGGATCTTCTTGAGATCCTTTTTTTCTGCGC
GTAATCTGCTGCTTGCAAACAAAAAAACCACCGCTACCAGCGGTGGTTTGTTTGCCGGATCAAGAGCTACCAACT
CTTTTTCCGAAGGTAACTGGCTTCAGCAGAGCGCAGATACCAAATACTGTCCTTCTAGTGTAGCCGTAGTTAGGC
CACCACTTCAAGAACTCTGTAGCACCGCCTACATACCTCGCTCTGCTAATCCTGTTACCAGTGGCTGCTGCCAGT
GGCGATAAGTCGTGTCTTACCGGGTTGGACTCAAGACGATAGTTACCGGATAAGGCGCAGCGGTCGGGCTGAACG
GGGGGTTCGTGCACACAGCCCAGCTTGGAGCGAACGACCTACACCGAACTGAGATACCTACAGCGTGAGCATTGA
GAAAGCGCCACGCTTCCCGAAGGGAGAAAGGCGGACAGGTATCCGGTAAGCGGCAGGGTCGGAACAGGAGAGCGC
ACGAGGGAGCTTCCAGGGGGAAACGCCTGGTATCTTTATAGTCCTGTCGGGTTTCGCCACCTCTGACTTGAGCGT
CGATTTTTGTGATGCTCGTCAGGGGGGCGGAGCCTATGGAAAAACGCCAGCAACGCGGCCTTTTTACGGTTCCTG
GCCTTTTGCTGGCCTTTTGCTCACATGTTCTTTCCTGCGTTATCCCCTGATTCTGTGGATAACCGTATTACCGCC
TTTGAGTGAGCTGATACCGCTCGCCGCAGCCGAACGACCGAGCGCAGCGAGTCAGTGAGCGAGGAAGCGGAAGAG
CGCCCAATACGCAAACCGCCTCTCCCCGCGCGTTGGCCGATTCATTAATGCAGTAATACATAACCTTATGTATCA
TACACATACGATTTAGGTGACACTATAG

>K-9326 ^{S3}rP2X7^{S3K64A}rP2X7^{S3K64A}rP2X7-pNKS4 ($^{wt-ko-ko}$trimer) 216.494 Da, pI 8.29, 1.911 residues

AATACAAGCTTACAACAAAGAACAACAAACAACAAAGTCCGACGTCGAAGTAGCCACCTACCATCATCATCATCA
TCCCATGGGGAGCGCTTGGAGCCACCCGCAGTTCGAAAAAGGTGGAGGTTCTGGCGGTGGATCGGGAGGTTCAGC
GTGGAGCCACCCGCAGTTCGAGAAAGCTCCCGCTTGCTGTAGCTGGAACGATGTCTTTCAGTATGAGACAAACAA
AGTCACCCGGATCCAGAGCGTGAATTACGGCACCATCAAGTGGATCTTGCACATGACAGTCTTTTCCTACGTTAG
CTTTGCTTTGATGAGCGACAAGCTATATCAGCGGAAGGAGCCCCTTATCAGCTCTGTGCACACCAAGGTCAAAGG
CGTTGCAGAGGTGACAGAGAATGTCACGGAGGGCGGGGTGACGAAGTTAGTACACGGCATCTTCGACACGGCCGA
CTACACCCTCCCTTTGCAGGGGAACTCGTTCTTTGTAATGACAAATTATCTCAAGTCAGAAGGCCAAGAACAGAA
GCTGTGTCCTGAGTATCCCAGCCGCGGTAAACAGTGCCATTCTGACCAGGGTTGTATAAAAGGATGGATGGACCC
ACAAAGTAAAGGAATCCAGACCGGCAGGTGTATACCTTACGACCAGAAGAGGAAGACCTGTGAAATCTTTGCCTG
GTGTCCTGCTGAGGAAGGGAAAGAAGCCCCACGGCCTGCACTCTTGAGGAGCGCCGAAAACTTCACCGTACTCAT
CAAGAACAATATCGACTTCCCGGGCCACAACTATACTACGAGAAACATCTTACCAGGTATGAACATCTCTTGTAC
CTTTCACAAGACTTGGAACCCTCAGTGTCCCATCTTCCGGCTAGGGGACATCTTCCAGGAAATCGGAGAGAACTT
TACAGAGGTGGCAGTTCAGGGAGGAATCATGGGCATTGAGATCTACTGGGACTGCAACCTGGACAGCTGGTCCCA
TCGCTGTCAACCCAAATACAGCTTCCGCCGGCTGGACGACAAGTACACCAATGAGTCCCTGTTCCCTGGCTACAA
CTTCAGATACGCCAAGTACTATAAGGAAAATGGCATGGAAAAGCGGACATTGATCAAAGCCTTCGGCGTGCGTTT
TGACATCCTGGTTTTTGGCACTGGAGGAAAGTTTGACATCATCCAGTTGGTTGTGTACATTGGATCCACCCTGTC
CTATTTCGGTTTGGCCACCGTGTGTATTGACTTGATCATCAACACGTATGCCAGTACCTGCTGCAGGTCACGTGT
TTACCCCTCCTGTAAGTGCTGCGAGCCCTGTGCAGTGAATGAGTACTACTACAGAAAGAAGTGTGAGCCCATCGT
GGAGCCCAAGCCGACGTTAAAGTATGTGTCCTTTGTGGACGAGCCCCACATTTGGATGGTGGACCAGCAGCTGCT
TGGGAAAAGTCTGCAAGATGTCAAAGGTCAAGAGGTCCCGAGACCCCAGACGGACTTCTTGGAACTGTCTAGGCT
CTCCCTCTCTCTCCACCACTCACCCCCAATTCCTGGACAACCTGAGGAAATGCAGCTGCTCCAGATAGAAGCGGT
TCCTAGGTCCAGGGACAGCCCAGATTGGTGCCAGTGTGGAAACTGCCTCCCGTCTCAACTACCAGAGAACCGCAG
GGCCCTGGAGGAGCTGTGCTGCCGGAGGAAGCCAGGACAGTGCATCACTACCTCTGAGCTCTTCAGTAAGATCGT
GCTATCCAGAGAGGCCCTGCAGCTCCTCCTGCTCTACCAGGAGCCCTTGCTGGCGCTGGAGGGAGAGGCCATCAA
CAGCAAGCTGCGACACTGTGCGTACAGGAGCTATGCCACCTGGCGCTTTGTCTCCCAAGACATGGCCGACTTTGC
CATTCTGCCCAGCTGCTGCCGCTGGAAGATCCGGAAGGAGTTCCCCAAGACCCAGGGGCAGTACTCTGGCTTCAA
GTATCCCTACGGTGGATCTGGTGGCGGTGGATCAGGAGGCGGTGGATCAGGACTCATGGGGAGCGCTTGGAGCCA
CCCGCAGTTCGAAAAAGGTGGAGGTTCTGGCGGTGGATCGGGAGGTTCAGCGTGGAGCCACCCGCAGTTCGAGAA
AGCTCCCGCTTGCTGTAGCTGGAACGATGTCTTTCAGTATGAGACAAACAAAGTCACCCGGATCCAGAGCGTGAA
TTACGGCACCATCAAGTGGATCTTGCACATGACAGTCTTTTCCTACGTTAGCTTTGCTTTGATGAGCGACAAGCT
ATATCAGCGGAAGGAGCCCCTTATCAGCTCTGTGCACACCGCTGTCAAAGGCGTTGCAGAGGTGACAGAGAATGT
CACGGAGGGCGGGGTGACGAAGTTAGTACACGGCATCTTCGACACGGCCGACTACACCCTCCCTTTGCAGGGGAA
CTCGTTCTTTGTAATGACAAATTATCTCAAGTCAGAAGGCCAAGAACAGAAGCTGTGTCCTGAGTATCCCAGCCG
CGGTAAACAGTGCCATTCTGACCAGGGTTGTATAAAAGGATGGATGGACCCACAAAGTAAAGGAATCCAGACCGG
CAGGTGTATACCTTACGACCAGAAGAGGAAGACCTGTGAAATCTTTGCCTGGTGTCCTGCTGAGGAAGGGAAAGA
AGCCCCACGGCCTGCACTCTTGAGGAGCGCCGAAAACTTCACCGTACTCATCAAGAACAATATCGACTTCCCGGG
CCACAACTATACTACGAGAAACATCTTACCAGGTATGAACATCTCTTGTACCTTTCACAAGACTTGGAACCCTCA
GTGTCCCATCTTCCGGCTAGGGGACATCTTCCAGGAAATCGGAGAGAACTTTACAGAGGTGGCAGTTCAGGGAGG
AATCATGGGCATTGAGATCTACTGGGACTGCAACCTGGACAGCTGGTCCCATCGCTGTCAACCCAAATACAGCTT
CCGCCGGCTGGACGACAAGTACACCAATGAGTCCCTGTTCCCTGGCTACAACTTCAGATACGCCAAGTACTATAA

GGAAAATGGCATGGAAAAGCGGACATTGATCAAAGCCTTCGGCGTGCGTTTTGACATCCTGGTTTTTGGCACTGG
AGGAAAGTTTGACATCATCCAGTTGGTTGTGTACATTGGATCCACCCTGTCCTATTTCGGTTTGGCCACCGTGTG
TATTGACTTGATCATCAACACGTATGCCAGTACCTGCTGCAGGTCACGTGTTTACCCCTCCTGTAAGTGCTGCGA
GCCCTGTGCAGTGAATGAGTACTACTACAGAAAGAAGTGTGAGCCCATCGTGGAGCCCAAGCCGACGTTAAAGTA
TGTGTCCTTTGTGGACGAGCCCCACATTTGGATGGTGGACCAGCAGCTGCTTGGGAAAAGTCTGCAAGATGTCAA
AGGTCAAGAGGTCCCGAGACCCCAGACGGACTTCTTGGAACTGTCTAGGCTCTCCCTCTCTCTCCACCACTCACC
CCCAATTCCTGGACAACCTGAGGAAATGCAGCTGCTCCAGATAGAAGCGGTTCCTAGGTCCAGGGACAGCCCAGA
TTGGTGCCAGTGTGGAAACTGCCTCCCGTCTCAACTACCAGAGAACCGCAGGGCCCTGGAGGAGCTGTGCTGCCG
GAGGAAGCCAGGACAGTGCATCACTACCTCTGAGCTCTTCAGTAAGATCGTGCTATCCAGAGAGGCCCTGCAGCT
CCTCCTGCTCTACCAGGAGCCCTTGCTGGCGCTGGAGGGAGAGGCCATCAACAGCAAGCTGCGACACTGTGCGTA
CAGGAGCTATGCCACCTGGCGCTTTGTCTCCCAAGACATGGCCGACTTTGCCATTCTGCCCAGCTGCTGCCGCTG
GAAGATCCGGAAGGAGTTCCCCAAGACCCAGGGGCAGTACTCTGGCTTCAAGTATCCCTACGGTGGATCTGGTGG
CGGTGGATCAGGAGGCGGTGGATCAGGACTCATGGGGAGCGCTTGGAGCCACCCGCAGTTCGAAAAAGGTGGAGG
TTCTGGCGGTGGATCGGGAGGTTCAGCGTGGAGCCACCCGCAGTTCGAGAAAGCTCCCGCTTGCTGTAGCTGGAA
CGATGTCTTTCAGTATGAGACAAACAAAGTCACCCGGATCCAGAGCGTGAATTACGGCACCATCAAGTGGATCTT
GCACATGACAGTCTTTTCCTACGTTAGCTTTGCTTTGATGAGCGACAAGCTATATCAGCGGAAGGAGCCCCTTAT
CAGCTCTGTGCACACCGCTGTCAAAGGCGTTGCAGAGGTGACAGAGAATGTCACGGAGGGCGGGGTGACGAAGTT
AGTACACGGCATCTTCGACACGGCCGACTACACCCTCCCTTTGCAGGGGAACTCGTTCTTTGTAATGACAAATTA
TCTCAAGTCAGAAGGCCAAGAACAGAAGCTGTGTCCTGAGTATCCCAGCCGCGGTAAACAGTGCCATTCTGACCA
GGGTTGTATAAAAGGATGGATGGACCCACAAAGTAAAGGAATCCAGACCGGCAGGTGTATACCTTACGACCAGAA
GAGGAAGACCTGTGAAATCTTTGCCTGGTGTCCTGCTGAGGAAGGGAAAGAAGCCCCACGGCCTGCACTCTTGAG
GAGCGCCGAAAACTTCACCGTACTCATCAAGAACAATATCGACTTCCCGGGCCACAACTATACTACGAGAAACAT
CTTACCAGGTATGAACATCTCTTGTACCTTTCACAAGACTTGGAACCCTCAGTGTCCCATCTTCCGGCTAGGGGA
CATCTTCCAGGAAATCGGAGAGAACTTTACAGAGGTGGCAGTTCAGGGAGGAATCATGGGCATTGAGATCTACTG
GGACTGCAACCTGGACAGCTGGTCCCATCGCTGTCAACCCAAATACAGCTTCCGCCGGCTGGACGACAAGTACAC
CAATGAGTCCCTGTTCCCTGGCTACAACTTCAGATACGCCAAGTACTATAAGGAAAATGGCATGGAAAAGCGGAC
ATTGATCAAAGCCTTCGGCGTGCGTTTTGACATCCTGGTTTTTGGCACTGGAGGAAAGTTTGACATCATCCAGTT
GGTTGTGTACATTGGATCCACCCTGTCCTATTTCGGTTTGGCCACCGTGTGTATTGACTTGATCATCAACACGTA
TGCCAGTACCTGCTGCAGGTCACGTGTTTACCCCTCCTGTAAGTGCTGCGAGCCCTGTGCAGTGAATGAGTACTA
CTACAGAAAGAAGTGTGAGCCCATCGTGGAGCCCAAGCCGACGTTAAAGTATGTGTCCTTTGTGGACGAGCCCCA
CATTTGGATGGTGGACCAGCAGCTGCTTGGGAAAAGTCTGCAAGATGTCAAAGGTCAAGAGGTCCCGAGACCCCA
GACGGACTTCTTGGAACTGTCTAGGCTCTCCCTCTCTCTCCACCACTCACCCCCAATTCCTGGACAACCTGAGGA
AATGCAGCTGCTCCAGATAGAAGCGGTTCCTAGGTCCAGGGACAGCCCAGATTGGTGCCAGTGTGGAAACTGCCT
CCCGTCTCAACTACCAGAGAACCGCAGGGCCCTGGAGGAGCTGTGCTGCCGGAGGAAGCCAGGACAGTGCATCAC
TACCTCTGAGCTCTTCAGTAAGATCGTGCTATCCAGAGAGGCCCTGCAGCTCCTCCTGCTCTACCAGGAGCCCTT
GCTGGCGCTGGAGGGAGAGGCCATCAACAGCAAGCTGCGACACTGTGCGTACAGGAGCTATGCCACCTGGCGCTT
TGTCTCCCAAGACATGGCCGACTTTGCCATTCTGCCCAGCTGCTGCCGCTGGAAGATCCGGAAGGAGTTCCCCAA
GACCCAGGGGCAGTACTCTGGCTTCAAGTATCCCTACTGACTATCTAGAGGATCCCCGGGTACGTACCGGGCCCC
CCATCGAGGTCGACGTAGCTTTCATTTGTTTT**AA**TTT**A**TTTTTT**AAA**T**A**GC**A**TT**A**C**AAAA**C**AAA**TT**A**CC**A**C**AA**C**A**
AAAAAAAAACC**AAAA**C**AAAAA**C**AAA**T**AA**T**AAA**GTCCC**AAAA**C**AAAAA**CGG**AA**T**A**TGC**AAA**C**AAAAAAAAAAAAAA**
AAAAAAAAAAAAAAAAAAAAAAAAAAAAAAAAAAAAAGAATTGCTCGAGCGGCCGCTCGAGCAATTCCGGTCTCC
CTATAGTGAGTCGTATTACTGGCGTAATAGCGAAGAGGCCCGCACCGATCGCCCTTCCCAACAGTTGCGTAGCCT
GAATGGCGAATGGGACGCGCCCTGTAGCGGCGCATTAAGCGCGGCGGGTGTGGTGGTTACGCGCAGCGTGACCGC
TACACTTGCCAGCGCCCTAGCGCCCGCTCCTTTCGCTTTCTTCCCTTCCTTTCTCGCCACGTTCGCCGGCTTTCC
CCGTCAAGCTCTAAATCGGGGGCTCCCTTTAGGGTTCCGATTTAGTGCTTTACGGCACCTCGACCCCAAAAAACT
TGATTAGGGTGATGGTTCACGTGGGCCATCGCCCTGATAGACGGTTTTTCGCCCTTTGACGTTGGAGTCCACGTT
CTTTAATAGTGGACTCTTGTTCCAAACTGGAACAACACTCAACCCTATCTCGGTCTATTCTTTTGATTTATAAGG
GATTTTGCCGATTTCGGCCTATTGGTTAAAAAATGAGCTGATTTAACAAAAATTTAACGCGAATTTTAACAAAATA
TTAACGTTTACAATTTCAGGTGGCACTTTTCGGGGAAATGTGCGCGGAACCCCTATTTGTTTATTTTTCTAAATA
CATTCAAATATGTATCCGCTCATGAGACAATAACCCTGATAAATGCTTCAATAATATTGAAAAAGGAAGAGTATG
AGTATTCAACATTTCCGTGTCGCCCTTATTCCCTTTTTTGCGGCATTTTGCCTTCCTGTTTTTGCTCACCCAGAA
ACGCTGGTGAAAGTAAAAGATGCTGAAGATCAGTTGGGTGCACGAGTGGGTTACATCGAACTGGATCTCAACAGC
GGTAAGATCCTTGAGAGTTTTCGCCCCGAAGAACGTTTTCCAATGATGAGCACTTTTAAAGTTCTGCTATGTGGC
GCGGTATTATCCCGTATTGACGCCGGGCAAGAGCAACTCGGTCGCCGCATACACTATTCTCAGAATGACTTGGTT
GAGTACTCACCAGTCACAGAAAAGCATCTTACGGATGGCATGACAGTAAGAGAATTATGCAGTGCTGCCATAACC
ATGAGTGATAACACTGCGGCCAACTTACTTCTGACAACGATCGGAGGACCGAAGGAGCTAACCGCTTTTTTGCAC
AACATGGGGGATCATGTAACTCGCCTTGATCGTTGGGAACCGGAGCTGAATGAAGCCATACCAAACGACGAGCGT
GACACCACGATGCCTGTAGCAATGGCAACAACGTTGCGCAAACTATTAACTGGCGAACTACTTACTCTAGCTTCC

CGGCAACAATTAATAGACTGGATGGAGGCGGATAAAGTTGCAGGACCACTTCTGCGCTCGGCCCTTCCGGCTGGC
TGGTTTATTGCTGATAAATCTGGAGCCGGTGAGCGTGGGTCTCGCGGTATCATTGCAGCACTGGGGCCAGATGGT
AAGCCCTCCCGTATCGTAGTTATCTACACGACGGGGAGTCAGGCAACTATGGATGAACGAAATAGACAGATCGCT
GAGATAGGTGCCTCACTGATTAAGCATTGGTAACTGTCAGACCAAGTTTACTCATATATACTTTAGATTGATTTA
AAACTTCATTTTTAATTTAAAAGGATCTAGGTGAAGATCCTTTTTGATAATCTCATGACCAAAATCCCTTAACGT
GAGTTTTCGTTCCACTGAGCGTCAGACCCCGTAGAAAAGATCAAAGGATCTTCTTGAGATCCTTTTTTTCTGCGC
GTAATCTGCTGCTTGCAAACAAAAAAACCACCGCTACCAGCGGTGGTTTGTTTGCCGGATCAAGAGCTACCAACT
CTTTTTCCGAAGGTAACTGGCTTCAGCAGAGCGCAGATACCAAATACTGTCCTTCTAGTGTAGCCGTAGTTAGGC
CACCACTTCAAGAACTCTGTAGCACCGCCTACATACCTCGCTCTGCTAATCCTGTTACCAGTGGCTGCTGCCAGT
GGCGATAAGTCGTGTCTTACCGGGTTGGACTCAAGACGATAGTTACCGGATAAGGCGCAGCGGTCGGGCTGAACG
GGGGGTTCGTGCACACAGCCCAGCTTGGAGCGAACGACCTACACCGAACTGAGATACCTACAGCGTGAGCATTGA
GAAAGCGCCACGCTTCCCGAAGGGAGAAAGGCGGACAGGTATCCGGTAAGCGGCAGGGTCGGAACAGGAGAGCGC
ACGAGGGAGCTTCCAGGGGGAAACGCCTGGTATCTTTATAGTCCTGTCGGGTTTCGCCACCTCTGACTTGAGCGT
CGATTTTTGTGATGCTCGTCAGGGGGGCGGAGCCTATGGAAAAACGCCAGCAACGCGGCCTTTTTACGGTTCCTG
GCCTTTTGCTGGCCTTTTGCTCACATGTTCTTTCCTGCGTTATCCCCTGATTCTGTGGATAACCGTATTACCGCC
TTTGAGTGAGCTGATACCGCTCGCCGCAGCCGAACGACCGAGCGCAGCGAGTCAGTGAGCGAGGAAGCGGAAGAG
CGCCCAATACGCAAACCGCCTCTCCCCGCGCGTTGGCCGATTCATTAATGCAGTAATACATAACCTTATGTATCA
TACACATACGATTTAGGTGACACTATAG

>K-9255 ^{S3K64A}rP2X7^{S3}rP2X7^{S3K64A}rP2X7-pNKS4 ($^{ko-wt-ko}$trimer) 216.494 Da, pI 8.29, 1.911 residues

AATACAAGCTTACAACAAAGAACAACAAACAACAAAGTCCGACGTCGAAGTAGCCACCTACCATCATCATCATCA
TCCCATGGGGAGCGCTTGGAGCCACCCGCAGTTCGAAAAAGGTGGAGGTTCTGGCGGTGGATCGGGAGGTTCAGC
GTGGAGCCACCCGCAGTTCGAGAAAGCTCCCGCTTGCTGTAGCTGGAACGATGTCTTTCAGTATGAGACAAACAA
AGTCACCCGGATCCAGAGCGTGAATTACGGCACCATCAAGTGGATCTTGCACATGACAGTCTTTTCCTACGTTAG
CTTTGCTTTGATGAGCGACAAGCTATATCAGCGGAAGGAGCCCCTTATCAGCTCTGTGCACACCGCTGTCAAAGG
CGTTGCAGAGGTGACAGAGAATGTCACGGAGGGCGGGGTGACGAAGTTAGTACACGGCATCTTCGACACGGCCGA
CTACACCCTCCCTTTGCAGGGGAACTCGTTCTTTGTAATGACAAATTATCTCAAGTCAGAAGGCCAAGAACAGAA
GCTGTGTCCTGAGTATCCCAGCCGCGGTAAACAGTGCCATTCTGACCAGGGTTGTATAAAAGGATGGATGGACCC
ACAAAGTAAAGGAATCCAGACCGGCAGGTGTATACCTTACGACCAGAAGAGGAAGACCTGTGAAATCTTTGCCTG
GTGTCCTGCTGAGGAAGGGAAAGAAGCCCCACGGCCTGCACTCTTGAGGAGCGCCGAAAACTTCACCGTACTCAT
CAAGAACAATATCGACTTCCCGGGCCACAACTATACTACGAGAAACATCTTACCAGGTATGAACATCTCTTGTAC
CTTTCACAAGACTTGGAACCCTCAGTGTCCCATCTTCCGGCTAGGGGACATCTTCCAGGAAATCGGAGAGAACTT
TACAGAGGTGGCAGTTCAGGGAGGAATCATGGGCATTGAGATCTACTGGGACTGCAACCTGGACAGCTGGTCCCA
TCGCTGTCAACCCAAATACAGCTTCCGCCGGCTGGACGACAAGTACACCAATGAGTCCCTGTTCCCTGGCTACAA
CTTCAGATACGCCAAGTACTATAAGGAAAATGGCATGGAAAAGCGGACATTGATCAAAGCCTTCGGCGTGCGTTT
TGACATCCTGGTTTTTGGCACTGGAGGAAAGTTTGACATCATCCAGTTGGTTGTGTACATTGGATCCACCCTGTC
CTATTTCGGTTTGGCCACCGTGTGTATTGACTTGATCATCAACACGTATGCCAGTACCTGCTGCAGGTCACGTGT
TTACCCCTCCTGTAAGTGCTGCGAGCCCTGTGCAGTGAATGAGTACTACTACAGAAAGAAGTGTGAGCCCATCGT
GGAGCCCAAGCCGACGTTAAAGTATGTGTCCTTTGTGGACGAGCCCCACATTTGGATGGTGGACCAGCAGCTGCT
TGGGAAAAGTCTGCAAGATGTCAAAGGTCAAGAGGTCCCGAGACCCCAGACGGACTTCTTGGAACTGTCTAGGCT
CTCCCTCTCTCTCCACCACTCACCCCCAATTCCTGGACAACCTGAGGAAATGCAGCTGCTCCAGATAGAAGCGGT
TCCTAGGTCCAGGGACAGCCCAGATTGGTGCCAGTGTGGAAACTGCCTCCCGTCTCAACTACCAGAGAACCGCAG
GGCCCTGGAGGAGCTGTGCTGCCGGAGGAAGCCAGGACAGTGCATCACTACCTCTGAGCTCTTCAGTAAGATCGT
GCTATCCAGAGAGGCCCTGCAGCTCCTCCTGCTCTACCAGGAGCCCTTGCTGGCGCTGGAGGGAGAGGCCATCAA
CAGCAAGCTGCGACACTGTGCGTACAGGAGCTATGCCACCTGGCGCTTTGTCTCCCAAGACATGGCCGACTTTGC
CATTCTGCCCAGCTGCTGCCGCTGGAAGATCCGGAAGGAGTTCCCCAAGACCCAGGGGCAGTACTCTGGCTTCAA
GTATCCCTACGGTGGATCTGGTGGCGGTGGATCAGGAGGCGGTGGATCAGGACTCATGGGGAGCGCTTGGAGCCA
CCCGCAGTTCGAAAAAGGTGGAGGTTCTGGCGGTGGATCGGGAGGTTCAGCGTGGAGCCACCCGCAGTTCGAGAA
AGCTCCCGCTTGCTGTAGCTGGAACGATGTCTTTCAGTATGAGACAAACAAAGTCACCCGGATCCAGAGCGTGAA
TTACGGCACCATCAAGTGGATCTTGCACATGACAGTCTTTTCCTACGTTAGCTTTGCTTTGATGAGCGACAAGCT
ATATCAGCGGAAGGAGCCCCTTATCAGCTCTGTGCACACCAAGGTCAAAGGCGTTGCAGAGGTGACAGAGAATGT
CACGGAGGGCGGGGTGACGAAGTTAGTACACGGCATCTTCGACACGGCCGACTACACCCTCCCTTTGCAGGGGAA
CTCGTTCTTTGTAATGACAAATTATCTCAAGTCAGAAGGCCAAGAACAGAAGCTGTGTCCTGAGTATCCCAGCCG
CGGTAAACAGTGCCATTCTGACCAGGGTTGTATAAAAGGATGGATGGACCCACAAAGTAAAGGAATCCAGACCGG
CAGGTGTATACCTTACGACCAGAAGAGGAAGACCTGTGAAATCTTTGCCTGGTGTCCTGCTGAGGAAGGGAAAGA
AGCCCCACGGCCTGCACTCTTGAGGAGCGCCGAAAACTTCACCGTACTCATCAAGAACAATATCGACTTCCCGGG
CCACAACTATACTACGAGAAACATCTTACCAGGTATGAACATCTCTTGTACCTTTCACAAGACTTGGAACCCTCA
GTGTCCCATCTTCCGGCTAGGGGACATCTTCCAGGAAATCGGAGAGAACTTTACAGAGGTGGCAGTTCAGGGAGG
AATCATGGGCATTGAGATCTACTGGGACTGCAACCTGGACAGCTGGTCCCATCGCTGTCAACCCAAATACAGCTT
CCGCCGGCTGGACGACAAGTACACCAATGAGTCCCTGTTCCCTGGCTACAACTTCAGATACGCCAAGTACTATAA
GGAAAATGGCATGGAAAAGCGGACATTGATCAAAGCCTTCGGCGTGCGTTTTGACATCCTGGTTTTTGGCACTGG
AGGAAAGTTTGACATCATCCAGTTGGTTGTGTACATTGGATCCACCCTGTCCTATTTCGGTTTGGCCACCGTGTG
TATTGACTTGATCATCAACACGTATGCCAGTACCTGCTGCAGGTCACGTGTTTACCCCTCCTGTAAGTGCTGCGA
GCCCTGTGCAGTGAATGAGTACTACTACAGAAAGAAGTGTGAGCCCATCGTGGAGCCCAAGCCGACGTTAAAGTA
TGTGTCCTTTGTGGACGAGCCCCACATTTGGATGGTGGACCAGCAGCTGCTTGGGAAAAGTCTGCAAGATGTCAA
AGGTCAAGAGGTCCCGAGACCCCAGACGGACTTCTTGGAACTGTCTAGGCTCTCCCTCTCTCTCCACCACTCACC
CCCAATTCCTGGACAACCTGAGGAAATGCAGCTGCTCCAGATAGAAGCGGTTCCTAGGTCCAGGGACAGCCCAGA
TTGGTGCCAGTGTGGAAACTGCCTCCCGTCTCAACTACCAGAGAACCGCAGGGCCCTGGAGGAGCTGTGCTGCCG
GAGGAAGCCAGGACAGTGCATCACTACCTCTGAGCTCTTCAGTAAGATCGTGCTATCCAGAGAGGCCCTGCAGCT
CCTCCTGCTCTACCAGGAGCCCTTGCTGGCGCTGGAGGGAGAGGCCATCAACAGCAAGCTGCGACACTGTGCGTA
CAGGAGCTATGCCACCTGGCGCTTTGTCTCCCAAGACATGGCCGACTTTGCCATTCTGCCCAGCTGCTGCCGCTG
GAAGATCCGGAAGGAGTTCCCCAAGACCCAGGGGCAGTACTCTGGCTTCAAGTATCCCTACGGTGGATCTGGTGG
CGGTGGATCAGGAGGCGGTGGATCAGGACTCATGGGGAGCGCTTGGAGCCACCCGCAGTTCGAAAAAGGTGGAGG
TTCTGGCGGTGGATCGGGAGGTTCAGCGTGGAGCCACCCGCAGTTCGAGAAAGCTCCCGCTTGCTGTAGCTGGAA
CGATGTCTTTCAGTATGAGACAAACAAAGTCACCCGGATCCAGAGCGTGAATTACGGCACCATCAAGTGGATCTT
GCACATGACAGTCTTTTCCTACGTTAGCTTTGCTTTGATGAGCGACAAGCTATATCAGCGGAAGGAGCCCCTTAT

CAGCTCTGTGCACACCGCTGTCAAAGGCGTTGCAGAGGTGACAGAGAATGTCACGGAGGGCGGGGTGACGAAGTT
AGTACACGGCATCTTCGACACGGCCGACTACACCCTCCCTTTGCAGGGGAACTCGTTCTTTGTAATGACAAATTA
TCTCAAGTCAGAAGGCCAAGAACAGAAGCTGTGTCCTGAGTATCCCAGCCGCGGTAAACAGTGCCATTCTGACCA
GGGTTGTATAAAAGGATGGATGGACCCACAAAGTAAAGGAATCCAGACCGGCAGGTGTATACCTTACGACCAGAA
GAGGAAGACCTGTGAAATCTTTGCCTGGTGTCCTGCTGAGGAAGGGAAAGAAGCCCCACGGCCTGCACTCTTGAG
GAGCGCCGAAAACTTCACCGTACTCATCAAGAACAATATCGACTTCCCGGGCCACAACTATACTACGAGAAACAT
CTTACCAGGTATGAACATCTCTTGTACCTTTCACAAGACTTGGAACCCTCAGTGTCCCATCTTCCGGCTAGGGGA
CATCTTCCAGGAAATCGGAGAGAACTTTACAGAGGTGGCAGTTCAGGGAGGAATCATGGGCATTGAGATCTACTG
GGACTGCAACCTGGACAGCTGGTCCCATCGCTGTCAACCCAAATACAGCTTCCGCCGGCTGGACGACAAGTACAC
CAATGAGTCCCTGTTCCCTGGCTACAACTTCAGATACGCCAAGTACTATAAGGAAAATGGCATGGAAAAGCGGAC
ATTGATCAAAGCCTTCGGCGTGCGTTTTGACATCCTGGTTTTTGGCACTGGAGGAAAGTTTGACATCATCCAGTT
GGTTGTGTACATTGGATCCACCCTGTCCTATTTCGGTTTGGCCACCGTGTGTATTGACTTGATCATCAACACGTA
TGCCAGTACCTGCTGCAGGTCACGTGTTTACCCCTCCTGTAAGTGCTGCGAGCCCTGTGCAGTGAATGAGTACTA
CTACAGAAAGAAGTGTGAGCCCATCGTGGAGCCCAAGCCGACGTTAAAGTATGTGTCCTTTGTGGACGAGCCCCA
CATTTGGATGGTGGACCAGCAGCTGCTTGGGAAAAGTCTGCAAGATGTCAAAGGTCAAGAGGTCCCGAGACCCCA
GACGGACTTCTTGGAACTGTCTAGGCTCTCCCTCTCTCTCCACCACTCACCCCCAATTCCTGGACAACCTGAGGA
AATGCAGCTGCTCCAGATAGAAGCGGTTCCTAGGTCCAGGGACAGCCCAGATTGGTGCCAGTGTGGAAACTGCCT
CCCGTCTCAACTACCAGAGAACCGCAGGGCCCTGGAGGAGCTGTGCTGCCGGAGGAAGCCAGGACAGTGCATCAC
TACCTCTGAGCTCTTCAGTAAGATCGTGCTATCCAGAGAGGCCCTGCAGCTCCTCCTGCTCTACCAGGAGCCCTT
GCTGGCGCTGGAGGGAGAGGCCATCAACAGCAAGCTGCGACACTGTGCGTACAGGAGCTATGCCACCTGGCGCTT
TGTCTCCCAAGACATGGCCGACTTTGCCATTCTGCCCAGCTGCTGCCGCTGGAAGATCCGGAAGGAGTTCCCCAA
GACCCAGGGGCAGTACTCTGGCTTCAAGTATCCCTACTGACTATCTAGAGGATCCCCGGGTACGTACCGGGCCCC
CCATCGAGGTCGACGTAGCTTTCATTTGTTTT**AA**TTT**A**TTTTTT**AAA**T**A**GC**A**TT**A**C**AAAA**C**AAA**TT**A**CC**A**C**AA**C**A**
AAAAAAAAACC**AAAA**C**AAAAA**C**AAA**T**AA**T**AAA**GTCCC**AAAA**C**AAAAA**CGG**AA**T**A**TGC**AAA**C**AAAAAAAAAAAAAA**
AAAAAAAAAAAAAAAAAAAAAAAAAAAAAAAAAAAAAGAATTGCTCGAGCGGCCGCTCGAGCAATTCCGGTCTCC
CTATAGTGAGTCGTATTACTGGCGTAATAGCGAAGAGGCCCGCACCGATCGCCCTTCCCAACAGTTGCGTAGCCT
GAATGGCGAATGGGACGCGCCCTGTAGCGGCGCATTAAGCGCGGCGGGTGTGGTGGTTACGCGCAGCGTGACCGC
TACACTTGCCAGCGCCCTAGCGCCCGCTCCTTTCGCTTTCTTCCCTTCCTTTCTCGCCACGTTCGCCGGCTTTCC
CCGTCAAGCTCTAAATCGGGGGCTCCCTTTAGGGTTCCGATTTAGTGCTTTACGGCACCTCGACCCCAAAAAACT
TGATTAGGGTGATGGTTCACGTGGGCCATCGCCCTGATAGACGGTTTTTCGCCCTTTGACGTTGGAGTCCACGTT
CTTTAATAGTGGACTCTTGTTCCAAACTGGAACAACACTCAACCCTATCTCGGTCTATTCTTTTGATTTATAAGG
GATTTTGCCGATTTCGGCCTATTGGTTAAAAAATGAGCTGATTTAACAAAATTTAACGCGAATTTTAACAAAATA
TTAACGTTTACAATTTCAGGTGGCACTTTTCGGGGAAATGTGCGCGGAACCCCTATTTGTTTATTTTTCTAAATA
CATTCAAATATGTATCCGCTCATGAGACAATAACCCTGATAAATGCTTCAATAATATTGAAAAAGGAAGAGTATG
AGTATTCAACATTTCCGTGTCGCCCTTATTCCCTTTTTTGCGGCATTTTGCCTTCCTGTTTTTGCTCACCCAGAA
ACGCTGGTGAAAGTAAAAGATGCTGAAGATCAGTTGGGTGCACGAGTGGGTTACATCGAACTGGATCTCAACAGC
GGTAAGATCCTTGAGAGTTTTCGCCCCGAAGAACGTTTTCCAATGATGAGCACTTTTAAAGTTCTGCTATGTGGC
GCGGTATTATCCCGTATTGACGCCGGGCAAGAGCAACTCGGTCGCCGCATACACTATTCTCAGAATGACTTGGTT
GAGTACTCACCAGTCACAGAAAAGCATCTTACGGATGGCATGACAGTAAGAGAATTATGCAGTGCTGCCATAACC
ATGAGTGATAACACTGCGGCCAACTTACTTCTGACAACGATCGGAGGACCGAAGGAGCTAACCGCTTTTTTGCAC
AACATGGGGGATCATGTAACTCGCCTTGATCGTTGGGAACCGGAGCTGAATGAAGCCATACCAAACGACGAGCGT
GACACCACGATGCCTGTAGCAATGGCAACAACGTTGCGCAAACTATTAACTGGCGAACTACTTACTCTAGCTTCC
CGGCAACAATTAATAGACTGGATGGAGGCGGATAAAGTTGCAGGACCACTTCTGCGCTCGGCCCTTCCGGCTGGC
TGGTTTATTGCTGATAAATCTGGAGCCGGTGAGCGTGGGTCTCGCGGTATCATTGCAGCACTGGGGCCAGATGGT
AAGCCCTCCCGTATCGTAGTTATCTACACGACGGGGAGTCAGGCAACTATGGATGAACGAAATAGACAGATCGCT
GAGATAGGTGCCTCACTGATTAAGCATTGGTAACTGTCAGACCAAGTTTACTCATATATACTTTAGATTGATTTA
AAACTTCATTTTTAATTTAAAAGGATCTAGGTGAAGATCCTTTTTGATAATCTCATGACCAAAATCCCTTAACGT
GAGTTTTCGTTCCACTGAGCGTCAGACCCCGTAGAAAAGATCAAAGGATCTTCTTGAGATCCTTTTTTTCTGCGC
GTAATCTGCTGCTTGCAAACAAAAAAACCACCGCTACCAGCGGTGGTTTGTTTGCCGGATCAAGAGCTACCAACT
CTTTTTCCGAAGGTAACTGGCTTCAGCAGAGCGCAGATACCAAATACTGTCCTTCTAGTGTAGCCGTAGTTAGGC
CACCACTTCAAGAACTCTGTAGCACCGCCTACATACCTCGCTCTGCTAATCCTGTTACCAGTGGCTGCTGCCAGT
GGCGATAAGTCGTGTCTTACCGGGTTGGACTCAAGACGATAGTTACCGGATAAGGCGCAGCGGTCGGGCTGAACG
GGGGGTTCGTGCACACAGCCCAGCTTGGAGCGAACGACCTACACCGAACTGAGATACCTACAGCGTGAGCATTGA
GAAAGCGCCACGCTTCCCGAAGGGAGAAAGGCGGACAGGTATCCGGTAAGCGGCAGGGTCGGAACAGGAGAGCGC
ACGAGGGAGCTTCCAGGGGGAAACGCCTGGTATCTTTATAGTCCTGTCGGGTTTCGCCACCTCTGACTTGAGCGT
CGATTTTTGTGATGCTCGTCAGGGGGGCGGAGCCTATGGAAAAACGCCAGCAACGCGGCCTTTTTACGGTTCCTG
GCCTTTTGCTGGCCTTTTGCTCACATGTTCTTTCCTGCGTTATCCCCTGATTCTGTGGATAACCGTATTACCGCC
TTTGAGTGAGCTGATACCGCTCGCCGCAGCCGAACGACCGAGCGCAGCGAGTCAGTGAGCGAGGAAGCGGAAGAG

CGCCCAATACGCAAACCGCCTCTCCCCGCGCGTTGGCCGATTCATTAATGCAGTAATACATAACCTTATGTATCA
TACACATACGATTTAGGTGACACTATAG
