# Supplementary material for: Activation of the Rat P2X7 Receptor by Functionally Different ATP Activation Sites
Source: Cells. 2025 Jun 6;14(12):855. doi: 10.3390/cells14120855 (PMC12191118; doi:10.3390/cells14120855)
Supplement: Supplementary file 1 [file cells-14-00855-s001.zip › Supplementary Table 2 (ARRIVE guidelines).pdf]

**Supplementary Table S2. Compliance with the ARRIVE guidelines for using *Xenopus laevis* as donors of isolated oocytes for in vitro expression experiments.**

1. Study Design

**ARRIVE Item:** Briefly describe the study design, including the experimental groups and controls.

**Response:** Our study involved cRNA injections into *Xenopus laevis* oocytes that were surgically removed under tricaine anesthesia performed, in accordance with approved protocols. The oocytes were then injected ex vivo with cRNA or control solutions. They were kept at ambient temperature for 2-3 days and then analyzed for functional responses and biochemical expression. No behavioral or survival endpoints in live animals were assessed. All of the *Xenopus laevis* survived the surgery and recovered.

---

2. Sample Size

**ARRIVE Item:** Explain how the sample size was determined.

**Response:** The removed partial ovary contained a sufficient number of oocytes for several experiments. The sample size was determined based on previous in vitro studies using *Xenopus* oocytes, in order to ensure sufficient statistical power for the electrophysiological measurements. For biochemical experiments, 10-15 oocytes were typically used to allow for the selection of a sufficient number of healthy oocytes for the experiment, as well as for repeat PAGE experiments in case of technical problems. Due to the in vitro nature of the experiments, no formal power calculation was performed.

---

3. Inclusion and Exclusion Criteria

**ARRIVE Item:** Describe criteria for including/excluding animals or data.

**Response:** Prior to their surgical removal, there was no obvious sign that allowed us to assess the quality of the oocytes. Only healthy oocytes could be used in the experiment. Those that were damaged during isolation or did not survive the injection procedure were excluded from further analysis.

---

4. Randomization

**ARRIVE Item:** State whether randomization was used.

**Response:** The frogs were not randomly selected; rather, they were chosen at the examiners' discretion. After isolation, the frogs were randomly allocated to experimental or control groups. Due to the in vitro design, no further randomization was necessary..

---

5. Blinding

**ARRIVE Item:** State whether experimenters were blinded to group allocation.

**Response:** Blinding was not performed as all experimental manipulations and analyses were conducted in vitro by the same investigator, and group assignments were evident from the experimental setup.

---

6. Outcome Measures

**ARRIVE Item:** Clearly define all outcome measures.

**Response:** The primary outcome measures were the biochemical expression and the electrophysiological response of oocytes after cRNA injection. These were assessed biochemically and electrophysiologically, using SDS-PAGE and PAGE techniques and two-electrode voltage clamp recordings, respectively.

---

7. Statistical Methods

**ARRIVE Item:** Describe statistical methods used.

**Response:** The in vitro data were analyzed using as the appropriate statistics, as detailed in the paper.

---

8. Experimental Animals

**ARRIVE Item:** Provide details of animals used.

**Response:** Adult female *Xenopus laevis* frogs were obtained from certified breeders. Age and weight were not recorded specifically, since only mature females were used for oocyte collection.

---

9. Housing and Husbandry

**ARRIVE Item:** Describe housing and husbandry conditions.

**Response:** In accordance with institutional guidelines, the frogs were housed in aquaria with a controlled temperature (18–20°C) and light-dark cycles. They were provided with clean water and regular feedings.

---

10. Animal Care and Monitoring

**ARRIVE Item:** Describe any interventions to reduce pain, suffering, or distress.

**Response:** Ovariectomy was performed under tricaine immersion anesthesia to minimize pain and distress, in accordance with approved protocols. The incisions were carefully closed with sutures using professional surgical instruments. The female frogs were allowed to recover for one to two days in individual baths before being returned to their group.

---

11. Ethical Statement

**ARRIVE Item:** Provide details of ethical review and approval.

**Response:** All procedures were approved by the local animal welfare committees in Halle, Germany (reference no. Az. 203.42502-2-1493 MLU) and Düsseldorf, Germany (reference no. 8.87-51.05.20.10.131), and were conducted in accordance with EC Directive 86/609/EEC.

---

12. Generalizability/Translation

**ARRIVE Item:** Discuss how findings might translate to other systems/species.

**Response:** Because the oocytes were injected with cRNAs encoding mammalian proteins, the resulting data reflect the primary biochemical expressions and functional characteristics in mammals.

---

13. Protocol Registration

**ARRIVE Item:** Was the protocol pre-registered?

**Response:** In Germany, pre-registration of the experimental protocol for ex vivo experiments in isolated *Xenopus laevis* oocytes is not required by regulation.

---

14. Data Accessibility

**ARRIVE Item:** State where data supporting the findings can be accessed.

**Response:** All data supporting the findings of this study are presented in the figures included in the paper. Additionally, the dataset used for the statistical analysis will be deposited in the Open Access Research Data Repository, Share_it, of the University Libraries of Saxony-Anhalt.

---

For Items Not Applicable

For items that do not apply (e.g., animal welfare monitoring during long-term studies, humane endpoints, etc.), you can write:

Not applicable. No live animal procedures or survival endpoints were involved; only in vitro experiments on isolated oocytes were performed.

---

**Summary Table for ARRIVE Responses**

| **ARRIVE Item** | **Example Response (for in vitro oocyte study)** |
|---|---|
| Study Design | In vitro study using *Xenopus laevis* oocytes; no live animal endpoints. |

| ARRIVE Item | Example Response (for in vitro oocyte study) |
|---|---|
| Sample Size | Based on prior studies; no formal power calculation. |
| Inclusion Criteria | Healthy stage V–VI oocytes only. |
| Randomization | Oocytes randomly allocated after isolation. |
| Blinding | Not performed; single investigators, in vitro setup. |
| Outcome Measures | Biochemical expression and electrophysiological responses after cRNA injection |
| Statistics | One-way ANOVA, multiple t-tests with Bonferroni correction using Sigma Plot; $p < 0.05$. |
| Animals | Adult female *Xenopus laevis*; mature females only. |
| Housing | Aquaria, 18–20°C, light-dark cycle, fresh water |
| Animal Care | Ovariectomy under tricaine anesthesia. |
| Ethical Statement | Approved by local committees in Halle and Düsseldorf; EC Directive 86/609/EEC. |
| Generalizability | In vitro findings; direct translation possible due to the expression of mammalian proteins |
| Protocol Registration | Not pre-registered. |
| Data Accessibility | Open Access Research Data Repository “Share_it”, University Libraries of Saxony-Anhalt |
| Not Applicable Items | Not applicable; only in vitro experiments performed. |
